# Deformation and Failure of Supported Five-Fold Twinned Silver Nanowires under Tensile, Compressive, and Cyclic Loading

**DOI:** 10.1021/acsnano.5c05537

**Published:** 2025-08-21

**Authors:** Marco Moninger, Lilian M. Vogl, Peter Schweizer, Peter Denninger, Patricia Smolka, Johannes Will, Erdmann Spiecker

**Affiliations:** Institute of Micro- and Nanostructure Research (IMN) and Center for Nanoanalysis and Electron Microscopy (CENEM), Interdisciplinary Center for Nanostructured Films (IZNF), Friedrich-Alexander-Universität Erlangen-Nürnberg, Cauerstrasse 3, 91058 Erlangen, Germany

**Keywords:** silver nanowire, flexible transparent electrode, mechanical testing, nanowire deformation and failure, correlative light and electron microscopy

## Abstract

Silver nanowire (AgNW) networks have emerged as one of the most promising materials for flexible transparent conductive electrodes. These wires offer excellent electrical, optical, and mechanical properties and can be applied using low-cost printing techniques with the potential for upscaling. To elucidate the mechanical properties of nanowire networks for use in flexible electronics, it is essential to first characterize the behavior of individual wires adhered to the polymer surface under mechanical loading of the polymer. This study investigates the mechanical response of isolated nanowires during uniaxial in situ tensile testing of the polymer using correlative microscopy, which combines the advantages of light and electron microscopy. By changing the orientation of the nanowires with respect to the tensile straining axis of the polymer, the nanowires experience either tensile (for parallel orientation) or compressive forces (for perpendicular orientation) according to the polymer’s elastic–plastic Poisson’s ratio, which links lateral contraction of the polymer to tensile strain. Aligned and isolated AgNWs were applied to flat surfaces of two polymers, PET and PDMS, which serve as model systems to investigate the effect of the substrate on the mechanical response of the nanowires. We observe a strong influence of the polymer type on the wire deformation behavior and fracture, which we attribute to the different adhesion strength of the wires on PET and PDMS. While the wires on PET undergo multiple fractures, breaking into segments of roughly equal length under tensile loading, those on PDMS typically fracture only once, accompanied by early sliding of the wire on the substrate. Compression tests revealed localized plastic deformation by nanowire kinking with the formation of new grain boundaries for both polymer substrates. Electron microscopy studies revealed different deformation configurations depending on the amount of load applied. In addition, cyclic compressive tests provided insight into the fatigue behavior of the wires. Here, newly formed grain boundaries acted as potential fracture sites, whereas the purely elastic deformation remained fully reversible up to 1000 cycles.

## Graphical abstract

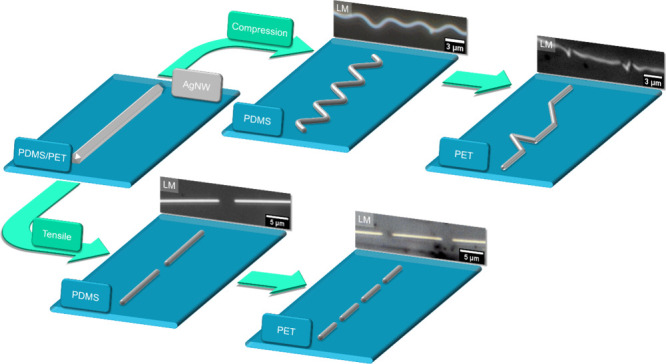

Percolation-based networks using nanowires as building blocks are one of the most promising alternatives to indium tin oxide (ITO) for use as transparent conductive electrodes (TCEs),^1,2^ which are essential components in thin film technologies such as organic solar cells,^3,4^ light-emitting diodes,^5,6^ and sensors.^7,8^ The use of ITO inherently restricts the flexibility of such devices because of its brittle nature.^9,10^ Moreover, ITO contains >70 wt % Indium, a rare and limited resource. In the field of network electrodes, different types of wires have been investigated ranging from carbon nanotubes (CNTs) to metallic nanowires.^11,12^ Among these, five-fold twinned AgNWs stand out as the most promising candidate due to well-established synthesis methods that yield highly monodisperse nanowires with a high aspect ratio.^13,14^ Additionally, they offer an optimal combination of electrical, optical and mechanical properties, making them highly suitable for TCEs and device design requirements.^15–19^ The two major requirements for TCEs are an optical transmittance of ∼90% and a sheet resistance in the range of 100 Ω/sq, depending on the application.^20^ To prove the mechanical resilience of network electrodes, bending tests have been largely adopted as the standard testing method.^16,21^ Depending on the testing setup and substrate thickness, bending tests induce only a relatively low amount of strain in the network, typically in the range of 1–2%, which is not sufficient to result in substantial fracture of the network and its wires.^9,22,23^ To reach higher strain levels tensile straining tests have been conducted revealing facture of individual wires in the network at tensile strains of 3–4% accompanied by an increase in sheet resistance.^24,25^ Combined with microscopic investigations, these tests have provided direct microscopic insight into the mechanical deformation and failure of the network. However, they do not directly reveal the intrinsic mechanical behavior of individual nanowires on polymer substrates, as the numerous wire intersections significantly influence their mechanical response. To get insight into the deformation and fracture characteristics of individual nanowires, tensile tests on freestanding nanowires can be employed. When combined with different imaging techniques such as scanning electron microscopy (SEM) or transmission electron microscopy (TEM), these tests provide valuable insights into the nanowires’ elastic (e.g., Young’s modulus) and plastic (e.g., tensile strength, elongation at break) properties, as well as the microscopic defect processes that lead to wire failure.^26–31^

Yin et al. have shown that single crystalline AgNWs deform either through twinning or localized dislocation slip, depending on the aspect ratio of the wire’s cross section.^32^ However, the more commonly used five-fold twinned AgNWs show a significantly different deformation and fracture behavior. During tensile straining, five-fold twinned AgNWs initially exhibit defect nucleation along their entire length before the deformation localizes, initiating the formation of a neck. As result of stress localization the wire eventually fractures in the region of the neck, leaving tapered ends indicative of the ductile nature of the fracture event.^24,26,29^ This behavior can be attributed to the wire’s unique microstructure, consisting of five interconnected twin boundaries that act as barriers for defect propagation. Depending on the wire diameter, this results in size-dependent strain hardening and ductility of the five-fold twinned AgNWs.^33,34,24,35,36^ The presence of interconnected twin boundaries also influences the deformation behavior of five-fold twinned AgNWs under compression and bending. In this context, it is important to note that nanowires with small diameter and high aspect ratios typically undergo bending under compressive loading, as the wire eventually deflects from its initially straight configuration.^29,37^ As the bending angle increases, partial dislocations initially nucleate forming stacking faults on the tensile side of the nanowire, followed by formation of full dislocations on the compressive side, as demonstrated by molecular dynamics (MD) simulations.^24^ However, due to the presence of twin boundaries, these dislocations, along with their associated stacking faults, cannot propagate through the entire nanowire or annihilate with each other. Instead, they accumulate and organize into a grain boundary-like structure, forming a kink in the nanowire^24^ Early in situ bending tests of five-fold twinned Ag nanowires conducted in a scanning electron microscope have shown that such kinks can lead to nanowire fracture upon further bending.^38^

In contrast to the tensile or compressive testing of isolated nanowires in SEM or TEM, nanowires used in flexible electronics, e.g., as TCSs, are not free-standing but are instead applied to the surface of a flexible substrate. The introduction of surface interactions can heavily influence the mechanical behavior of a material system, a phenomenon that has been extensively studied in the context of thin metal films on polymer substrates.^39^ Here, varying behavior can be observed depending on the specific film material used. For less ductile metals such as chromium (Cr) and titanium (Ti), as well as brittle oxides like ITO, fracture occurs at very low levels of tensile strain applied to the polymer substrate (<1–2%).^40^ As the strain increases, new cracks continue to form until a periodic crack pattern with a characteristic minimum spacing is established. In contrast, thin films composed of highly ductile fcc metals such as gold (Au), silver (Ag) or copper (Cu) exhibit distinctly different deformation behavior.^39^ These films undergo ductile deformation already at low levels of applied strain, leading to necking or localized deformation beyond a certain onset strain. With further increase in applied strain, complete fracture can occur; however, not all necked regions evolve into through-thickness cracks that span the entire film.^39,41^ Still, a periodically spaced damage is recorded in these systems. Periodic deformation and fracturing have also been observed in five-fold twinned AgNWs on polymer substrates when the polymer is subjected to strain.^24^ However, these observations were made on nanowire networks, where nanowire nodes obscure the intrinsic mechanical response of the individual nanowires.

In this work, we report on a systematic study of the deformation and failure mechanisms of isolated five-fold twinned AgNWs supported on different polymer substrates and exposed to tensile, compressive and cyclic loading. For this purpose, isolated nanowires were prepared on either Polyethylene terephthalate (PET) or Polydimethylsiloxane (PDMS) using doctor blading of an appropriate nanowire solution. The polymer substrates with the wires on top were subjected to tensile straining using custom-build manual and electrical straining stages. The setup makes use of the lateral constriction of the polymer, which enables the application of tensile and compressive load to the nanowires depending on the nanowire orientation. The straining stages are designed to fit under a light microscope (LM) as well as in the SEM enabling in situ imaging of the straining process. By correlating in situ LM with ex situ SEM and TEM, we leverage the advantages of LM—such as compatibility with a realistic environment and the absence of beam damage—while benefiting from the high-resolution capabilities of electron microscopy (EM) techniques. Most importantly, our studies revealed the formation of equidistant cracks (in tension) and variably shaped kinks (in compression), both influenced by the type of substrate. These kinks are characterized by the formation of new grain boundaries (GBs), allowing the nanowire to accommodate high levels of strain. Moreover, using PDMS as a substrate enables cyclic testing of AgNWs, allowing for the investigation of cyclic fatigue and the role of plastic deformation events in triggering nanowire failure. Altogether, our comprehensive study provides detailed microscopic insights into the deformation modes of single AgNWs under various loading scenarios and the pivotal role of adhesion which is key information for the design of nanowire electrodes for flexible and stretchable applications.

## Results and Discussion

### Correlative Approach

To investigate the mechanical properties of five-fold twinned AgNWs on polymer substrates, a correlative approach was chosen.^42^ This approach integrates the advantages of different microscopy techniques, such as LM and EM, and provides a scale bridging perspective on the mechanical response of the supported nanowires. Figure 1 illustrates the correlative workflow, including two loading scenarios and the applied microscopy techniques. All straining tests were conducted with the help of custom-build manual or electrical straining stages which are described in more detail in the Methods sections and the SI. The setups enable both tensile and compressive testing of the nanowires depending on the wire orientation on the substrate. The polymer elongates along the straining direction but contracts in perpendicular direction due to its elastic-plastic Poisson’s ratio (see SI). Under conditions of strong adhesion of the wire on the substrate the strain applied to the substrate is transferred to the wire. In consequence, the type of loading experienced by the wire depends on its alignment to the macroscopic straining direction as visualized in Figure 1. The loading mode is indicated by the arrows for differently oriented wires. Further, the alignment is controlled by an established doctor blade coating approach.^24^ Details and parameters for coating and alignment of the nanowires can be found in the Methods sections.

**Figure 1:**
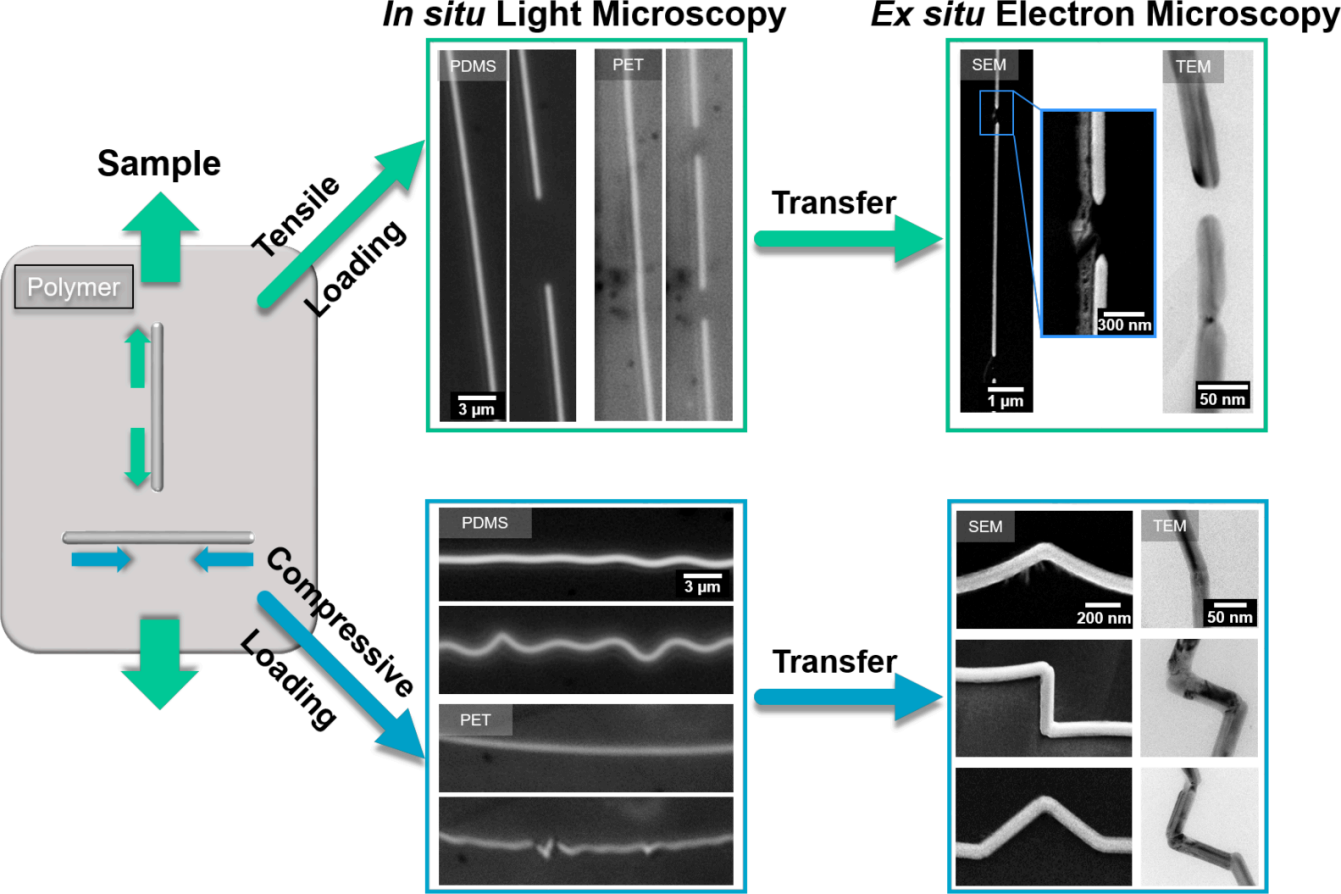
Schematic illustration of the correlative workflow employed in this study, combining light and electron microscopy to elucidate the deformation and failure mechanisms of AgNWs. The schematic on the left illustrates the macroscopic tensile straining of the polymer and the resulting microscopic tensile/compressive loading of the nanowires depending on their vertical/horizontal alignment. Upper row shows wire fracturing under tensile loading in LM, SEM, and TEM images. Bottom row shows nanowire buckling and kinking under compressive deformation in LM, SEM, and TEM images.

The influence of the support-wire interaction was investigated by comparing two different substrates, PET and PDMS. PET is a commonly used substrate material for flexible electronics,^43,44^ which undergoes plastic deformation at relatively small strains. In addition to PET, a variety of other polymer substrates are employed in flexible electronics.^59–61^ We selected PDMS as the second substrate because it is an elastomer that remains elastic even under large strains.^45^ Consequently, straining is reversible, enabling cyclic mechanical loading of the nanowires, which represents a realistic loading scenario for TCEs. As illustrated in Figure 1 and further discussed in the following sections, the mechanical response of the wires is strongly influenced by the substrate material, primarily due to differences in adhesion strength.

### Benefits of Combining Light and Electron Microscopy

Particular attention should be given to the use of LM when investigating nanoscale objects, especially those situated on substrates. Previous in situ studies by our group have demonstrated the capability of LM not only to visualize nanowire growth and quantify their growth kinetics^46^ but also to reveal and analyze the resonant behavior of vibrating nanowires in various gaseous environments.^42^ As shown in the images in Figure 1, LM not only enables imaging of nanowires with a diameter around 50 nm (see Figure S8) during in situ straining, but also allows for resolving changes in wire morphology during deformation and fracturing. At a first glace this might be surprising since according to the Rayleigh criterion the wire diameters are below the resolution limit of the microscope. The criterion states that two objects can be resolved separately when the first diffraction minimum of one object coincides with the maximum of the second object.^47^ However, this does not contradict the imaging of isolated nanowires with diameters significantly below the resolution limit. Due to the diffraction limit, they simply appear wider than they actually are. It also helps that only one dimension of the nanowires—their width—falls below the resolution limit of LM, while their lengths typically extend to tens of microns. As will be shown below, LM images of nanowires are surprisingly sensitive to deformation events, such as nanowire necking or kink formation, even if the microscopic details of these deformations are not resolved. To capture those fine structural details, EM methods must be employed.

The use of LM offers several significant advantages over EM. Most notably, it allows for a flexible choice of environmental conditions—such as ambient pressure—eliminating the need for vacuum and enabling more realistic testing scenarios. Additionally, the interaction between visible light and the sample is largely nondestructive. In contrast, EM requires a vacuum environment, and the electron beam can damage sensitive materials, especially polymers.^48,49^ It can also lead to unwanted carbon deposition,^50^ which may locally alter the mechanical properties of the nanowires.

Nevertheless, due to its high spatial resolution the use of EM is indispensable to gain further insight into the structural changes of the wires during deformation and fracturing (see Figure 1). Due to the interaction between the electron beam and the sample, these investigations were carried out ex situ to identify and further evaluate the phenomena observed in the LM. Here, SEM enabled an analysis of the different deformation events and corresponding geometries formed during compression and also provided a closer look at the cup and cone structure appearing in the tensile fracture case. In addition, TEM enabled a thorough investigation of the defect structures generated during plastic deformation of the wires giving insight into the deformation mechanism of the wires down to the atomic scale.

### Tensile Loading on PDMS and PET

As previously stated, depending on their alignment relative to the initial straining direction, the nanowires are subjected to either tensile or compressive loading. The results of alignment (approximately) parallel to the macroscopic straining direction—corresponding to tensile straining—are summarized in Figure 2. The series of LM images in Figure 2a shows a nanowire on PDMS at different levels of applied strain. The first fracture event occurs at 3.28% substrate strain. With continued straining, the fracture gap widens, indicating slippage of the wire on the PDMS to compensate the strain. No additional fracture events are observed up to the maximum applied macroscopic strain of 15.09%. Figure 2b presents a corresponding series of LM images of a wire on PET. Similar to the PDMS case, the first fracture event occurs at 2.73% applied tensile strain. However, unlike PDMS, additional fracture events occur shortly thereafter at a slightly higher strain level of 3.47%. As a result, all fracture events occur almost simultaneously in a very narrow strain window. Notably, the fracturing leads to fragmentation of the wire in similar sized segments (9.16 ± 1.58 μm). This value was determined by evaluating the nanowire along its full length, which fractured into 13 segments, as shown in Figure S4 of the Supporting Information. Once fragmentation is complete, further straining leads to opening of the fracture gaps, similar to the case of PDMS. Additional images and data for all tensile tests performed on PET and PDMS are provided in the Supporting Information (see Tables S2 and S3, along with the referenced LM image series).

**Figure 2:**
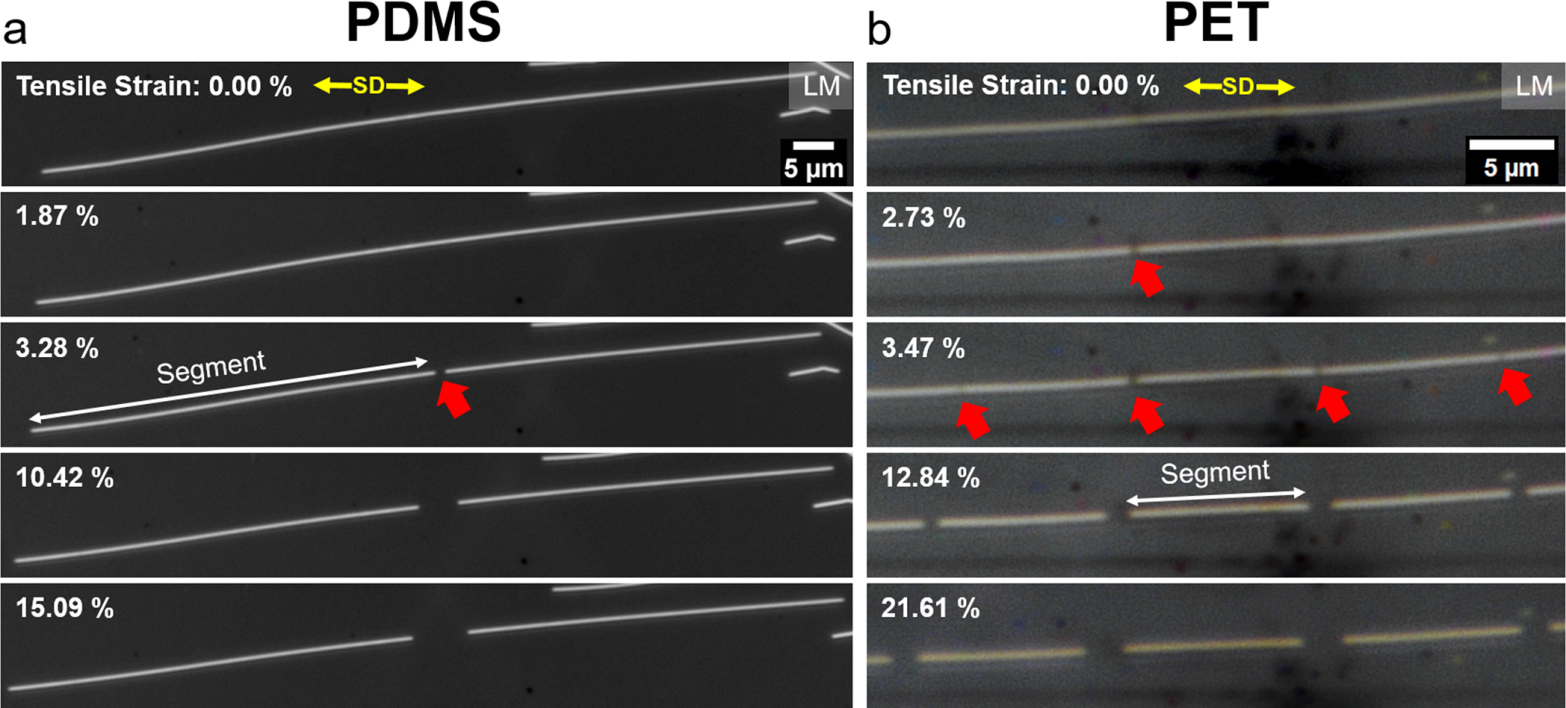
LM still frames from in situ tensile tests with the applied tensile strain indicated in each frame. The yellow arrows indicate the substrate straining direction (SD). (a) AgNW on a PDMS substrate showing failure via single fracture and subsequent opening of the fracture gap. The red arrow indicates the point of fracture. (b) AgNW on the PET substrate exhibiting multiple fractures at approximately equidistant positions along the wire leading to fragmentation with a segment length of 9.16 ± 1.58 μm. Red arrows indicate the initial occurrence of individual failure points, all appearing in a narrow strain window. An LM image series showing the entire nanowire and its fragmentation into 13 wire segments is provided in Figure S4 of the Supporting Information.

To quantify the influence of the substrate on the mechanical response of the wire the total elongation of the wire (including fracture gaps) and the mean strain of individual wire segments formed upon fragmentation of the wires are measured from LM images as a function of strain applied to the substrate. The resulting data are shown in Figure 3. To obtain accurate data a feature tracking algorithm has been used which enables tracking of features in the LM image series with subpixel accuracy. Distinct features on the substrate have been selected for evaluation of the substrate strain whereas wire ends have been used for evaluation of the total wire elongation and the mean strain of wire segments. The whole procedure is described in more detail in the Methods section.

**Figure 3:**
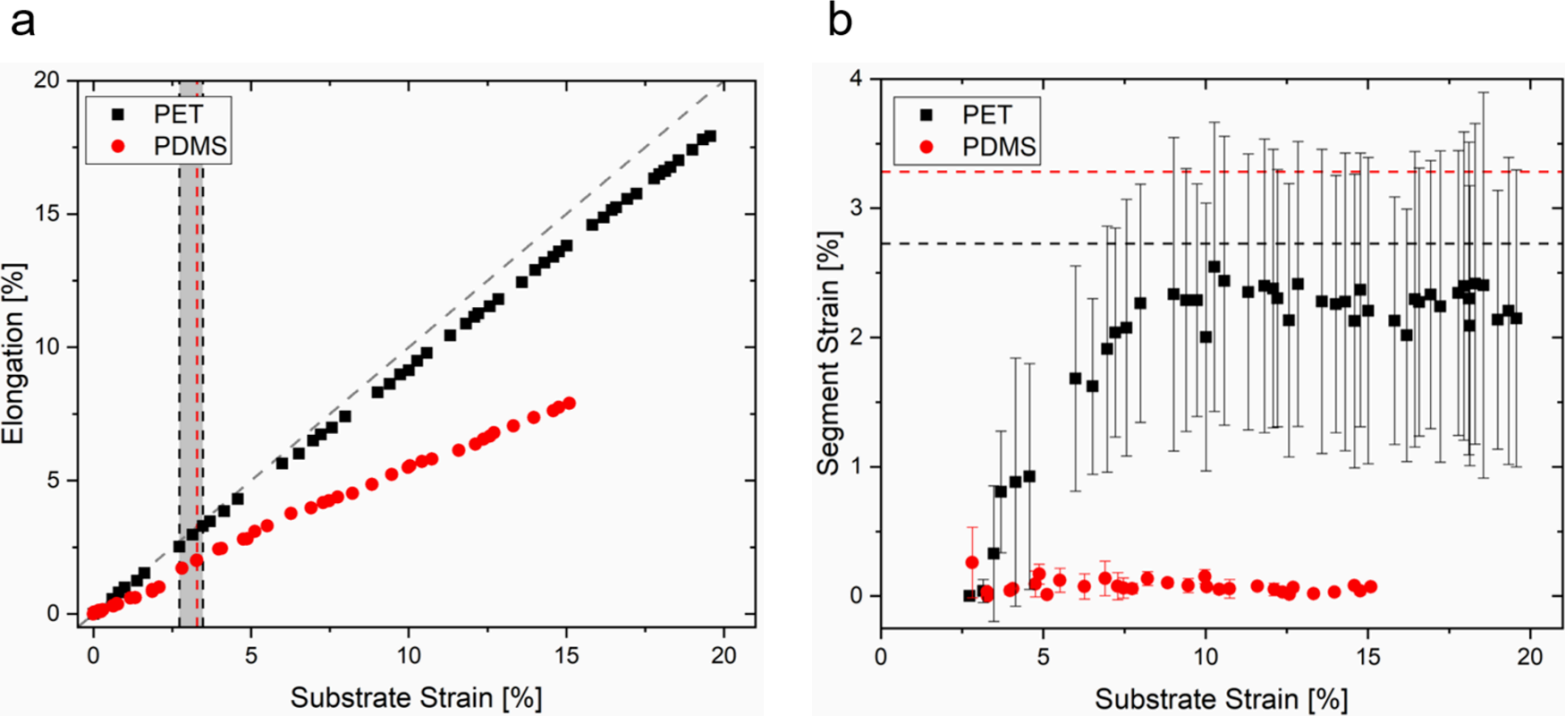
Graphs showing elongation and strain values of the polymer and wires extracted from the in situ tests for PDMS and PET in Figure 2. All values have been extracted from images via feature tracking. (a) Total wire elongation (including fracture gaps) versus substrate strain. Red dashed vertical line indicates singular fracture of wire on PDMS. Gray shaded area indicates strain range at which the segmentation for the wire on PET occurs. The dashed reference line with a slope of 1 indicates complete transfer of the substrate strain to the wire. (b) Evolution of mean strain of the individual wire segments after their formation as a function of increasing substrate strain. Dashed horizontal lines indicate the substrate strain level at which the corresponding complete wire showed the first fracture.

Figure 3a shows the total wire elongation measured from one end to the other as a function of substrate strain. Here, the elongation includes fracture gaps and therefore does not directly translate to actual tensile strain of the wire which is partly relaxed by wire fracturing and subsequent opening of the fracture gaps. The data for PET and PDMS exhibit markedly different behaviors, indicating a pronounced substrate effect. For PET the total elongation very closely follows the substrate stain (dashed diagonal line) indicating a strong adhesion of the wire to the polymer and very limited slipping of the wire ends. In contrast, the data for PDMS deviate significantly from the diagonal, indicating pronounced wire slippage on the substrate—suggestive of lower adhesion between the wire and the polymer. This is in line with the observation in Figure 2a that the wire on PDMS only fractured once. Obviously, further substrate straining does not lead to sufficient buildup of strain in the wire to initiate subsequent fracture events in the two wire segments but is rather accommodated by slipping of the wire ends and opening of the fracture gap. For the wire on PET this route of strain relaxation is only effective for smaller wire segments formed by multiple fracture events (Figure 2b) indicating an almost complete strain transfer from substrate to wire as result of stronger adhesion.

This interpretation is further manifested by the analysis depicted in Figure 3b showing the evolution of the mean strain (or elongation) of the individual wire segments from their first formation to the maximum substrate strain applied which amounts to ∼15% for PDMS and ∼19% for PET. A comparison of the two data sets reveal clear differences in wire-substrate interaction following the initial wire fracture. For PET, each of the 13 tracked segments (see Figure S4) accumulate an average strain of ∼2.5% after the initial fracture, before reaching a plateau. The strain level at the plateau remains below the 2.87% strain value at which the first fracture event occurred (see Figure 2b), as indicated by the dashed horizontal line in Figure 3b. This can be interpreted as follows: upon reaching the plateau, the slipping of the short wire segments at both ends becomes sufficient to accommodate the additional strain applied to the substrate. As a result, no additional strain is transferred from the substrate to the wire, preventing the individual wire segments from reaching the critical strain level required to initiate further fracture events. The situation is completely different for PDMS. Here, hardly any increase in strain of the wire segments can be observed after their formation in the first and only fracture event. This further confirms the much easier slipping of the wires on PDMS, allowing for near-complete relaxation of wire strain—even for the relatively long wire segments. Overall, a stronger surface interaction between the wire and the PET is observed leading to a fragmentation of the initial wire into small segments followed by a buildup of strain in the individual segments. The weaker surface interaction on PDMS leads to slipping of the wire on the substrate surface after a singular fracture event.

The observed fragmentation of the wires on PET shows parallels to the behavior of thin metallic/oxide films on substrates.^39,51^ For less ductile (non-fcc) metals such as Cr or Ti the thin film exhibits an onset of crack formation at strains around 1–2%.^52^ With increasing strain more and more cracks form until a saturation value is reached. Highly ductile materials like Cu show the onset of plastic deformation, which means not fully formed through cracks, at similar strain values. During the straining test many but not all of these deformations start to evolve into full cracks leaving the ductile sample also with a saturation number of cracks.^41^ The investigated wires exhibit a similar behavior to these metallic thin films in that they also reach a saturation crack spacing especially on PET. In contrast to the thin film, the nanowires reach the point of saturation within a very short-range of strains. Furthermore, the crack spacing can give information about the interaction between a thin film and the polymer. This behavior is typically represented by shear stress. The concept is that, beyond a certain load, the adhesion between the film and the substrate becomes insufficient to fully transfer the load to the thin film, ultimately leading to delamination.^40^

The model typically used for this purpose is the shear-lag model.^53^ However, due to the very specific characteristics of the cracking behavior—such as cracks consistently forming at the center of each segment—this model cannot be directly applied to the nanowire case. Nonetheless, the different behavior of the wires on the two substrates can be attributed to variations in surface interaction. This is already evident from the images in Figure 2: while only a single crack appears on PDMS, fragmentation into many wire segments is observed on PET. This suggests a weaker surface interaction between the wire and PDMS, as the adhesion is insufficient to transfer enough strain from the substrate to the wire to initiate additional crack formation.

### Compressive Loading on PET

When the wires are aligned perpendicular to the initial straining direction, a compressive load is imparted through the contraction of the polymer, governed by its elastic-plastic Poisson’s ratio. For the PET used in this study the Poisson’s ratio is approximately 0.4. During compressive loading, the wires do not fracture but instead exhibit distinct deformation events (see Figure 4). While the presence of complete wire fracture with segment separation in the deformed region cannot be ruled out based on the LM images, this is clearly refuted by post-mortem SEM analysis, which confirms that all examined wires remained continuous despite significant deformation (see Figure 5). In addition, the SEM images confirm at higher resolution what is already apparent in the LM images, namely that sharp kinks form at the deformation sites along the wire. Furthermore, we can exclude out-of-plane buckling as an additional mode of strain relaxation, as it would produce distinctly different contrast features in the LM images. This is evidenced by a single compression test on PDMS, which exhibits weaker wire adhesion compared to PET and where out-of-plane buckling was observed. The corresponding LM image series is shown in Figure S10. Out-of-plane buckling is characterized by periodic bright-dark contrast features, while the wire itself remains straight in plan-view projection. At higher strains, the out-of-plane buckling transitions to in-plane buckling and kinking, restoring the wire’s typical uniform white appearance along its entire length.

**Figure 4:**
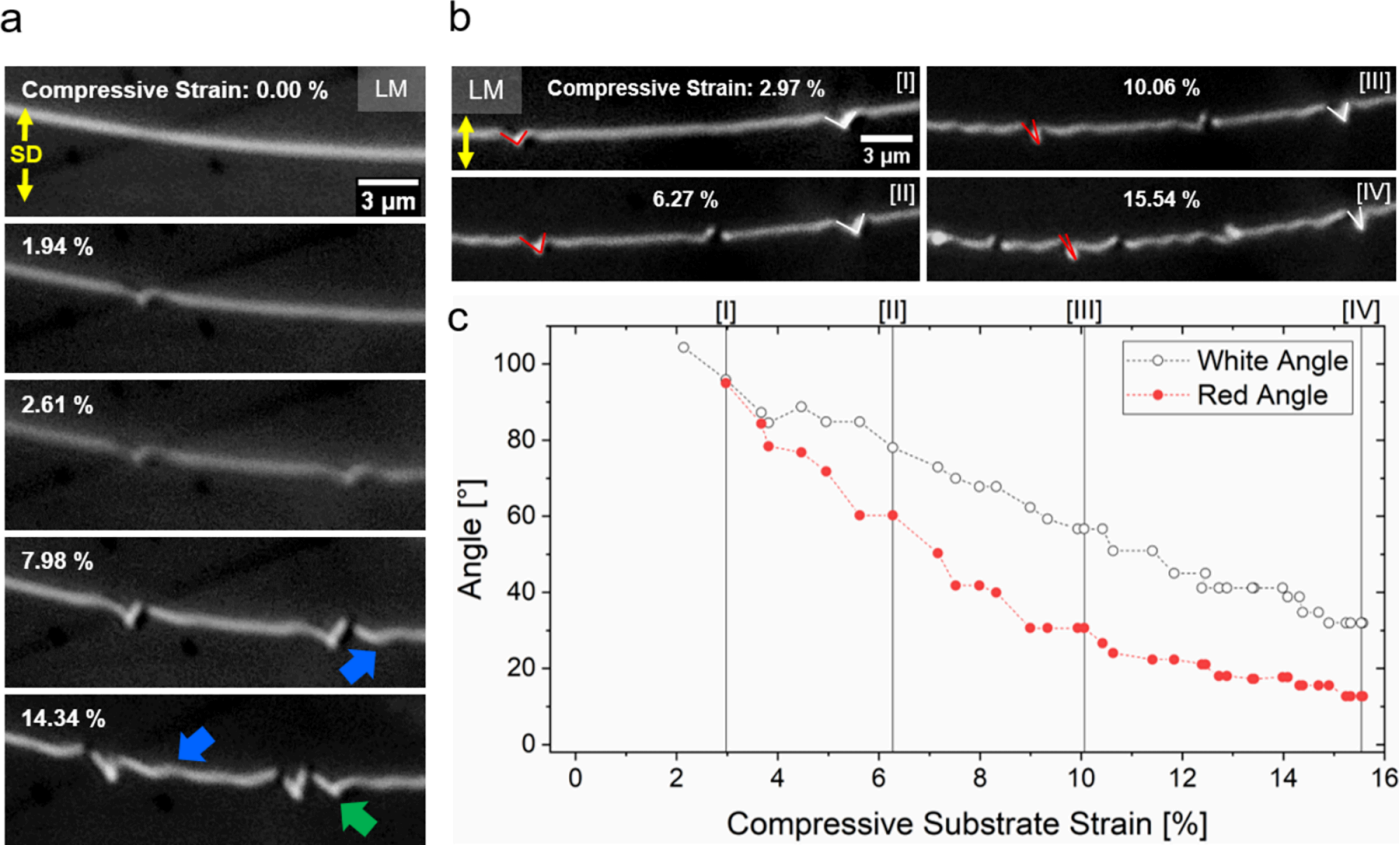
LM still frames from in situ compression tests and evolution of kink angles as a function of loading. The yellow arrows indicate the substrate straining direction (SD). (a) AgNWs on PET showing the formation of nanowire kinks by plastic deformation. Additional deformation sites form with increasing compressive strain, while existing deformation sites become more pronounced. Blue arrows indicate additional undulations corresponding to elastic deformation; green arrow marks an undulation that transformed into a kink. (b,c) LM images of deformation sites on AgNWs, combined with a plot of the corresponding measured kink angles. Angles measured in the images are indicated in red and white. Roman numerals denote the corresponding strain levels in the plot from which the images are taken.

**Figure 5:**
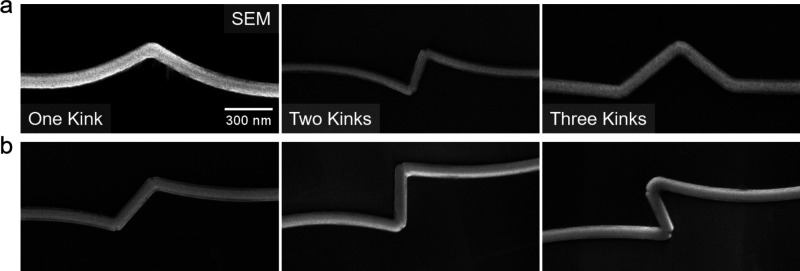
SEM images of compressive deformation sites in different states. (a) Exemplary SEM images of the three different types of deformation sites, defined by the number of kinks. (b) SEM images of deformation sites comprising two kinks, illustrating the different states the deformation site can exhibit. To accommodate increasing compressive strain, the wire progressively folds, and the angles become more acute. Images are taken from separate wires.

Under compressive loading of nanowires on PET, the onset of plastic deformation—i.e., kink formation—occurred at compressive substrate strains between 1.5% and 3.0% (see Table S4 for an overview of all tests conducted on PET). As shown in Figure 4a, with increased straining additional deformation sites appear. Moreover, a steady increase in load leads to a characteristic evolution of the kink structures: already-formed kinks develop progressively more acute kink angles (see Figure 4b). In addition, new kinks form in the immediate vicinity of existing kinks, resulting in double and multiple kink structures, as illustrated by the example in Figure 4a. At higher levels of compressive strain, an undulation of the entire wire (see blue arrows) is observed, which partially relaxes through the formation of new kinks next to existing ones (see green arrow). The characteristic evolution of the kink angle is illustrated by the example shown in Figure 4b. Following the initial kink formation, a folding mechanism emerges to accommodate the increasing strain. This phenomenon can be further evaluated by measuring the kink angle as a function of substrate strain (see Figure 4c). Starting from an angle of approximately 90° at 2.97% compressive substrate strain, the wire locally folds to much more acute angles of around 40° and 20° at 15.54% compressive strain for the two kinks marked in white and red, respectively. It is remarkable that LM, despite its limited resolution, enables not only the early detection of plastic deformation sites under compression (and tension, as shown above), but also allows the evolution of kink geometries to be tracked in situ, with the significant advantage that LM image series can be acquired during straining under realistic ambient or flexibly adjustable conditions. This unique capability can be combined with the strengths of SEM and TEM, which provide much higher resolution for detailed analysis of the kink morphology and internal defect structure.

SEM images of three different deformation states, classified by the number of kinks, are compiled in Figure 5a. These deformation states reflect the situation shown in Figure 4a, where new kinks form close to the initial kink with increasing load (e.g., above 7.98% compressive strain). In this context, an isolated deformation event or deformation site is defined as a kink structure with up to three kinks, separated from other deformation sites by several microns. This definition becomes increasingly complex at high levels of applied strain, as the wire deforms along its entire length. This can be observed in Figure 4b-IV, where the entire wire begins to undulate on the polymer surface, accompanied by a high density of deformation sites. Nevertheless, for compressive loading below 20% as utilized in this study, individual deformation sites are typically distinguishable, and overlap between deformation sites does not pose a problem. Interestingly, kink structures with a single kink are rarely found, which suggests that this configuration is unstable and rapidly evolves into a two-kink structure. This fits to the LM image series shown in Figure 4, where the initial deformation events already appear to exhibit a two-kink structure. The two- and three-kink configurations are much more resilient to further loading due to their ability to accommodate strain through geometric folding. Figure 5b illustrates this process with three SEM images of different deformation sites, each showing two kinks in different states of deformation. Consistent with the LM observations and the evaluation of kink angles in Figure 4, the kink angles become more acute with increasing compensated strain. The combination of these two mechanisms—namely, the formation of kinks at individual deformation sites and their structural evolution through folding—enables AgNWs to withstand high levels of load. Figure 5 further illustrates that, despite the formation of complex kink structures, the wire remains fully interconnected and is thus expected to maintain electrical conductance with low resistivity.

This interpretation is in accordance with our earlier study of nanowire networks, in which we correlated the mechanical deformation behavior and electrical properties of aligned AgNW networks during uniaxial tensile straining of PET substrates.^24^ In that study, we compared two cases in which the nanowires were preferentially aligned either parallel or perpendicular to the straining direction of the substrate. Upon straining, the degradation of electrical conductivity, measured via sheet resistance, was significantly lower when the nanowires are preferentially loaded in compression (i.e., wires preferentially perpendicular to the straining direction) compared to tension (i.e., wires preferentially parallel to the straining direction). In the former case, nanowire kinking was observed in the nanowire network, which allowed the network to retain electrical conductivity, whereas in the latter case, nanowire fracture occurred, leading to rapid electrical degradation. Furthermore, using high-resolution transmission electron microscopy (HRTEM) we found that kink formation is associated with the appearance of complex GBs at the kink positions, consistent with microscopic observations from bending tests of free-standing five-fold-twinned AgNWs in SEM^58^ and TEM.^57^ In our compression tests of single wires on polymer substrate, we expect to observe the same GBs as in the networks with the opportunity to study how the wire structure in the kink region evolves as a function of kink angle and associated nanowire folding. To address this, the wires were transferred to the TEM to provide insights into the local crystal structure at the kink positions.

For the TEM analysis, a transfer workflow employing a sacrificial layer of poly­(3,4-ethylenedioxythiophene):poly­(styrenesulfonate) (PEDOT:PSS)—similar to the transfer process used in organic solar cells—was utilized.^54^ More details about this process can be found in the Methods section. We are aware that this layer can alter the adhesion of the nanowires to the substrate and may affect their deformation behavior. However, no suitable alternative is available, and since the morphology of the nanowire kinks closely resembles that observed by SEM on plain PET, we expect that the internal structure of the kinks is likewise not significantly affected by the sacrificial layer. TEM enables investigation of the local crystal structure of the AgNWs and the formation of crystal defects associated with plastic deformation. Even for perfect (undeformed) five-fold twinned AgNWs , the electron diffraction patterns are rather complex, as they consist of two overlapping zone axis (ZA) patterns—when imaged in the appropriate projection—resulting from the five-fold twinned structure of the wire^14,24^ (see Figure 6a, center). For deformed wires with kinks, the patterns become even more complex (Figure 6a, right), since in addition to the five twin boundaries, one or more new GBs are introduced by plastic deformation. All investigated wires with kinks exhibited the presence of new GBs in the kink region (see Figure 6b). Thus, GBs appear to be characteristic of all kink structures that can be identified by LM and SEM at lower magnification. The TEM images confirm that the newly formed GBs do not compromise the structural integrity of the surrounding wire; consequently, the wire’s conductance is expected to remain high, as confirmed by our earlier study of AgMW networks under mechanical loading, discussed above in connection with the SEM analysis.^24^ Figure 6c, based on scanning transmission electron microscopy (STEM) images, illustrates another phenomenon observed in highly deformed wires: the formation of very small grains at the tips of acute wire kinks. While larger kink angles (left image) introduce only a single new GB into the wire, progressively more acute angles lead to the formation of additional GBs in the kink region (center), eventually resulting in the development of small grains at the kink tip of highly deformed wires (right). The crystalline nature of these grains was verified by dark field TEM analysis (see Figure S6 and related discussion in the Supporting Information). The formation of new grains at kink tips is identified as an additional mechanism that enables already folded AgNWs to accommodate large compressive strains by developing highly acute kink angles without fracturing.

**Figure 6:**
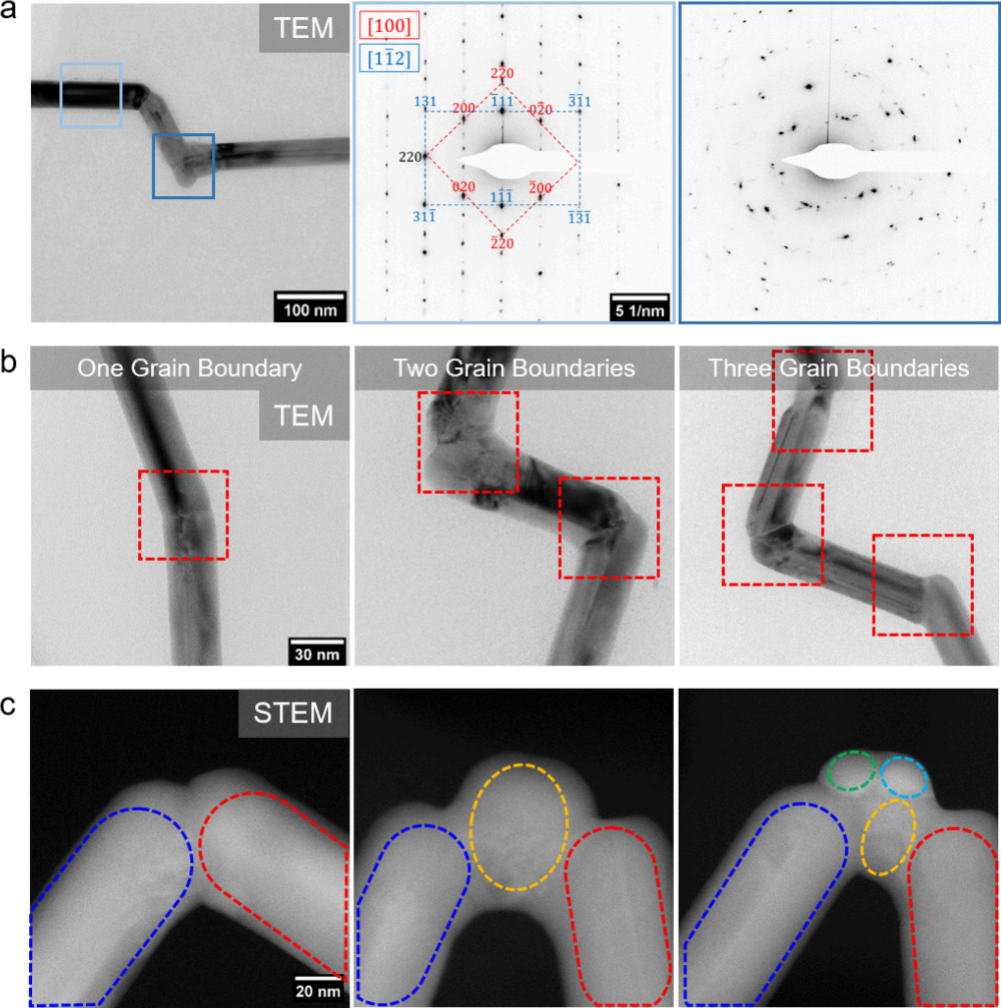
Conventional TEM images, scanning TEM images, and diffraction analysis of plastic deformation sites. (a) TEM image of a deformed wire. Colored boxes indicate the position of the diffraction patterns seen on the right side. Light blue box shows the pattern from the undeformed wire with the overlap of two diffraction patterns resulting from the five-fold twinned wire structure. Dark blue box shows the resulting complex pattern by the introduction of a new GB. (b) TEM images of the three different types of deformation sites showing GBs at the location of the kinks. Red boxes indicate position of new GBs. (c) STEM close-up images of deformed wires illustrating the formation of additional small grains at a higher grade of deformation. Red and blue lines indicate the original wire the other colors indicate new grains formed by deformation.

Regarding the microscopic mechanisms underlying the formation of nanowire kinks and their GBs, we refer to our earlier work,^24^ as well as to relevant experimental and simulation studies in the literature.^29,32,57,58^ Results of MD simulations will be discussed following the presentation of the compressive tests on nanowires supported on PDMS in the next section. These tests exhibit elastic in-plane buckling prior to kink formation, which enables estimation of the critical surface strain at the onset of plastic deformation. From an experimental perspective, performing pure compression tests on thin metallic nanowires is inherently challenging due to the difficulty of achieving perfect alignment of the compressive load with the wire axis. In the few cases where true uniaxial compression has been successfully realized, the test specimens were typically single-crystalline nanowires (or whiskers) with a limited aspect ratio, deliberately chosen to facilitate precise load application and minimize buckling effects. For example, freestanding single crystalline Au nanowires with [110] wire orientation exhibited the onset of plastic deformation through the nucleation of prismatic dislocation loops, which subsequently slipped down the wire. At higher degrees of deformation common dislocation slip on inclined {111} slip planes sets in resulting in slip steps on the wire surface.^55,56^ For five-fold twinned AgNWs, as employed in transparent electrodes, the situation is markedly different. These nanowires exhibit a much higher aspect ratio that precludes pure compression testing, particularly in freestanding configurations. Instead, such tests transition into bending, as the nanowire undergoes lateral deflection and elastic bending prior to the onset of plastic deformation.^29^ In the present work, the nanowires are attached to PET or, as discussed below, to PDMS. The combination of the low mechanical compliance of PET and the strong adhesion of the nanowires keeps the wires largely straight until kink formation occurs (see Figure 4a). We assume that the instability of the straight nanowire under increasing compressive loading eventually leads to local elastic buckling, followed by abrupt kink formation. However, the temporal and spatial resolution of our LM studies is insufficient to resolve the microscopic details of this process. As will be shown below, the behavior differs for nanowires attached to PDMS. The significantly higher mechanical compliance of the elastomer, combined with weaker adhesion, permits periodic elastic in-plane buckling of the nanowires prior to kink formation. Nonetheless, the kink formation remains as a sudden event, at least within the temporal resolution of our experiments.

Together with the insights gained from the tests in the LM and SEM, this provides a comprehensive overview of the behavior of five-fold twinned AgNWs on PET and their deformation behavior under mechanical straining of the PET substrate. Under compression, the wires employ a combination of kink formation and folding to accommodate significant substrate strain. At very high degrees of wire deformation, additional small grains are formed at the very tip of the wire kinks. This combination of mechanisms allows the wires to remain structurally intact over a wide range of compressive substrate strains.

### Compressive Loading on PDMS

Similar to PET, PDMS exhibits lateral contraction under tensile strain, as governed by its Poisson’s ratio. The Poisson’s ratio of PDMS, like that of most polymers, is approximately 0.5 (see Figure S1b). Figure 7 shows the deformation of a long nanowire (initial length: 69.5 μm) on PDMS, subjected to compressive loading resulting from its orientation perpendicular to the tensile straining axis. As evident from the LM image series, the wire exhibits markedly different deformation behavior compared to its counterparts on PET. In contrast to PET, the wire does not immediately form sharp kinks; instead, it initially undergoes smooth, sinusoidal deformation, characteristic of an elastic response. The amplitude of the sinusoidal deformation increases until the transition from elastic to plastic deformation occurs at the point of maximum amplitude, where smooth bending gives way to localized kink formation. (see Figure 7a). For the three periods with the highest amplitude, this transition occurs within a narrow strain range of 7.49–7.88%. Further increasing the compressive substrate strain induces plastic deformation in additional existing periods, resulting in a total of six kinks across the original wave structure at a compressive substrate strain of 17.89%.

**Figure 7:**
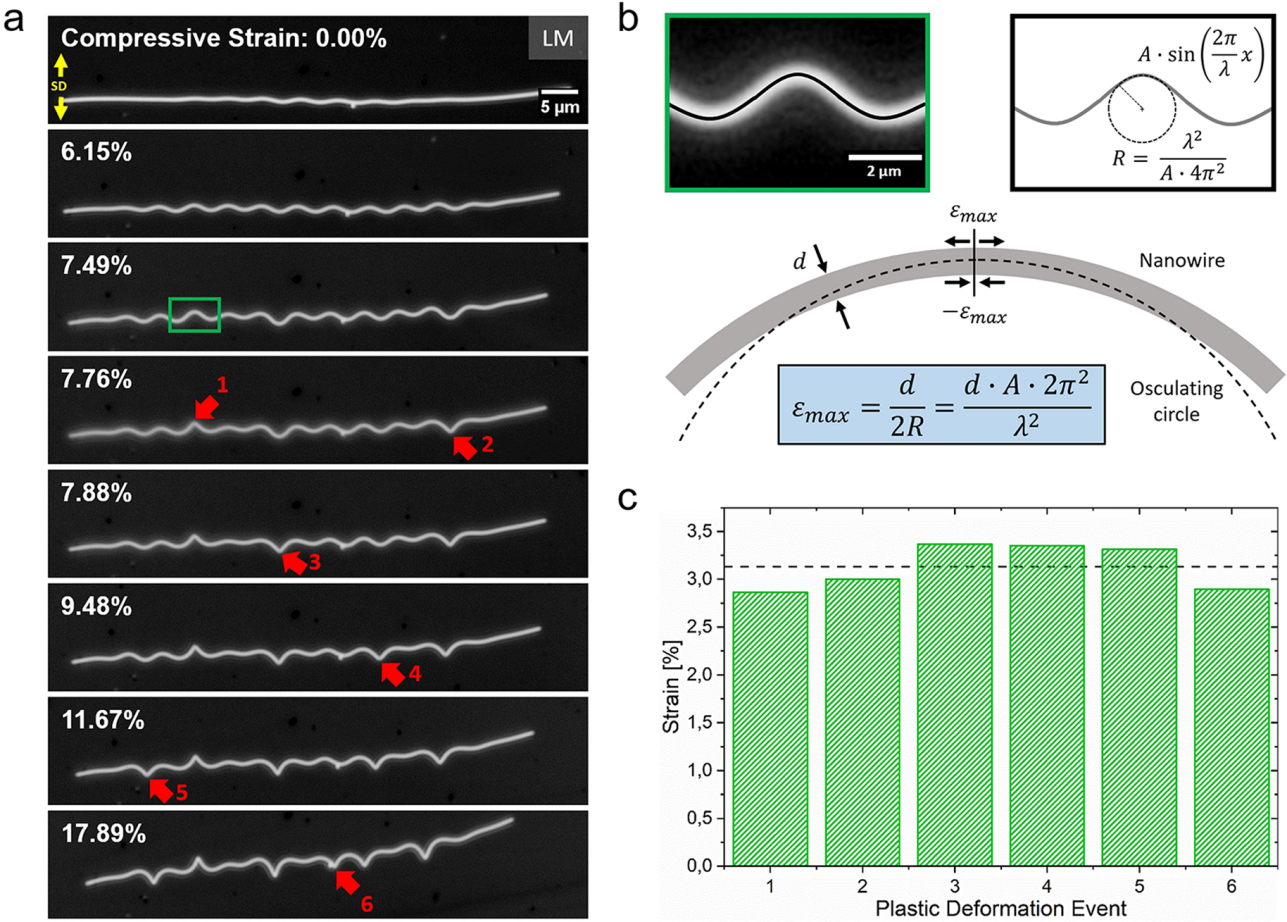
AgNW on PDMS subjected to compressive loading, showing local transition from elastic to plastic deformation. The yellow arrows indicate the substrate straining direction (SD). (a) LM images of a long wire at different levels of compressive straining of the PDMS substrate. With increasing stain, the wire develops periodic undulations with growing amplitude. At a critical wire bending, the elastic deformation changes to plastic deformation, as revealed by the formation of kinks. Six such transitions from elastic to plastic are numbered and marked by red arrows. (b) Enlarged section of the LM image, taken at 7.49% compressive substrate strain, shortly before the kink forms at the maximum amplitude of the undulation shown. The wire shape before kink formation can be approximated by a sinusoidal function with amplitude *A* and wavelength λ, as shown by the superimposed curve. The dark line represents not only the shape of the wire but also its thickness *d* ≈ 60 nm, which is much smaller than the apparent thickness of the wire in the LM image. The strongest wire bending occurs at the extrema of the sinusoidal curve. At this point, the osculating circle has a minimum radius of curvature *R*, given by the formula in the inset. Using the real diameter *d* of the wire, the maximum strain ε_max_ at the outer and inner wire surfaces can be calculated, using the formula in the box (see SI for details). (c) Critical strain values ε_max_ at outer (convex) wire surface measured shortly before the transition from elastic to plastic deformation for the six deformation events numbered in figure part (a).

From Figure 7a, it can be seen that the sinusoidal deformation of the nanowire does not extend all the way to the wire ends, but is suppressed within a certain distance from both ends, leaving these segments straight. This behavior can be attributed to strain relaxation by slippage of the wire on the PDMS substrate, as previously discussed in the context of the tensile experiments. The weaker adhesion of the wire to the PDMS substrate (compared to PET) makes this relaxation particularly effective at the wire ends which can retract freely. Furthermore, the reduced adhesion also facilitates the lateral sliding of the nanowire required for the in-plane sinusoidal deformation in its central region. However, the weak adhesion does not generally lead to detachment of the nanowire from the substrate, which would be required for relaxation via out-of-plane buckling. Such out-of-plane buckling was observed in only a single compression experiment, where periodic contrast features appeared in the LM images. The corresponding image series is shown in Figure S10 of the Supporting Information. The appearance of periodic dark contrast at 3.1% compressive strain, combined with a corresponding reduction in total wire length and the overall straight shape of the wire (in this projection), can only be interpreted as out-of-plane buckling. Between 3.1% and 5.8% strain, the contrast transitions to the typical appearance observed in all other experiments: a uniformly white wire with more or less sinusoidal in-plane undulations. Evidently, the initial out-of-plane buckling evolved into conventional in-plane buckling, restoring full contact of the nanowire with the substrate along its entire length.

We now take a closer look at the transition from elastic to plastic deformation, as revealed by kink formation in Figure 7a (red arrows). As expected, the formation of kinks is observed where the wire shows the strongest bending or the smallest radius of curvature, which is the case in those regions, where the sinusoidal modulation has its maximum amplitude. Bending tests on five-fold twinned AgNWs^29^ and corresponding atomistic simulations^24,37^ have shown that the first crystal defects initiating plastic deformation form on the outer convex surface of the wire, where the tensile strain reaches its maximum. It is therefore of interest to estimate the critical tensile strain at the wire surface corresponding to the onset of plastic deformation from the experimental images. For this, Figure 7b shows an enlarged section of the wire from Figure 7a at a compressive substrate strain of 7.49%, just before kink formation in this region. The wire has an almost sinusoidal shape, as shown by the superimposed sinusoidal function. As can be seen in Figure[Fig fig7]a, at the next higher strain level of 7.76%, a kink appears at the point of greatest amplitude (red arrow numbered “1”), so that the transition between elastic and plastic deformation must have occurred between 7.49% and 7.76% compressive substrate strain for this particular wire segment. To estimate the critical tensile strain at the wire surface, we approximate the wire shape in Figure[Fig fig7]b by a sinusoidal curve *f*(*x*) = *A*sin­(2π*x*/λ) with period λ and amplitude *A*. The local radius of curvature *R* can be calculated for each point on the sinusoidal curve, as outlined in the Supporting Information (see Figure S7). *R* has its minimum value *R*_min_ = λ^2^/(*A*4π^2^) at the positions of the extrema of the sinusoidal function, indicating the strongest wire bending at these points. The corresponding osculating circle is depicted in Figure[Fig fig7]b on the top right schematic and the enlarged view of the maximum below. In order to calculate the tensile strain at the outer wire surface, the precise diameter *d* of the wire must be known. Since Figure 7b is an optical micrograph, the nanowire appears significantly wider than it actually is. We know from TEM investigations that the actual diameter of the wires used in these tests is *d* = 57.39 ± 7.06 nm (see Figure S7 for a statistical evaluation). With this knowledge and considering that the wire diameter is much smaller than the radius of curvature, the tensile strain on the outer wire surface can be calculated using the formula ε_max_ = *d* *A*2π^2^/λ^2^, also depicted in Figure 7b. By inserting the real wire diameter (*d*) and the values for λ = 4730 nm and *A* = 566 nm, as measured from Figure 7b, the following results are obtained: a minimum radius of curvature *R*_min_ = 1002 nm and a tensile strain on the wire surface ε_max_ = 2.86%. As the wire section shown in Figure 7b was recorded shortly before the onset of plastic deformation (see red arrow in Figure 7a), ε_max_ can be directly interpreted as the critical tensile strain at the wire surface, at which plastic deformation sets in. The magnitude of strain is the same at the inner (concave) surface of the wire where compressive strain (ε < 0) is observed instead of tensile strain. However, it has been demonstrated that the wire is less susceptible to plastic deformation on the compressive side of the wire than on the tensile side.^24,37^ This is the reason why we only consider the tensile surface strain here.

The described evaluation was carried out for all six deformation events, which are marked in Figure 7a (red arrows and numbering). In each case, the waveform shortly before plastic deformation was analyzed. The magnified sections of the corresponding optical microscope image together with the extracted values for λ and *A* are shown in the Supporting Information (see Figure S9 and Table S1). Figure 7c shows the maximum tensile strain values on the wire surfaces at the transition from elastic to plastic deformation. Interestingly, all values cluster within a tensile surface strain range of 2.9–3.5%, comparable to that for nanowire fracture in tension, suggesting that the onset of plastic deformation is initiated by a well-defined and largely reproducible microscopic process.

In total, six nanowires were tested in compression on PDMS substrates, all exhibiting the same characteristic behavior: sinusoidal elastic buckling, followed by a transition to plastic deformation through kink formation at a critical substrate strain. The LM image series of four of these tests are shown in Figures S28–S31, while another test is presented in Figure 8 as part of a cyclic loading experiment (see next section). Table S5 summarizes the relevant data extracted from all six compression tests, including the one discussed in detail above (see Figure 7). As can be seen from the table and the corresponding LM image series, two of the wires are relatively short (<20 μm), which causes their deformation behavior to be dominated by strain relaxation at the wire ends. Nevertheless, both wires exhibit periodic in-plane buckling in the central region, which transitions into the formation of a single kink. Since the estimation of the critical tensile strain at the convex wire surface relies only on the local curvature of the wire, the same evaluation procedure as used for Figure 7 can be applied. Altogether, the critical tensile strain at the convex outer surface of the nanowires immediately prior to kink formation was evaluated for 19 kinks across the six tested wires. The values range from 2.7% to 4.12%, with a mean critical strain of 3.25 ± 0.42%. As noted earlier, the evaluation for all six wires was based on the assumption of a mean wire diameter of *d* = 57.4 nm, since the actual wire diameter could not be determined from the LM images. However, this assumption alone cannot account for the observed data spread. Since plastic deformation requires the nucleation of defects, we attribute the variability primarily to the stochastic nature of these nucleation events.

**Figure 8:**
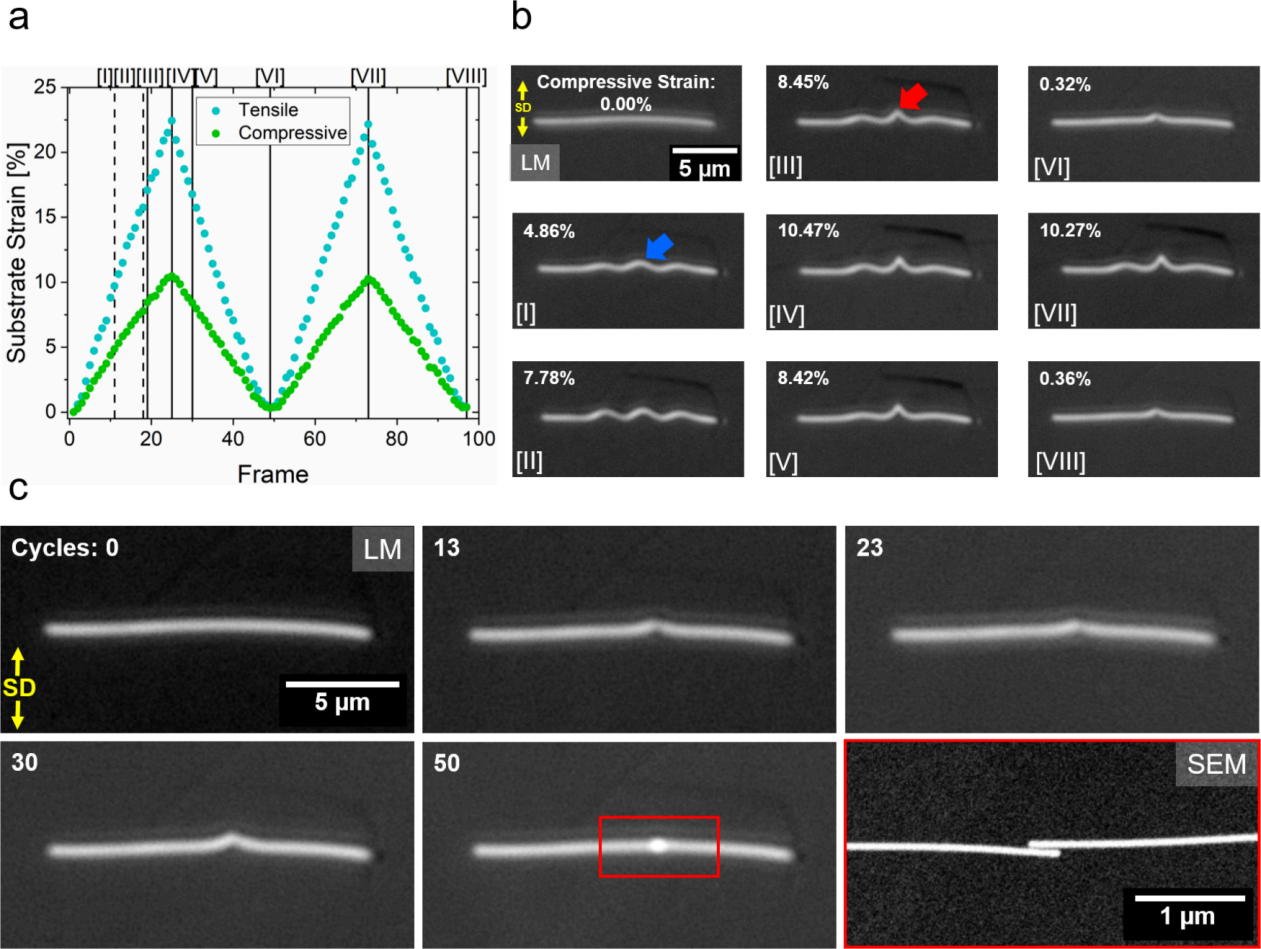
Elastic and plastic deformation of an AgNW on PDMS during cyclic compressive loading, investigated by in situ LM and ex situ SEM. The tensile straining direction (SD) of the substrate is indicated by yellow arrows and is oriented perpendicular to the nanowire. As a result, the nanowire experiences compressive loading along its axis. (a) Substrate strain measured in both the tensile (blue) and compressive (green) directions during the first two cycles. (b) LM images captured during the in situ experiment, showing the onset and increase of elastic deformation (I, marked by a blue arrow) and the subsequent transition to plastic deformation (III, marked by a red arrow). The plastic deformation becomes increasingly pronounced with greater compressive strain and remains visible after strain release. Roman numerals in the LM images correspond to the respective strain states indicated in panel (a). (c) LM images of the nanowire taken after various numbers of compressive loading cycles. Notably, no significant morphological changes are observed until after the 50th loading cycle, at which point a distinct contrast change emerges at the deformation site. The accompanying SEM image reveals a fatigue-induced fracture at the same location, coinciding with the original kink formed during early plastic deformation.

The local transition of sinusoidal bends into kinks exhibits strong similarities to the deformation behavior observed in bending tests of freestanding nanowires. As discussed above, Vlassov et al. reported a similarly abrupt transition from elastic to plastic deformation during in situ bending tests in a SEM setup.^58^ Kim et al. performed cyclic in situ bending tests on five-fold AgNWs, which also exhibited a transition from elastic to plastic deformation at a radius of curvature corresponding to a surface strain of 3.64%, a value closely matching our findings.^29^

MD simulations of bending tests give an insight into the defect processes involved in the transition from elastic to plastic deformation. In this context, we refer to MD simulations of nanowire bending conducted as part of our earlier study on the mechanical and electrical properties of flexible AgNW network electrodes.^24^ It should be noted that the nanowires used in those simulations had a diameter of only 15.2 nm, and were therefore significantly thinner than the nanowires investigated in the present work, which have a mean diameter of 57.4 nm. In the case of the simulated bending test, plastic deformation is initiated by the nucleation of partial dislocations from the tensile side of the nanowire, followed by the nucleation of full dislocations from the compressive side (ref 24, see Figure 8 and Movie 4). On the compressive side of the nanowire, dislocations on different slip planes interact and form dislocation locks containing sessile dislocations. The interlocked dislocation networks act as a barrier to the glide of further dislocations, and these dislocations accumulate in the incipient GB region. With increasing bending, this accumulation ultimately gives rise to a tilt GB, in agreement with HRTEM observations. An additional example of such a tilt GB, with a tilt angle of 35°, is shown in Figure S32. Notably, although the GB appears highly localized, it exhibits a surface reconstruction in the form of a triangular defect. This structural feature is proposed to enhance the mechanical stability of the kink and may serve to prevent premature failure, as discussed in ref 24.

### Cyclic Loading on PDMS

As mentioned earlier, the use of PDMS as a substrate enables cyclic loading and thereby facilitates the investigation of fatigue behavior. Figure 8 shows the results of a cyclic in situ straining test with two loading cycles. Compared to the previous wire, here a relaxation step is introduced after a certain amount of load. This process is then repeated a second time resulting in the strain curve shown in Figure 8a. The transition from elastic to plastic occurs between 7.78% and 8.45% of compressive substrate strain. Due to the shorter length of the wire only one kink is formed. Once formed, the kink remains and becomes more pronounced as the strain increases. As the load is reduced, the kink becomes less pronounced, but remains present even after all applied load has been removed from the substrate (see Figure 8b-VI). During the second loading cycle the plastic deformation site changes back to its more pronounced appearance including a rise of smaller undulations beside the plastic kink (Figure 8b-VII). As described for PET, the formation of visible kinks in the LM correlates with the generation of new GBs at the kink position. In the case of PDMS, a pronounced undulation of the wire can be seen before and also after kink formation. Thus, in contrast to PET, pronounced elastic and plastic deformation of the wires can be observed on PDMS. Here, the visibility of the preceding elastic deformation can be attributed to the lower surface interaction of the AgNW on PDMS. The lower adhesion already seen in the tensile tests gives the wire more freedom to slide on the substrate surface (see Figure 3). During compression, the wire must be able to slide sideways in order to form the sinusoidal undulation that is observed. The abrupt transition from elastic to plastic deformation has been reported in in situ bending tests of five-fold twinned AgNWs, where the deformation localizes at a certain bending angle and then abruptly forms the new GB to relax strain energy.^57,58^ Although the stress applied in bending tests differs from our compression test, these results are very much in line with our findings.

By continuing the cyclic testing of the wire seen in Figure 8b the investigation of the fatigue behavior is possible (see Figure 8c). For that the wire is cyclically loaded in compression up to a strain value of ∼10.5%. The test was interrupted after different numbers of cycles indicated in the LM images. The images were always taken after complete removal of the substrate strain. As previously seen, the newly formed GB remains visibly as a small buckle on the wire. This appearance changes after the 50th cycle significantly. Here the kink transforms into a bright spot visible in the LM. Complementary imaging in the SEM reveals that the wire has fractured precisely at the location of the GB (see Figure 8c, last image). This fatigue fracture most likely was caused by an accumulation of defects due to the repeated loading and deformation of the AgNW.

Cyclic testing methods are widely used in the field of flexible electronics since they offer insight into the possible lifetime of the device. Especially for flexible transparent electrodes cyclic bending test are widely adopted.^62,63^ Nanowire network electrodes have shown a high resilience to cyclic loading.^29,64^ For single wires cycle fatigue test are usually performed in a tensile setup.^28,65^ In the case of five-fold twinned AgNWs such tests revealed a self-healing property, meaning that incipient plastic deformation during tensile straining partially recovered upon unloading of the wire. Using in situ TEM this phenomenon of plastic recovery could be attributed to reversible dislocation activity in the five-fold twinned microstructure of the wire.^35^ In a subsequent loading cycle dislocation nucleation occurred at the same position. In contrast to cyclic tests in tensile mode, cyclic compression tests are not commonly performed for AgNWs. During our cyclic compression tests we do not see a recovery of plastic deformation. Rather, plastic deformation manifests itself in the formation of a GB which persists during subsequent loading cycles. However, reversible dislocation activity cannot be ruled out since only in situ LM was applied which has insufficient resolution to detect individual defects.

In contrast to the intentional plastic deformation, which led to fatigue fracture as reported above, we also performed cyclic straining of wires purely in the elastic regime (see Figure 9). Cyclic tests in the elastic regime are not yet common for AgNWs. Even the usual bending tests result in deformation or breakage of the wires.^9,22,66^ In this cyclic test we limited the maximum load precisely at the threshold of plastic deformation. This was achieved by slowly increasing the load in the first cycle until the first plastic deformation event occurred in the form of a kink at 5.12% compressive substrate strain, as indicated by the red arrow in Figure 9. In subsequent straining cycles the sample was then again loaded to the same maximum strain value, and LM images were taken at maximum load after various numbers of cycles. A total number of 1000 straining cycles were performed during this test which was made possible by the use of a specially designed straining stage which is described in more detail in the Methods sections. This programmable stage enables a precise, repeatable straining by a stepper motor. At all recorded straining steps the wire shows uniform undulations with an approximately sinusoidal shape. The last image in Figure 9 shows the wire after completion of 1000 cycles, where it is completely intact in contrast to the fatigue fracture of the wire in Figure 8c. This confirms that the elastic deformation is completely reversible. In addition, the initial site of plastic deformation still appears to be intact after 1000 cycles. One probable reason for this is the relatively low load on the deformation site, as in each cycle the wire was only strained to the point where the GB was formed in the first place. Obviously, under these conditions the accumulated defects where not sufficient to lead to a wire fracture.

**Figure 9:**
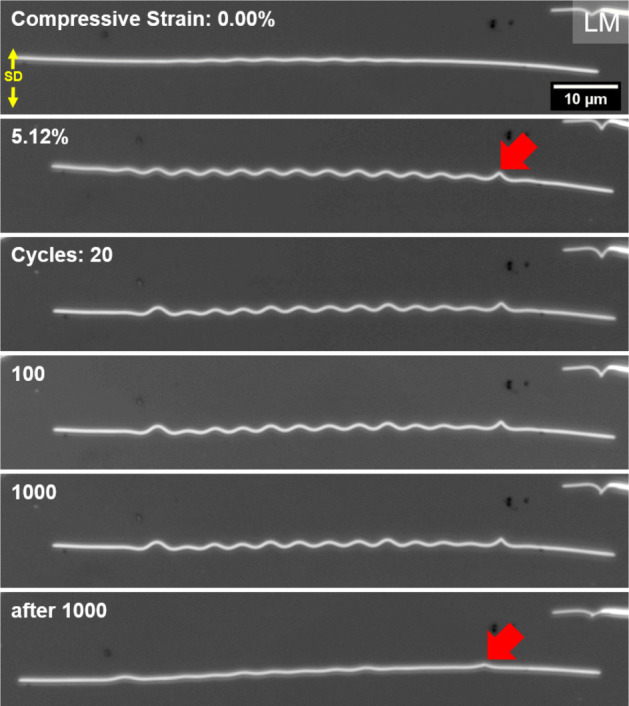
LM images of a cyclic compression test performed on a long AgNW. The first two images show the wire in its initial state and in its strained state at the maximum strain. The red arrow indicates the only plastic deformation of the wire. The rest of the wire shows a sinusoidal elastic deformation. The next three images show the wire at different numbers of straining cycles, each image taken at the maximum strain level. The last image shows the wire after 1000 cycles, after the strain has been removed. The red arrow indicates the plastic deformation spot introduced in the first cycle. The yellow arrows indicate the substrate straining direction (SD).

Although the investigation of the deformation modes of individual nanowires, including nanowire fracture in tension and kink formation in compression, is in the realm of fundamental research, the basic principles are likely to be of great importance for applications. The straining setup with wires placed on a substrate utilizes a very basic phenomenon, namely the lateral constriction of the polymer according to the Poisson’s ratio. In applications, the wires are usually not applied directly to a polymer substrate but are part of a device comprising different layers. The adhesion of the wires and thus the load transmission strongly depend on the underlying layer, which can be a transport layer in the case of a flexible solar cell, for example. The situation is complicated further if the wires are buried under the surface. However, the testing setup and the microscopic techniques utilized in this study are quite flexible and can be adopted to more complex situations thus enabling investigation of more realistic scenarios for device applications.

While the present work focused on the mechanical response of isolated nanowires, dense nanowire networks are required for use as flexible transparent electrodes. Interestingly, the same deformation modes observed in isolated wires, namely fracture in tension and kink formation in compression, are also observed in dense AgNW networks, as reported in our earlier work.^24^ Furthermore, the orientation of the wires relative to the tensile loading axis, which plays a key role in the mechanical response of isolated wires, also has a strong influence on the mechanical and electrical response of wire networks. A preferential orientation of the wires parallel to the loading axis results in wire fracture, associated with a drastic increase in sheet resistance. In contrast, a preferential orientation of the wires perpendicular to the loading axis, leads to kink formation and folding, which maintains a low sheet resistance up to considerable strain values. A difference is that, in the case of networks, wire junctions represent further constrictions for elastic and plastic deformation of the wires. This leads to additional deformation modes for wires in networks that are not observed in isolated nanowires.^24^ Therefore, in a next step, we want to use our setup to investigate the mechanical properties of two crossing nanowires and the role of the junction for the elastic and plastic deformation of the wires. In this context, the effect of nanowire welding, which is usually applied to reduce the resistance of the wire junctions, on the mechanical and electrical response to strain is of particular interest.

## Conclusions

In summary, a detailed correlative microscopy study of the deformation and fracture behavior of five-fold twinned AgNWs applied to two different polymer substrates, PET and PDMS, was conducted. For this purpose, a combination of in situ tensile straining tests and correlative LM/EM investigations was employed. By exploiting the lateral constriction during tensile straining, the polymer enabled both tensile and compressive testing of AgNWs, depending on their orientation with respect to the loading axis. The findings of the present work can be summarized as follows:

- During tensile testing the wires exhibit different behaviors depending on the polymer. On PDMS, even longer wires typically undergo only a single fracture—occurring at around 3% applied substrate strain—with the resulting gap widening as the load further increases.
- On PET the wires also fractures at around 3% substrate strain, but multiple fracture events occur almost simultaneously within a small strain window (<1%) leading to fragmentation of the wire into segments of approximately equal length. This different behavior can be attributed to stronger adhesion of the wires to PET compared to PDMS.
- Compressive tests of wires on PET revealed localized kink formation along the wires enabling accommodation of strain by a folding mechanism. SEM investigations show that up to three kinks are involved in this process, with the kink angles becoming increasingly acute as the applied substrate strain increases.
- Investigations in the TEM showed that the kink formation observed in LM and SEM is associated with the formation of a new GB crossing the wire at the position of the kink. Furthermore, at high degrees of deformation, i.e., at acute kink angles, nm sized grains are formed at the tip of the kink.
- Wires that were tested under compression on PDMS showed a different deformation behavior than their counterparts on PET. On PDMS, the wire shows a pronounced elastic deformation in the form of a sinusoidal curve before forming a kink and thus a new GB by plastic deformation. This transition from elastic to plastic deformation occurs at the maxima of the sinusoidal deformation, when the tensile strain on the convex wire surface reaches a value around 3%.
- PDMS enables cyclic testing of the wires in compression mode. In these fatigue tests, GBs formed in the kink regions act as potential fracture sites and can ultimately lead to wire failure under repeated loading. In contrast, the sinusoidal elastic deformation exhibited full reversibility, even after 1000 test cycles.

From a methodological point of view, the present work demonstrates that LM can be used effectively to gain deep insights into the elastic and plastic deformation behavior of individual nanowires with diameters in the range of 50 nm. If the investigated nanowires are separated from each other and show sufficient reflection or scattering contrast, they can be localized with a precision that is not limited by the optical resolution. This enabled the sinusoidal elastic deformation of the nanowires under compressive loading to be visualized with high sensitivity, as well as the formation of kinks during the transition to plastic deformation. Subsequent correlative analyses using electron microscopic methods such as SEM and TEM can then be used to reveal further microscopic details of the plastic deformation down to nanometer and atomic scale and validate or interpret the light microscopic observations. A significant advantage of LM in the investigation of nanowires on polymer substrates is the flexible choice of the sample environment (atmosphere, pressure). This allows the conditions to be adapted to those that are most realistic. In contrast, EM methods have significant limitations in this respect. They are often carried out under artificial conditions (e.g., in vacuum) and may even change the sample under investigation. The interaction of the electrons with the sample can lead to a change in the mechanical properties of the polymer with unknown consequences for tensile, compressive or cyclic tests. Furthermore, the application of a thin conductive layer is often employed to prevent charging of the polymer during SEM investigations. In both cases the microscopic examination directly affects the sample system in a way that is difficult to assess. The strength of the approach pursued here lies in the correlation of LM and EM, which combines the advantages of both methodologies: the flexible design of experimental conditions in LM and the much higher resolution of EM.

Our findings suggest that enhancing the mechanical resilience of devices involves not only aligning the nanowires according to anticipated loading scenarios but also modifying the adhesion of the electrodes to the underlying layers. As demonstrated in our experiments, a certain degree of freedom allows the nanowires to deform elastically, which can benefit overall device performance under cyclic loading conditions. However, considering that AgNWs are commonly applied as networks, further investigations into the influence of other wires on this behavior are needed. In particular, attention should be given to the impact of welding points between the wires, as these could potentially limit their capacity for elastic deformation.

## Methods

### Sample Preparation

As substrates for the straining test, two different materials were used. For the tensile straining test, a PET foil (Goodfellow GmbH) with a thickness of 23 μm was employed. The cyclic and fatigue tests were performed using PDMS (Dow Corning, Sylgard 184). The PDMS was prepared by mixing the base with the curing agent in a 10:1 ratio, followed by degassing in vacuum for 20 min to remove air bubbles. The mixture was then poured onto microscope slides (75 × 25 mm or 75 × 50 mm) and cured at 95 °C for 3 h, forming a ∼1 mm thick layer. The PDMS layer was also used as an adhesive to fix the 23 μm PET foil onto the microscope slides.

All straining samples were prepared with a doctor blade (Zehntner GmbH) under ambient conditions. For the light microscope studies, nanowires with a nominal diameter of 100 nm (ACS materials) were used. As shown in Figure S8, the actual wire diameter was ∼57 nm, as determined by STEM analysis. The dispersion had a concentration of 20 mg/mL in water. TEM investigations of nanowire kinks were performed on wires with a nominal diameter of 85 nm (ECOS X3), dispersed in 2-propanol at a concentration of 20 mg/mL. For isolated wires, the 100 nm nanowire dispersion was further diluted to 0.2 mg/mL and the 85 nm nanowire dispersion to 0.4 mg/mL. For both wire types, a blade-substrate gap of 400 μm and a table temperature of 40 °C were used. Depending on the microscope slide size, either 75 or 150 μL of dispersion was applied. Coating speeds were 20 mm/s for the 100 nm AgNWs and 3 mm/s for the 85 nm AgNWs. These parameters produced a preferential orientation of the wires along the coating direction, enabling alignment-specific straining tests. The 100 nm wires were subsequently annealed at 100 °C for 3 min, and the 85 nm wires at 100 °C for 4 min.

For SEM investigations, the strained wires on the substrate where coated with carbon to ensure good imaging conditions.

For the TEM studies, a layer of PEDOT:PSS (Al 4083, Heraeus) was applied onto the polymer substrates. The PEDOT:PSS was diluted with 2-propanol in a 1:1 ratio. Additionally, the surfactant Capstone FS-31 (DuPont) was added at a concentration of 0.1% relative to the PEDOT:PSS content to further improve the wetting properties. The layer was fabricated by doctor blading, using a 400 μm gap, as for the nanowire coating. The table was heated to 50 °C, and the coating was performed at 40 mm/s. An annealing step at 140 °C for 10 min followed. Depending on the layer quality, a second coating step was applied using the same parameters as the first.

### In Situ Straining Tests under the Light Microscope

During this study, two different custom-built straining stages were used. Both can be seen in Figure S2. The manual stage is entirely made of aluminum, with screws made of brass. This material choice enables the use of the stages in both the SEM and the light microscope. In the SEM, these materials are necessary for operation in immersion mode, which applies a magnetic field to achieve a higher resolution. To prepare a sample for straining, a 20 mm-wide stripe of the wire-polymer-composite was cut with a razor blade. The stripe was fixed between two clamps, each equipped with two screws. Displacement of the clamps was achieved by rotating a fine-thread screw (M6 × 0.5). The applied strain could be adjusted by changing the distance between the clamps. In this study, a distance of 16.67 mm between the clamps was used, resulting in 3% strain per full rotation of the screw. This setting was typically used as a reference point for straining, as the exact strain on the sample was determined from the images through image correlation.

The second custom-built stage is automated with the help of an electric stepper motor. This stage is also constructed entirely from aluminum and brass, allowing its use in the SEM. The setup is similar to the manual stage, with two clamps holding the cut substrate. The holder is displaced via the stepped motor, and the force is transmitted via a set of gears. The amount of applied strain is controlled by the number of steps taken by the motor and, as with the manual stage, can be adjusted changing the distance between the clamps. The straining process is operated via a controller unit and associated software, which regulates the number of motor steps. The software can execute programed sequences, which was used to perform cyclic testing with more than just a view straining cycles.

The used light microscope in this study was a Nikon Eclipse LV 100 ND, equipped with four extra-long working distance objectives. 10× magnification with a numerical aperture (NA) of 0.30, 20× magnification with NA 0.40, 50× with NA 0.60, and 100× with NA 0.60. Most of the microscopy images in this study were acquired using the 50× or 100× objectives, as their higher NA provides improved resolution of the individual wires. Extra-long working distance objectives were necessary to allow placement of the straining stage beneath the microscope.

### Tracking-Base Alignment and Strain Calculation

During the in situ straining tests, images from the same position on the sample were taken. However, alignment of the field of view was subject to human error, making realignment of the images necessary. This was accomplished using a Python script than implements subpixel feature tracking. The script tracks an arbitrarily chosen feature across the complete image stack by calculating a cross-correlation function, which scans the feature over each image and computes a correlation coefficient based on a comparison criterion, generating a correlation map. Each point in this map is assigned a floating-point value indicating how well the position matches the feature. This enables calculation of the shift between the feature in the first image and that in the current image, allowing for image alignment. This process is initially limited to pixel accuracy, as only one pixel corresponds the the highest correlation value. To achieve subpixel precision, the area surrounding this pixel was fitted with a two-dimensional Gaussian function, which accounts for feature shifts smaller than the pixel size.

Furthermore, to obtain strain values from the image stack, a similar tracking algorithm was applied. In this case, the same suppixel feature tracking was used, but on two features in an already aligned images stack. Determining the exact position of the features in each image allows also allows calculation of the distance between them and its evolution throughout the complete image stack. This makes it possible to determine the displacement of the two features during straining and, consequently, to calculate the strain form the images. Additional information on the feature tracking is provided in the SI.

## Supplementary Material















## Data Availability

Raw data supporting this study are available at Zenodo: https://doi.org/10.5281/zenodo.15640783.

## Notes

Poisson’s ratio analysis for PET and PDMS, images of used straining stages, full LM images of tensile and compressive tests from wires on PET, additional analysis of strain data from tests of PET and PDMS, dark field TEM analysis of small grains on highly deformed wires, in depth example of elastic deformation analysis and used images, wire diameter analysis via STEM images (PDF)
Movie 1: In situ tensile straining test of AgNW on PDMS (AVI)
Movie 2: In situ tensile straining test of AgNW on PET (AVI)
Movie 3: In situ compressive straining test of AgNW on PET (AVI)
Movie 4: In situ compressive straining test of AgNW on PET (AVI)
Movie 5: Cyclic in situ tensile straining test of AgNW on PDMS (AVI)
Movie 6: Cyclic in situ tensile straining test of AgNW on PDMS (AVI)

## †

Max Planck Institute for Sustainable Materials, Max-Planck-Straße 1, 40237 Düsseldorf, Germany

## ‡

Empa, Swiss Federal Laboratories for Materials Science and Technology, Laboratory for Mechanics of Materials & Nanostructures, Feuerwerkerstrasse 39, 3602 Thun, Switzerland.

## Abbreviations

Ag: silver
AgNW: silver nanowire
Au: gold
CNT: carbon nanotube
Cr: chromium
Cu: copper
EM: electron microscopy
GB: grain boundary
ITO: indium tin oxide
HRTEM: high-resolution transmission electron microscopy
LM: light microscopy
MD: molecular dynamics
PDMS: polydimethylsiloxane
PEDOT:PSS: poly­(3,4-ethylenedioxythiophene) polystyrene sulfonate
PET: polyethylene terephthalate
SEM: scanning electron microscopy
STEM: scanning transmission electron microscopy
TCE: transparent conductive electrodes
TEM: transmission electron microscopy
Ti: titanium
ZA: zone axis
