# Supplementary material for: Deformation and Failure of Supported Five-Fold Twinned Silver Nanowires under Tensile, Compressive, and Cyclic Loading
Source: ACS Nano. 2025 Aug 21;19(34):30872–88. doi: 10.1021/acsnano.5c05537 (PMC12410372; doi:10.1021/acsnano.5c05537)
Supplement: Supplementary file 1 [file nn5c05537_si_001.pdf]

Supporting Information

Deformation and Failure of Supported Five-Fold Twinned Silver Nanowires Under Tensile, Compressive and Cyclic Loading

*Marco Moninger[1], Lilian Vogl[1,2], Peter Schweizer[1,2], Peter Denninger[1,3], Patricia Smolka[1], Johannes Will[1] and Erdmann Spiecker[1]**

1 Institute of Micro- and Nanostructure Research (IMN) and Center for Nanoanalysis and Electron Microscopy (CENEM), Friedrich-Alexander-Universität Erlangen-Nürnberg, Interdisciplinary Center for Nanostructured Films (IZNF), Cauerstrasse 3, 91058 Erlangen, Germany

now at: Max Planck Institute for Sustainable Materials, 40237 Düsseldorf, Germany

3 now at: Empa, Swiss Federal Laboratories for Materials Science and Technology, Laboratory for Mechanics of Materials & Nanostructures, Thun, Switzerland

Keywords: silver nanowire, fivefold twinning, transparent electrode, buckling, kinks, electron microscopy, correlative microscopy

*Correspondence: erdmann.spiecker@fau.de

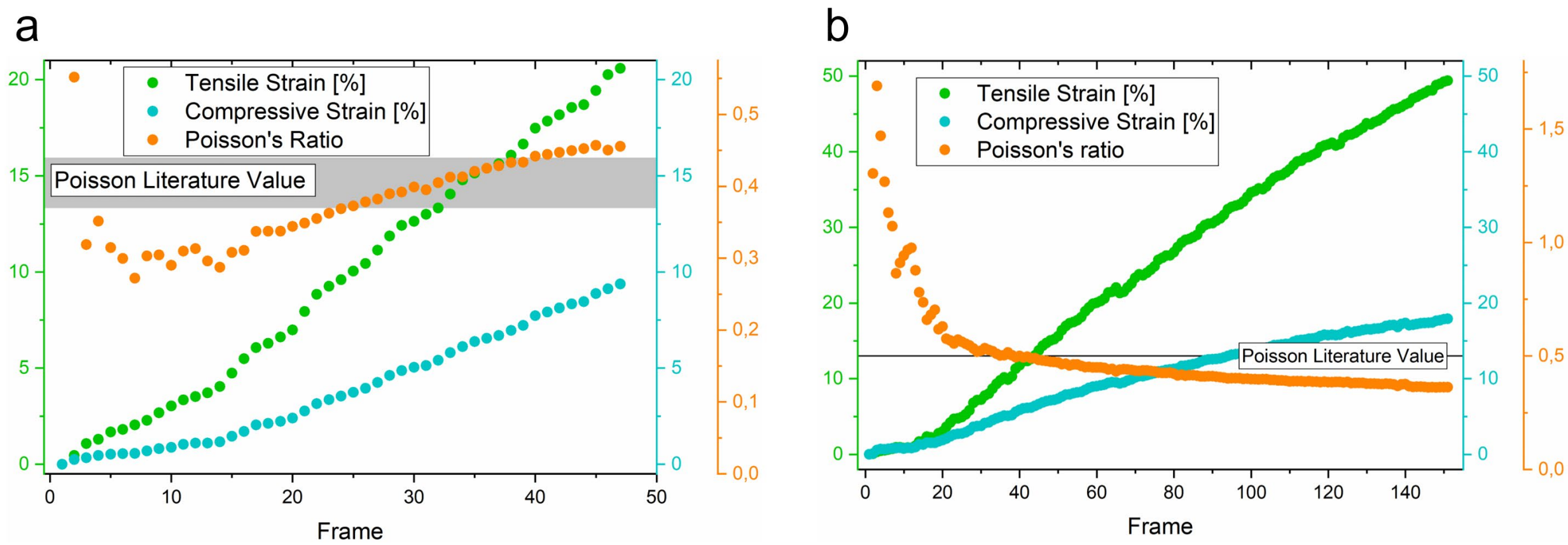

Figure S1. Characterization of the Poisson's ratio of the two polymer substrates, PET (a) and PDMS (b), measured from in situ uniaxial straining tests in the light microscope (LM) using feature tracking (see **Figure S11**). The plotted Poisson's ratios (orange dots) are calculated by dividing the cumulative compressive strain in the transverse direction (blue dots) by the corresponding cumulative tensile strain in the longitudinal (straining) direction (green dots). It is important to note that the plotted Poisson's ratio constitutes an *average value* over the strain range — from the initial zero strain (frame 0) to the total applied strain at the respective frame *n*. This explains the pronounced scatter observed in the Poisson's ratio at the initial frames, which may also be influenced by clamping effects (and potential polymer buckling). For this reason, Poisson's ratio values from the first 15–20 frames are disregarded. At larger strains, a clear trend is observed: the Poisson's ratio of PET increases with increasing strain, while that of PDMS decreases. The grey horizontal bar in (a) indicates the range of Poisson's ratios specified by the manufacturer for this PET [1]. The grey horizontal line in (b) marks a Poisson's ratio of 0.5, specified by the manufacturer[2] of the PDMS and expected for volume conservation under uniaxial straining (at small strains). It is important to emphasize that we do not use the Poisson's ratio in the evaluation of our nanowire straining tests, as we always directly measure the actual longitudinal and transverse strains by tracking features located adjacent to the nanowire on the substrate.

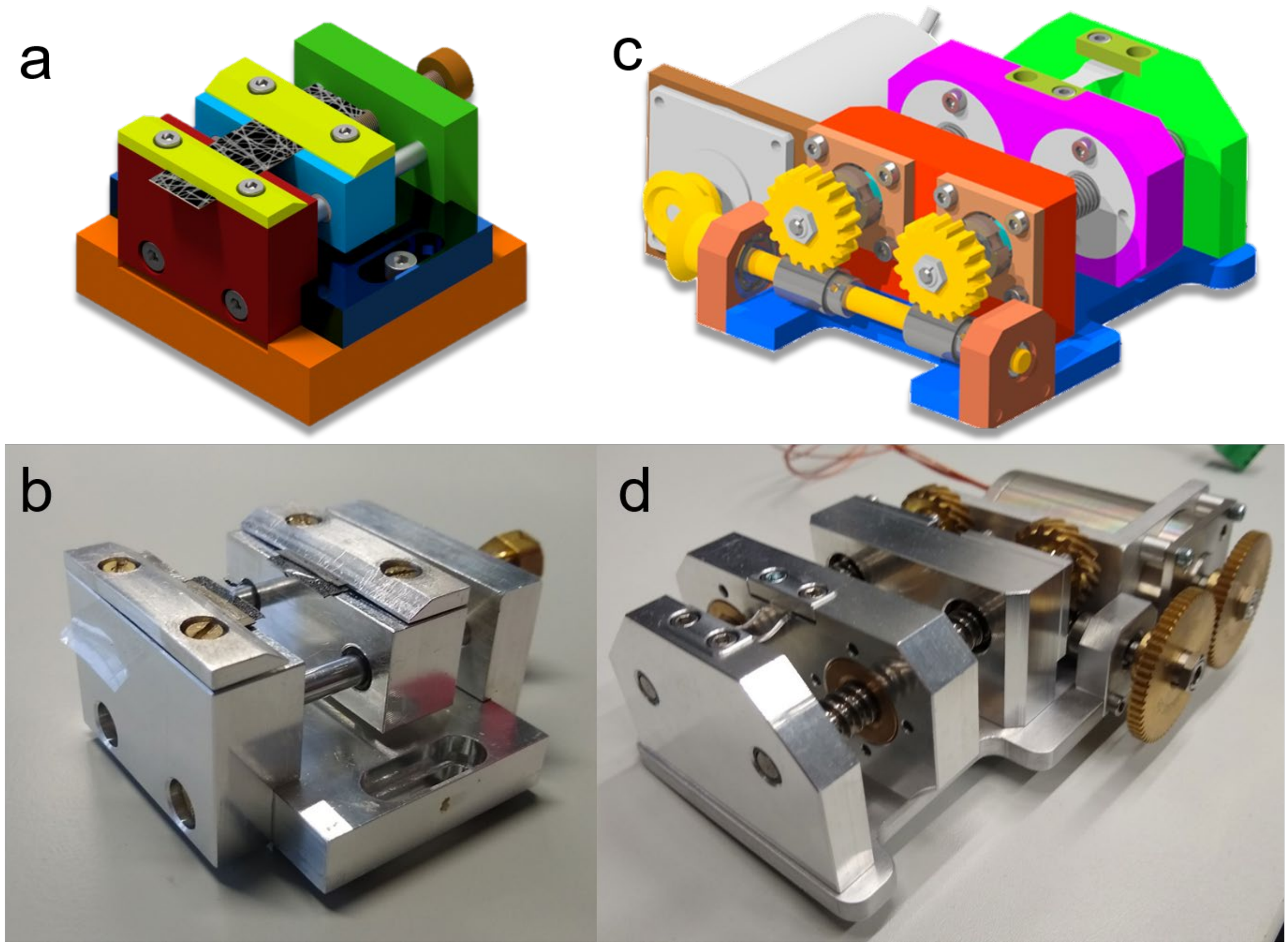

Figure S2. **Schematics and photographs of the two straining stages used for uniaxial mechanical straining of the polymer substrates with nanowires attached to their surfaces.** (a) Schematic of the manual straining stage. The displacement of the clamps is controlled via a screw mechanism. (b) Photograph of the manual straining stage, constructed entirely from aluminum, with brass screws, allowing its use under the light microscope (LM) and insertion into the chamber of a scanning electron microscope (SEM). (c) Schematic illustration of the automated and programmable straining stage, which incorporates a stepper motor. The displacement is generated via a gear mechanism that transfers the motor's rotation to the clamps, where the sample is mounted. (d) Photograph of the automated straining stage, also fully constructed from aluminum, with brass gears, suitable for use under an LM as well as inside an SEM chamber.

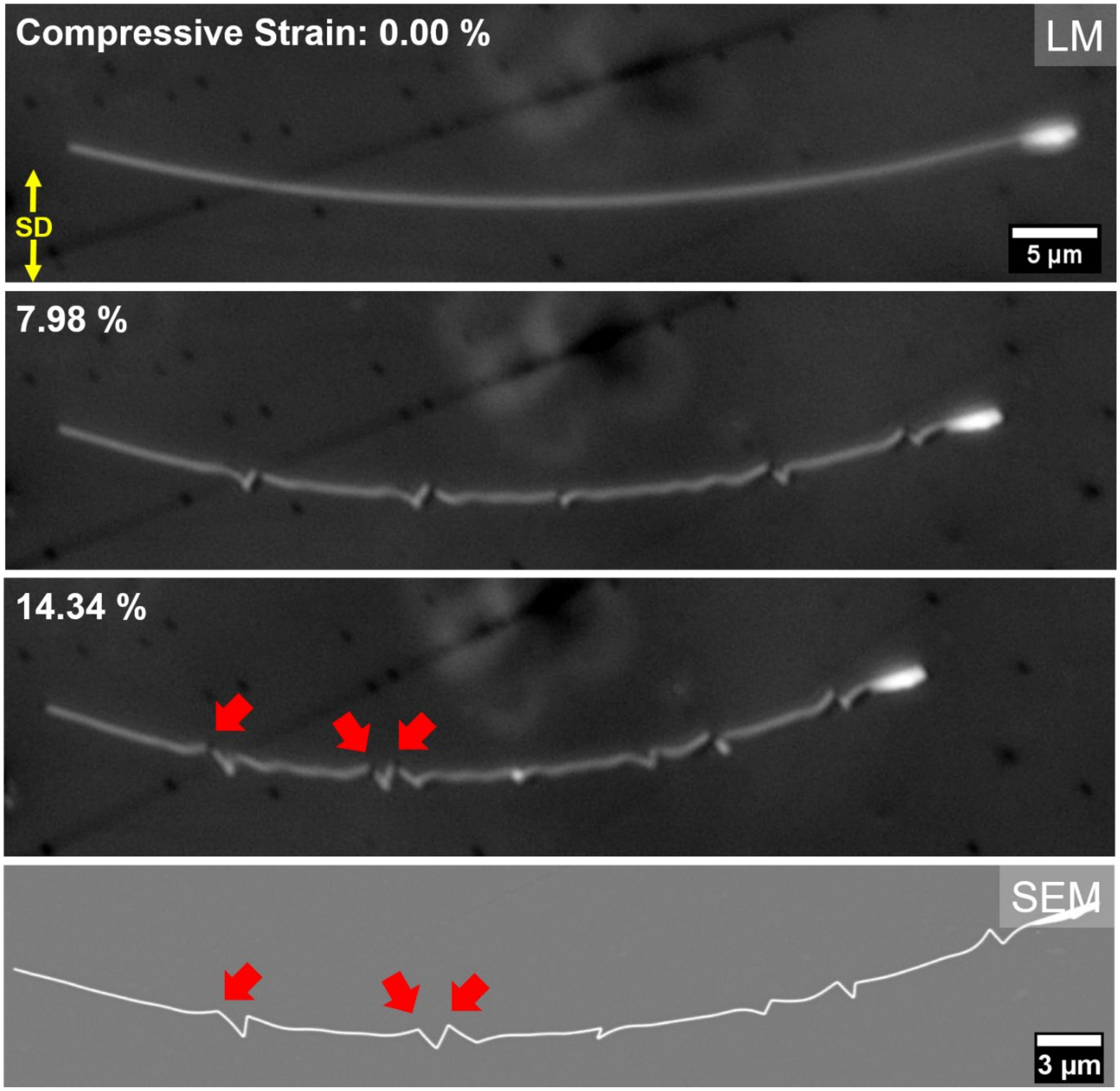

Figure S3. **Structural integrity of a nanowire on PET after severe wire kinking.** Top: LM still frames from a stopped *in situ* compression experiment. The images show the complete wire presented in **Figure 4a** of the main manuscript. The first image displays the pristine wire on the PET substrate. The second image shows the wire after formation of most of the kinks. The third image depicts the wire at the maximum applied strain. Red arrows indicate locations along the wire that appear as fracture gaps in the LM. Bottom: The final image presents the same wire imaged in the SEM, with red arrows marking the corresponding locations. The SEM image clearly reveals that the wire remains continuous across the wire kinks (and thus likely highly conductive); the apparent fracture gaps in the LM are not actual breaks, but rather result from light reflection effects at the wire kinks and the limited resolution of the LM.

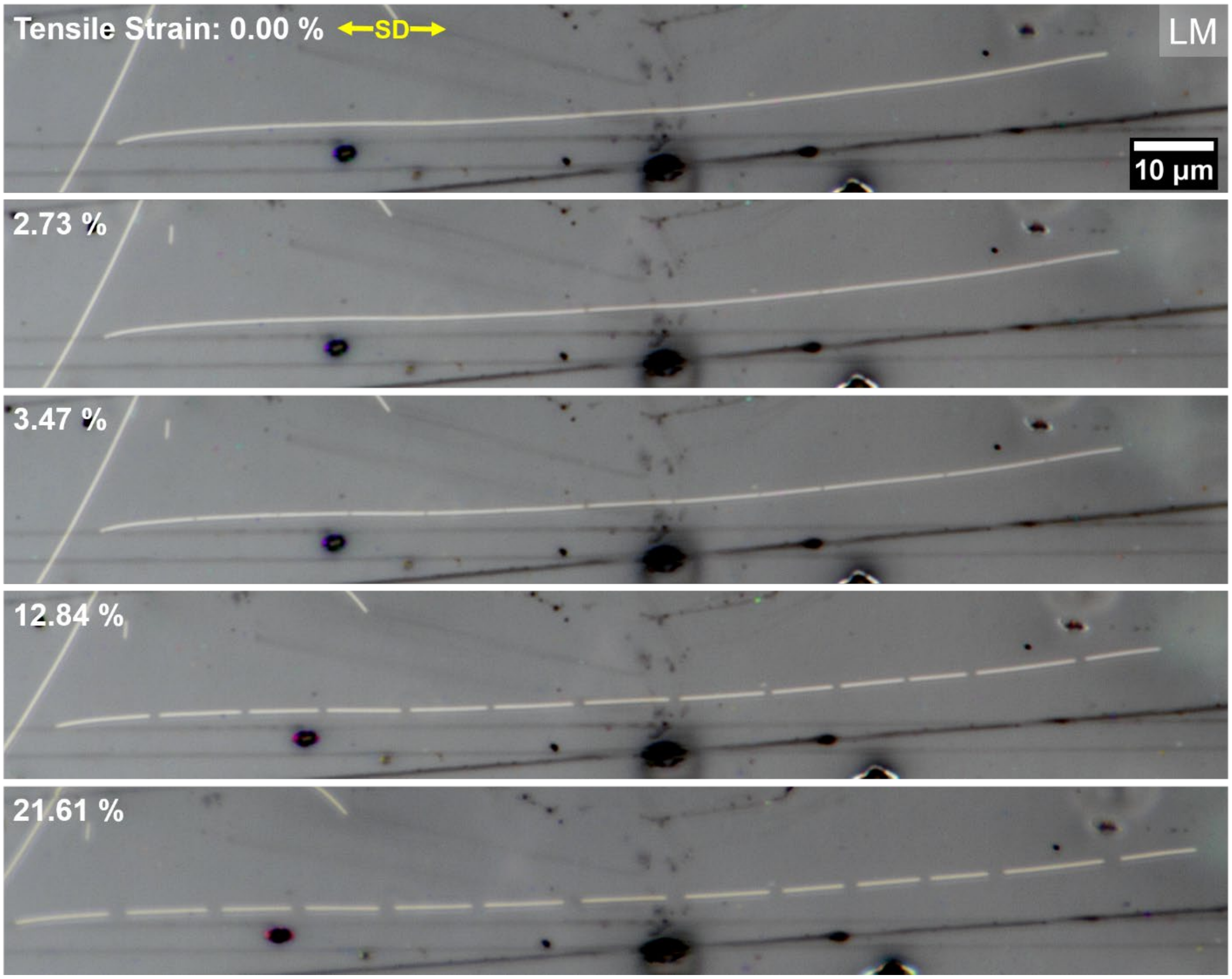

Figure S4. Tensile test of a long AgNW on PET. LM still frames from the stopped *in situ* tensile test shown in **Figure 2** of the main manuscript. The images display the compete wire, including all formed segments. In addition, the uniformity of the segment lengths is clearly illustrated.

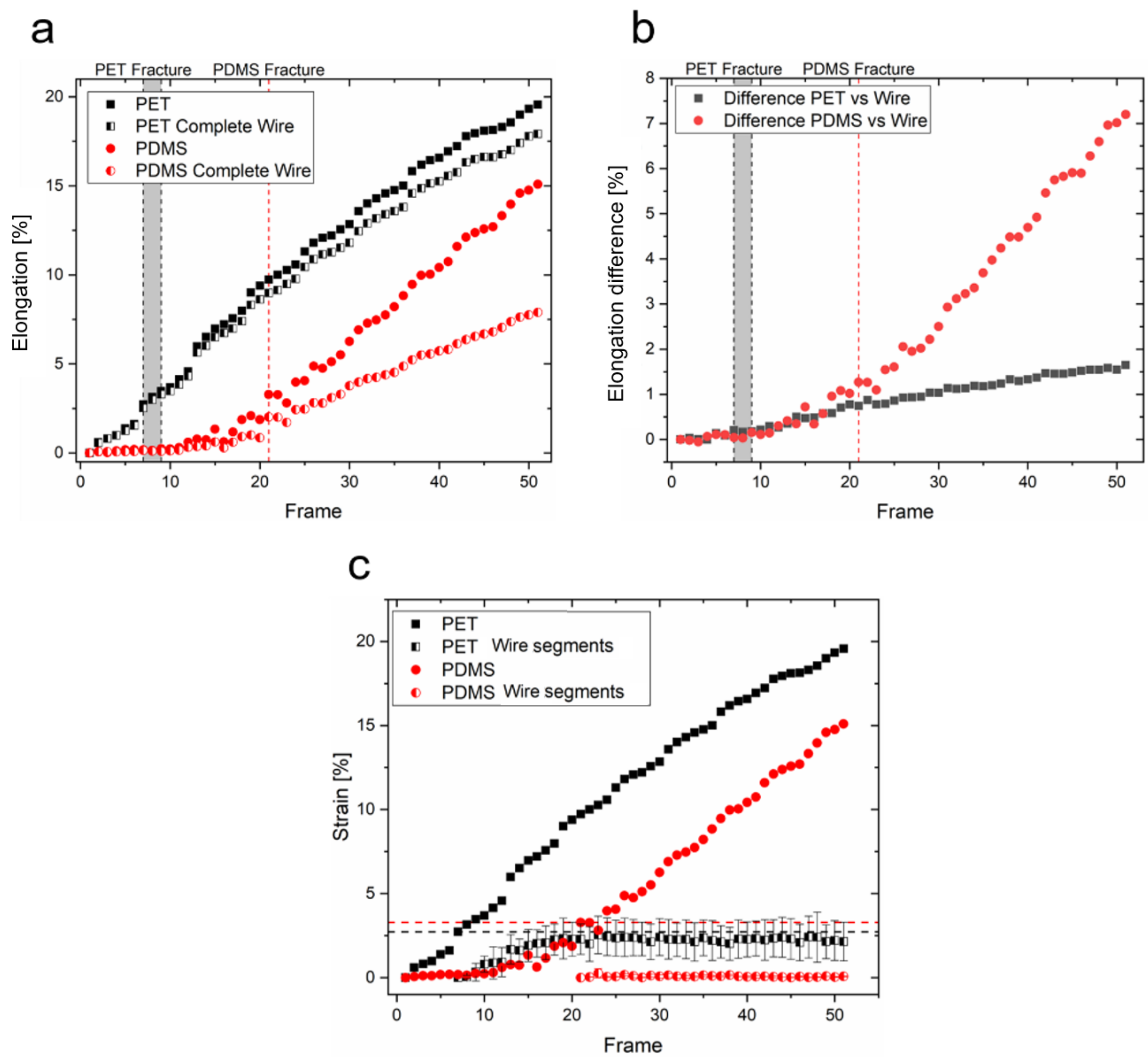

Figure S5. **Evaluation of the strain data from the tensile test shown in Figure 2**. The plots provide an alternative visualization of the data presented in **Figure 3**. a) Comparison of the elongation of the substrate (PET, PDMS) and the corresponding nanowire. For the substrates, the elongation directly reflects the applied strain. In contrast, for the nanowires, elongation represents the relative displacement between the two ends of the wire—independent of fracture events and crack openings occurring along the wire's length, which partially relax the strain in the wire. Unlike in **Figure 3**, the data here are plotted against the frame number of the tensile test. The black dotted lines and the grey bar indicate the ranges of LM images in which fracturing occurred for the nanowire on PET. For the wire on PDMS, only a single fracture event was observed, marked

by the red dotted line. b) Difference in elongation between the substrate and the corresponding nanowire for the two systems (PET and PDMS). As straining progresses, the difference in elongation increases for both cases, but the effect is significantly more pronounced for PDMS than for PET. This suggests substantial slippage of the nanowire at its ends on the PDMS substrate, which limits the strain transmitted to the wire. As a result, only a single crack forms in the nanowire on PDMS. c) Comparison of the substrate strain (filled symbols, as shown in Figure part a) with the mean strain of the wire segments after fracturing. The black and red dotted lines indicate the substrate strain levels at which the first fracture occurs in the nanowires on PET and PDMS, respectively. On PDMS, pronounced slippage of the wire at the ends of the two segments formed after fracture prevents effective strain transfer from the substrate to the wire segments, thereby suppressing further fracture events. On PET, slippage at the segment ends is significantly less. However, as multiple short wire segments form, the cumulative slippage remains sufficient to limit strain transfer to levels below the critical threshold (indicated by the black dotted line) necessary to trigger further fractures and wire fragmentation.

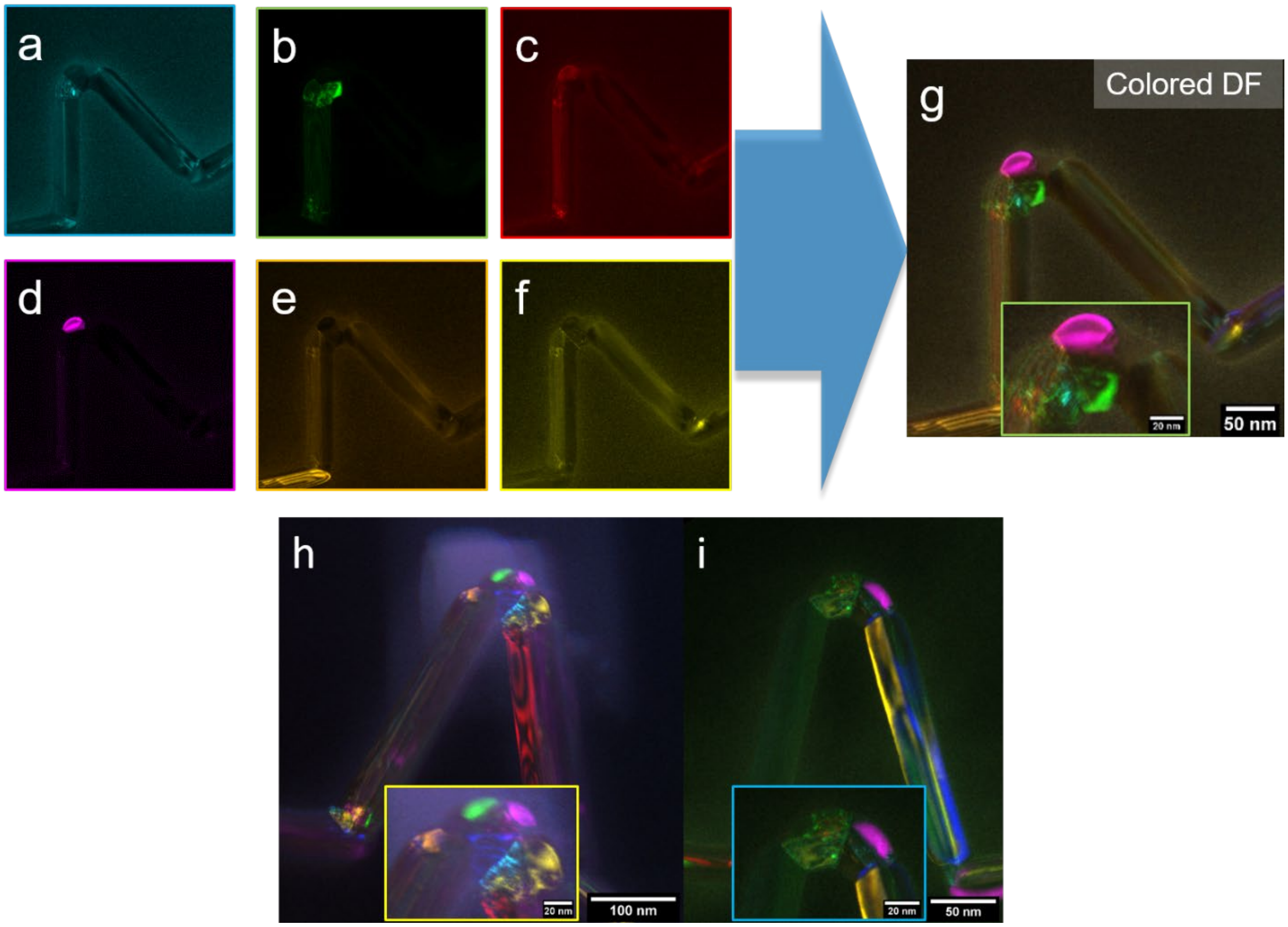

Figure S6. **Dark-field TEM analysis of wire kinks in heavily deformed AgNWs**. a-f) Colored dark-field TEM images highlighting different parts of the nanowire, each acquired using a distinct Bragg reflection. g) False-color image generated by combining the images shown in a-f, clearly revealing the presence of small crystallites formed at the very tip of the central wire kink. h, i) False-color images of two additional kink structures, exhibiting similar crystallite formation. The emergence of small crystallites on the outer side of the wire kinks is characteristic of kinks with acute angles. Notably, some of these grains appear nearly defect-free.

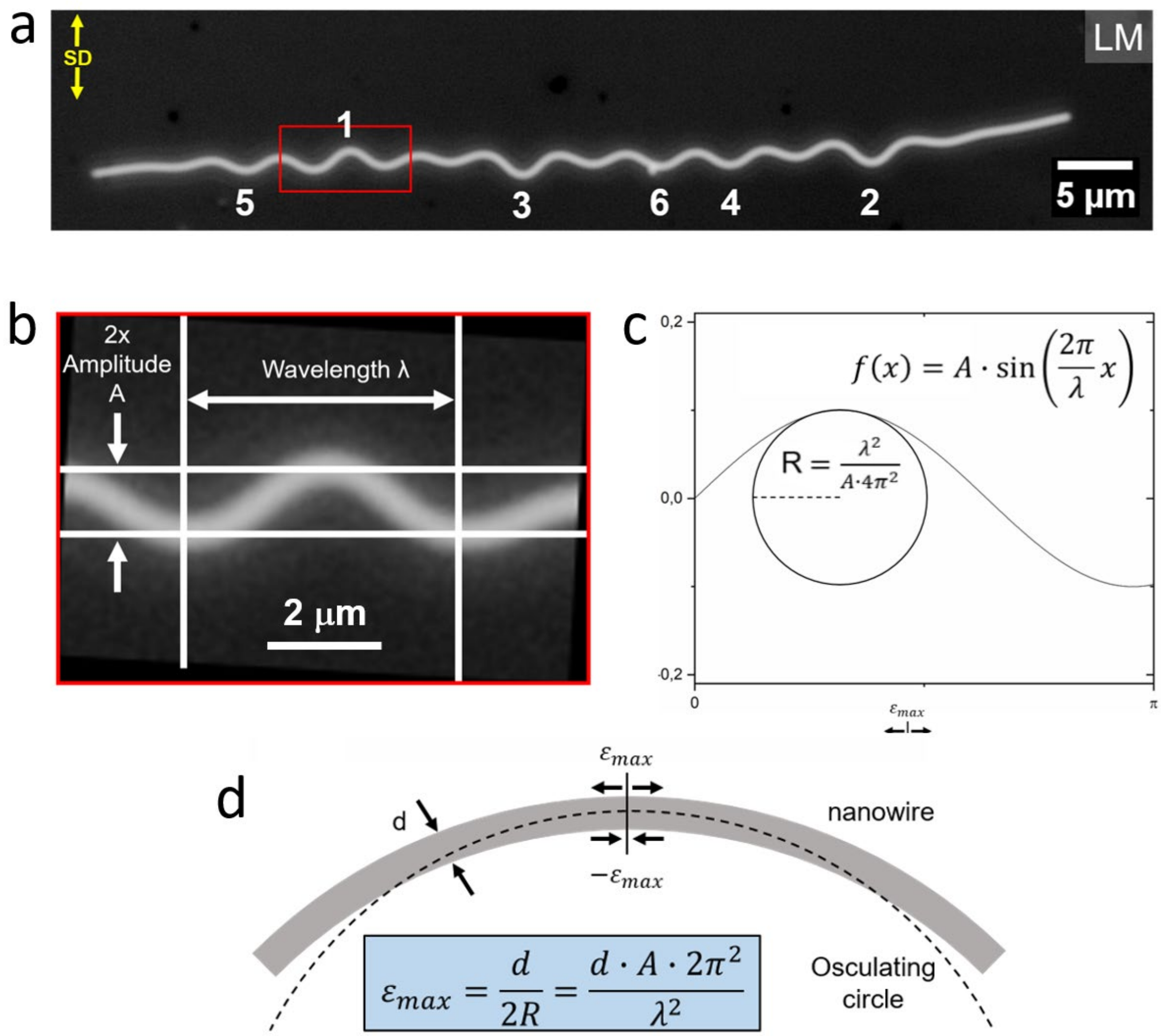

Figure S7. **Evaluation of the critical strain ε_{max} at the nanowire surface at the onset of kink formation**. a) Light microscopy (LM) image of an undulated AgNW on PDMS, extracted from an LM image series recorded during a compression experiment. The image captures the final state before the onset of plastic deformation via kink formation in the undulation period labeled "1". b) Enlarged section of the image illustrating the measurement of the undulation's wavelength λ and amplitude A. Note that the true width (diameter) of the nanowire is significantly smaller than its apparent width in the LM image. The period and amplitude are determined by assuming that the nanowire lies at the center of the LM contrast. c) Modelling of the nanowire undulation by a sinusoidal function (see **Figure 7**)

$$f(x) = A \cdot sin\left(\frac{2\pi}{\lambda}x\right).$$

The radius of curvature of the osculating circle is given by

$$R(x) = \left| \frac{(1 + f'(x)^2)^{\frac{2}{3}}}{f''(x)} \right|$$

and reaches its maximum value

$$R = \frac{\lambda^2}{A \cdot 4\pi^2}$$

at the point of maximum amplitude. d) The maximum tensile strain ε_{max} at the outer nanowire surface can be calculated from the radius of curvature R and the true nanowire diameter d using

$$\varepsilon_{max} = \frac{d}{2R} = \frac{d \cdot A \cdot 2\pi^2}{\lambda^2}.$$

This relation assumes that the true nanowire diameter is much smaller than the local radius of curvature, i.e., $d \ll R$, which is a good approximation for all cases investigated in the present study. Note, that under this condition the maximum compressive strain at the inner surface of the nanowire is $-\varepsilon_{max}$.

For the nanowire diameter d, we use the mean wire width of 57.4 nm, as determined by HAADF-STEM measurements (see **Figure S8**). The standard deviation of the nanowire width distribution is 7.1 nm, corresponding to a relative variation of 13%. Consequently, the same relative uncertainty applies to the calculated maximum tensile and compressive strain at the nanowire surface. An alternative approach would have been to measure the diameter of each individual nanowire after compressive testing using SEM. However, this method would require a conductive coating and suffer from limited image quality due to the electron beam sensitivity of PDMS. The resulting uncertainty in the measured wire diameter would be in the range of 5–10 nm. For these reasons, we opted to use the mean wire diameter 57.4 nm determined by HAADF-STEM for calculating ε_{max}.

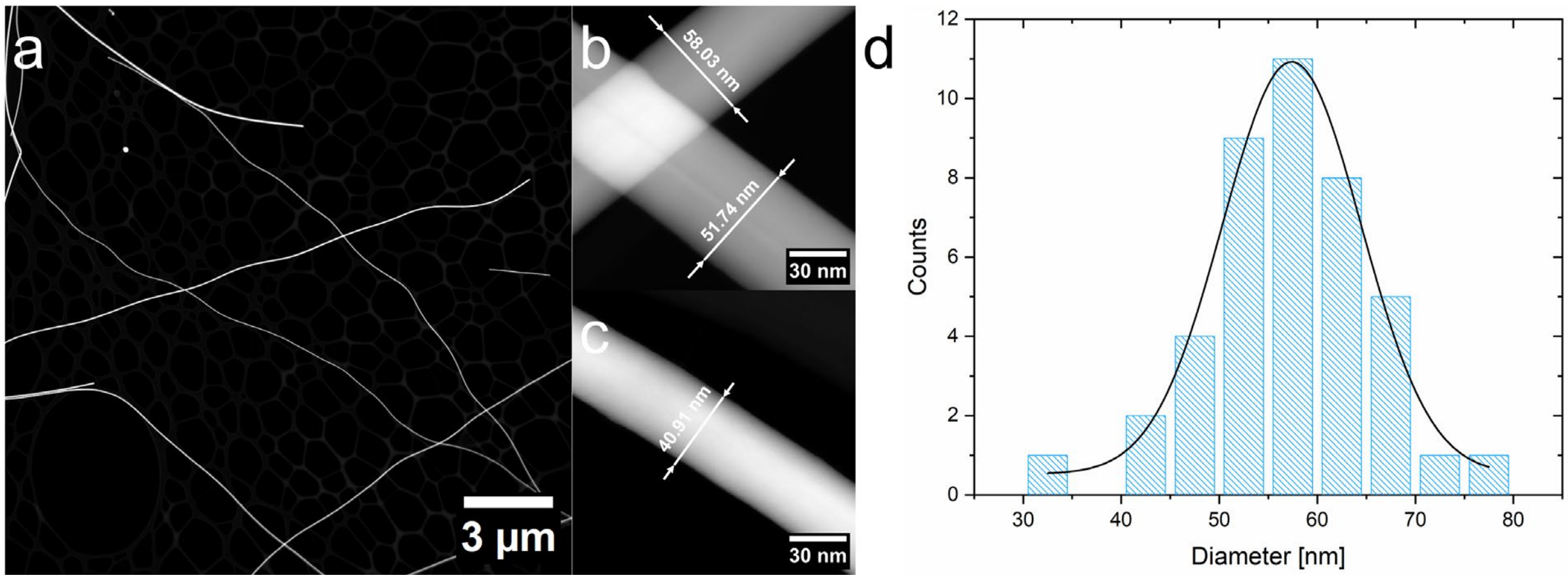

Figure S8. **Diameter distribution of the nanowires used in the LM studies** (nominally 100 nm nanowires from ACS materials; see Methods in main manuscript)**.** a) Low magnification HAADF-STEM image of silver nanowires from the batch used, dispersed on a lacey carbon TEM grid. b, c) Representative HAADF-STEM images used for diameter measurements of individual nanowires. d) Histogram showing the diameter distribution of 42 measured nanowires, which is well described by a normal distribution. The mean wire diameter is $57.4 \pm 7.1\ nm$.

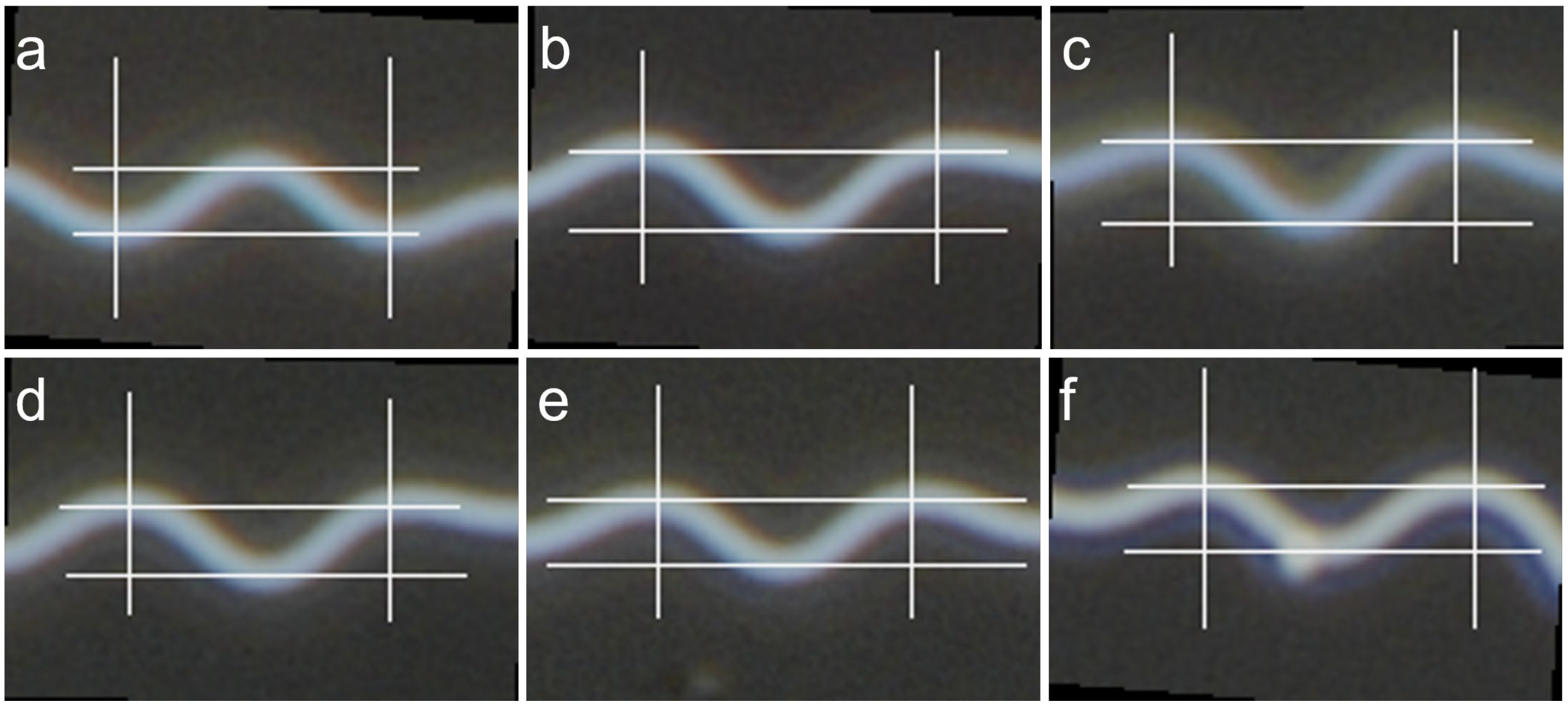

Figure S9. Evaluation of the six wire undulations marked in Figure S7a where kink formation was observed. Each LM image depicts the respective undulation shortly before the onset of kink formation. Evaluation of the local amplitude A and wavelength λ enables estimation of the critical surface strain ε_{max} at which plastic deformation occurs (cf. figure caption of **Figure S7**). The results of this analysis are compiled in **Table S1**. Details of the evaluated sites are as follows: a) Undulation at Site 1, recorded at 7.49% compressive substrate strain, immediately prior to kink formation. b) Site 2, at 7.49% strain. c) Site 3, at 7.76%. d) Site 4, at 9.13. e) Site 5, at 11.54%. f) Site 6, at 15.77%.

Table S1. **Values of critical surface strain ε_{max} at the onset of plastic deformation.** The table presents the critical surface strain ε_{max} at the nanowire surface associated with the onset of permanent plastic deformation through kink formation. The data correspond to the six deformation sites along the nanowire shown in **Figure S7**. Wavelength λ and amplitude A were determined as illustrated in **Figure S9**. For the estimation of ε_{max}, a mean wire diameter $d = 57.4\,nm$ was assumed (cf. **Figure S8**)

Undulation (Figures S7, S8)	Wavelength λ [µm]	Amplitude A [µm]	Critical strain ε_{max} [%]
Site 1	4.73	0.57	2.86
Site 2	5.07	0.68	3.00
Site 3	4.88	0.71	3.37
Site 4	4.48	0.60	3.35
Site 5	4.38	0.56	3.32
Site 6	4.67	0.56	2.90

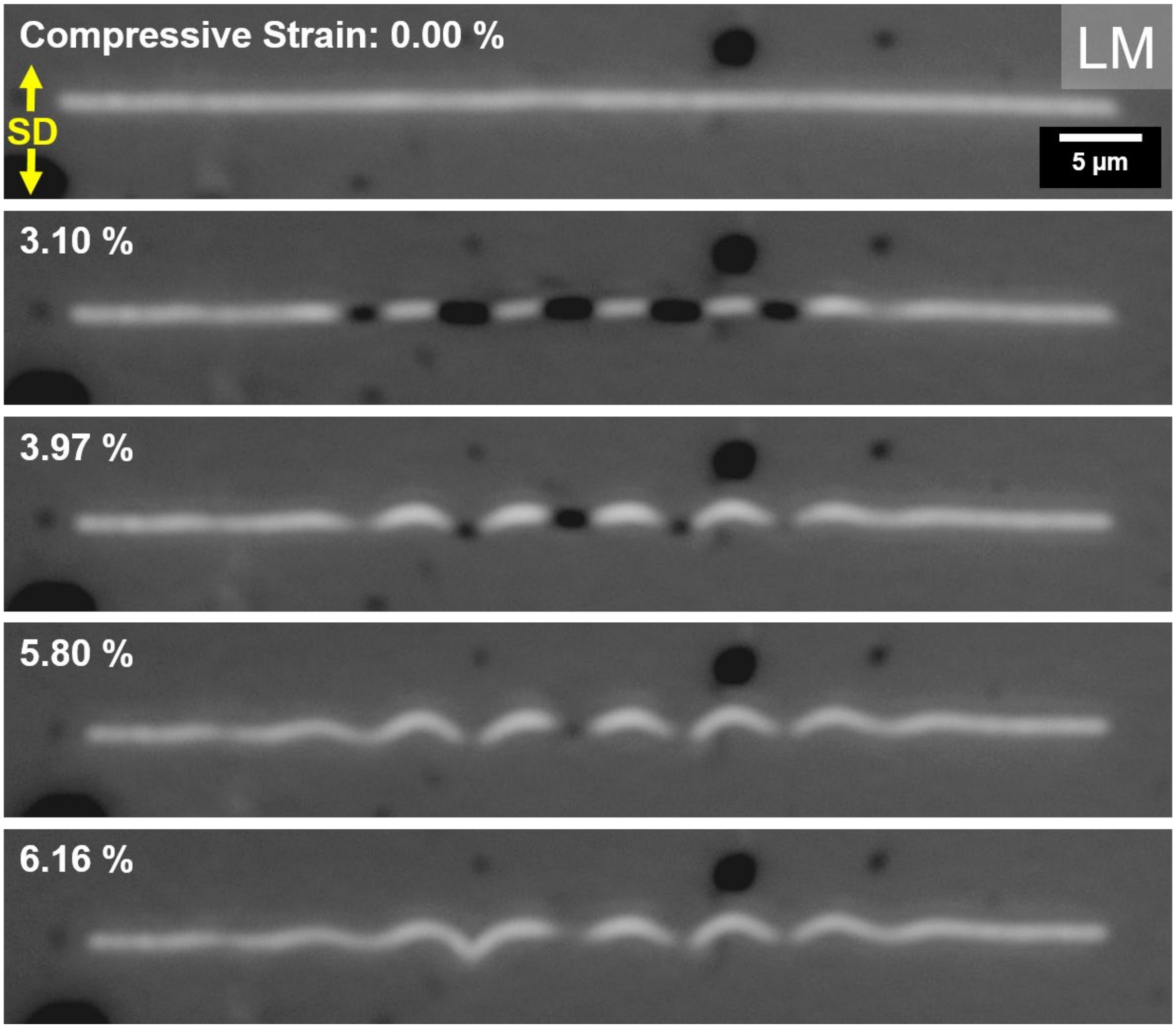

Figure S10. **Out-of-plane buckling of a nanowire on PDMS**. During compressive loading of the nanowire, a periodic bright/dark contrast emerges along its length at a substrate strain of 3.10%. This contrast is indicative of out-of-plane buckling, as the wire maintains a straight appearance despite the significant applied compressive strain. This interpretation is further supported by subsequent frames, where segments of the wire rotate back into the plane, transitioning into the characteristic morphology observed in all other compression experiments: a uniformly bright wire exhibiting sinusoidal in-plane undulations. These observations demonstrate that the initial out-of-plane buckling gradually evolved into conventional in-plane buckling. Notably, this phenomenon was only observed for a nanowire on PDMS, which is consistent with the lower adhesion of the wires to this substrate compared to PET. The reduced adhesion also facilitates increased slippage of the nanowires, further supporting the distinct deformation behavior on PDMS.

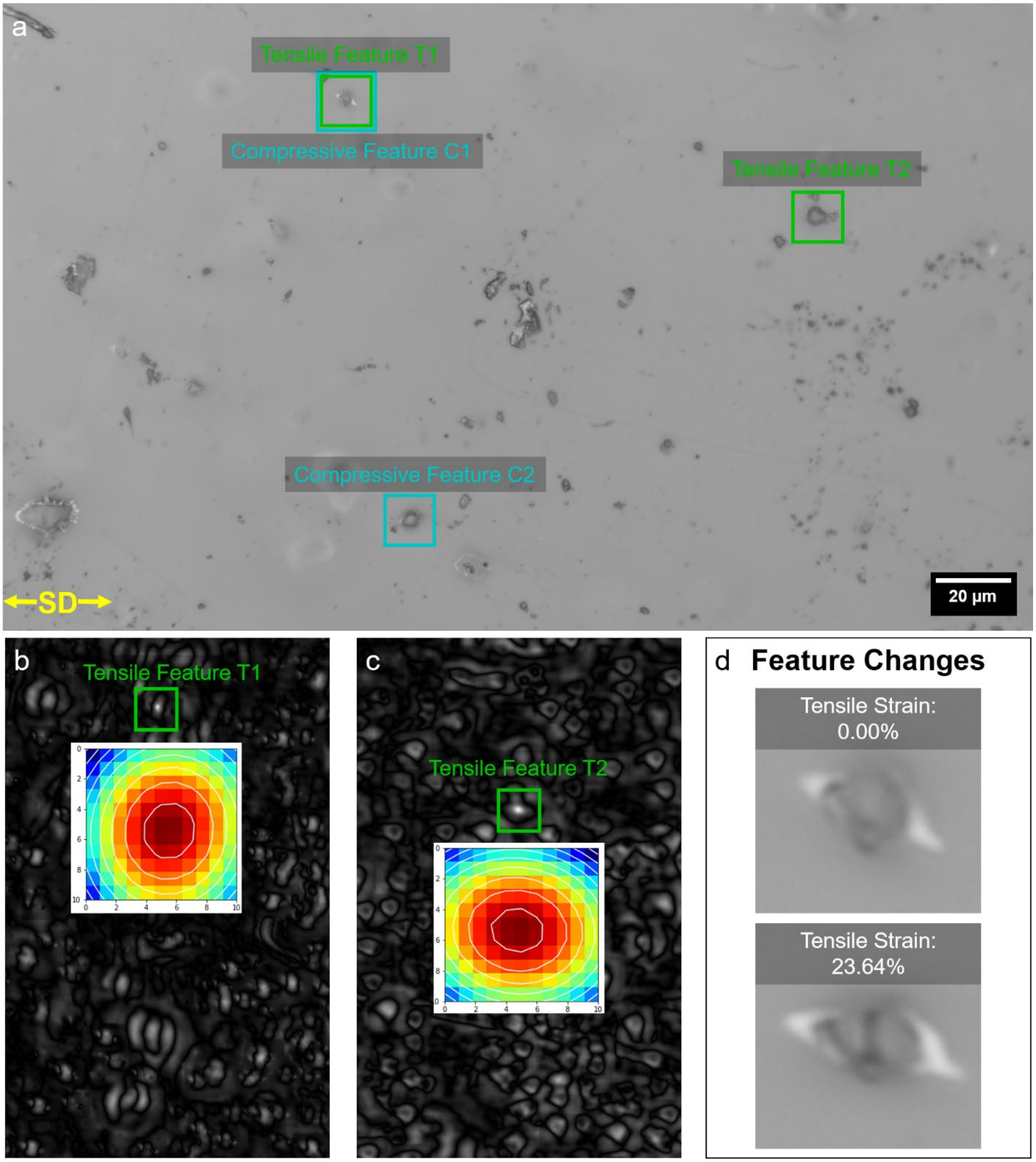

Figure S11. Illustration of the feature tracking algorithm. a) LM image of PET surface indicating the feature pairs used for feature tracking during uniaxial tensile straining and subsequent calculation of tensile/compressive substrate strain. b+c) Exemplary cross-correlation maps of two features T1 and T2, including two-dimensional Gaussian fits to the correlation maxima. d)

Representative change in the appearance of a selected surface feature in LM before loading (0 % strain) and at maximum tensile strain (here 23.64 %).

Figure S11 illustrates the algorithm employed for tracking and precisely locating arbitrarily selected surface features. The algorithm was developed by Peter Schweizer and is discussed in further detail in Ref. [3]. The method involves computing the correlation function between the reference feature (taken from the LM image at zero strain) and the image under analysis. This is accomplished by virtually translating the feature across the image and evaluating a correlation coefficient at each position using a predefined comparison metric. Here, the normalized cross-correlation coefficient was identified as the most appropriate matching criterion. It is computed by summing the products of corresponding pixel intensities between the template and the image at each position, followed by normalization with respect to the overall intensity values. The resulting coefficient quantifies the degree of similarity between the template and the local image region. Consequently, the brightest pixel in the resulting correlation map corresponds to the position of the best match for the template. **Figure S11 b** and **S11 c** present typical correlation maps for the two features T1 and T2, derived from randomly selected images within a strain series. The presence of multiple bright spots in the maps indicates that the selected features also exhibit similarities to other structures on the surface. The more distinct a chosen feature is, the better the resulting correlation and the more accurate the subsequent position determination. Nevertheless, a clear maximum in the correlation could be identified in each case. By identifying the position of the most intense pixel in the correlation map, the location of the template within the image can be determined. However, this approach limits the positional accuracy to the pixel size, which was 59 nm in most LM images. To overcome this limitation and achieve sub-pixel precision, a two-dimensional Gaussian function was fitted to the correlation peaks, as illustrated in the insets of **Figures S11b** and **S11c**. With the refined position data, precise distances between feature pairs can be computed. By monitoring their positions across sequential images, relative displacements—and thus local strains—can be derived. This approach can be extended to multiple feature pairs distributed across the sample, enabling the determination of strain over the entire visible area without the need for external strain measurements. For optimal accuracy, feature pairs should ideally be aligned along the same horizontal or vertical axis, as only the x- or y-coordinates are used in the displacement. **Figure S11 d** illustrates the changes that individual features can undergo during the straining process. As shown, a slight elongation is observed at 23.64% tensile strain.

Such deformation may introduce minor inaccuracies in determining the exact feature position. However, the application of two-dimensional Gaussian fitting helps to partially compensate for these deviations. Ideally, features that remain morphologically stable throughout the deformation should be selected for tracking to ensure the highest positional accuracy.

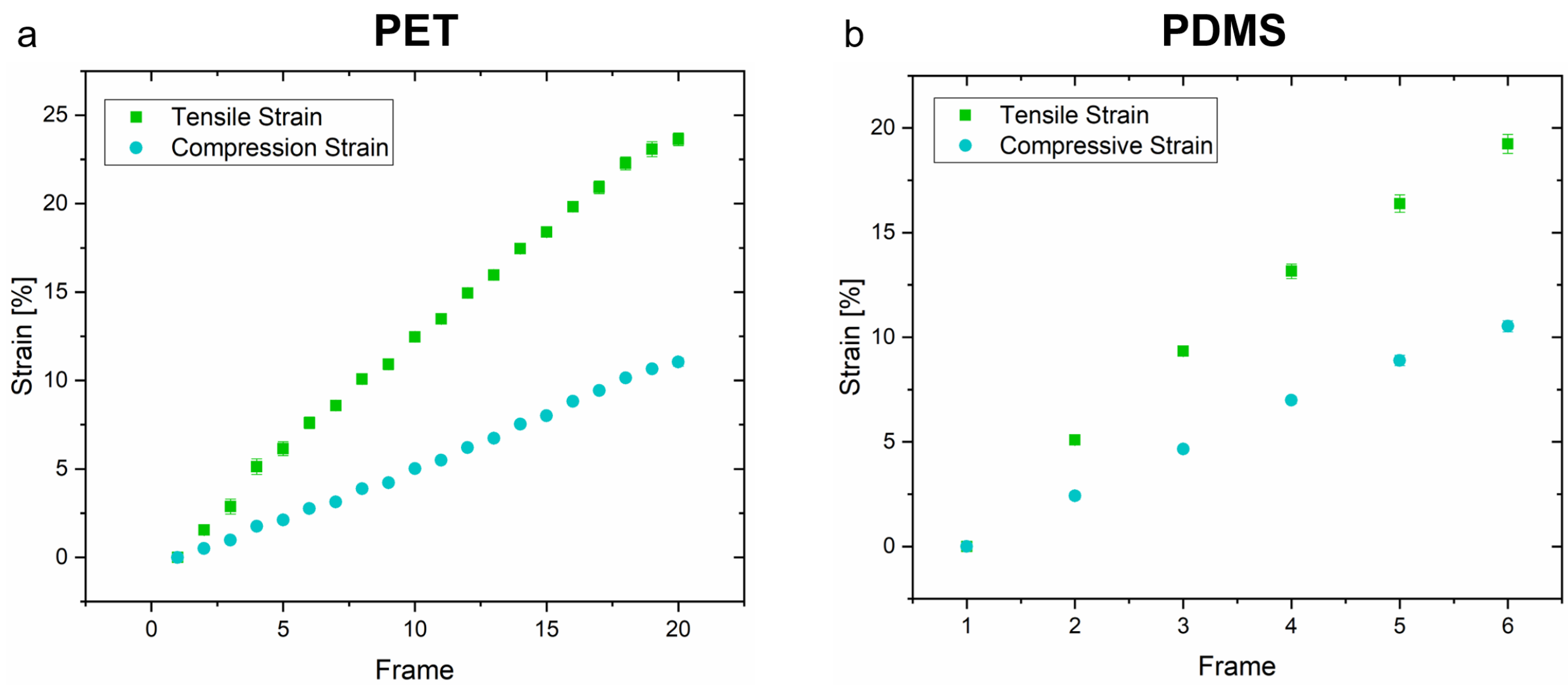

Figure S12. Tensile and compressive strain curves for PET and PDMS, calculated using the feature tracking algorithm applied to multiple feature pairs. a) Strain curves from a tensile test on a PET sample, based on five feature pairs in the tensile direction and five in the perpendicular compressive direction. b) Strain curves from a tensile test on a PDMS sample, using three feature pairs for each direction. In both cases, the mean strain values are plotted along with their corresponding standard deviations. The minimal standard deviations observed indicate a highly uniform strain distribution across the field of view.

Table S2. Initial length of all tensile-tested AgNWs on PET, the substrate strain value at the first occurrence of fracture, and the number of segments at the end of straining.

Wire	Initial wire length [µm]	Tensile substrate strain at first fracture [%]	Number of segments
PET_T_01 (Figure 2)	123.9	2.73	13
PET_T_02 (Figure S13)	36.0	3.46	5
PET_T_03 (Figure S14)	32.1	3.02	5
PET_T_04 (Figure S15)	46.2	2.51	8
PET_T_05 (Figure S16)	54.4	3.02	17
PET_T_06 (Figure S17)	24.0	3.89	2
PET_T_07 (Figure S18)	75.1	2.32	5

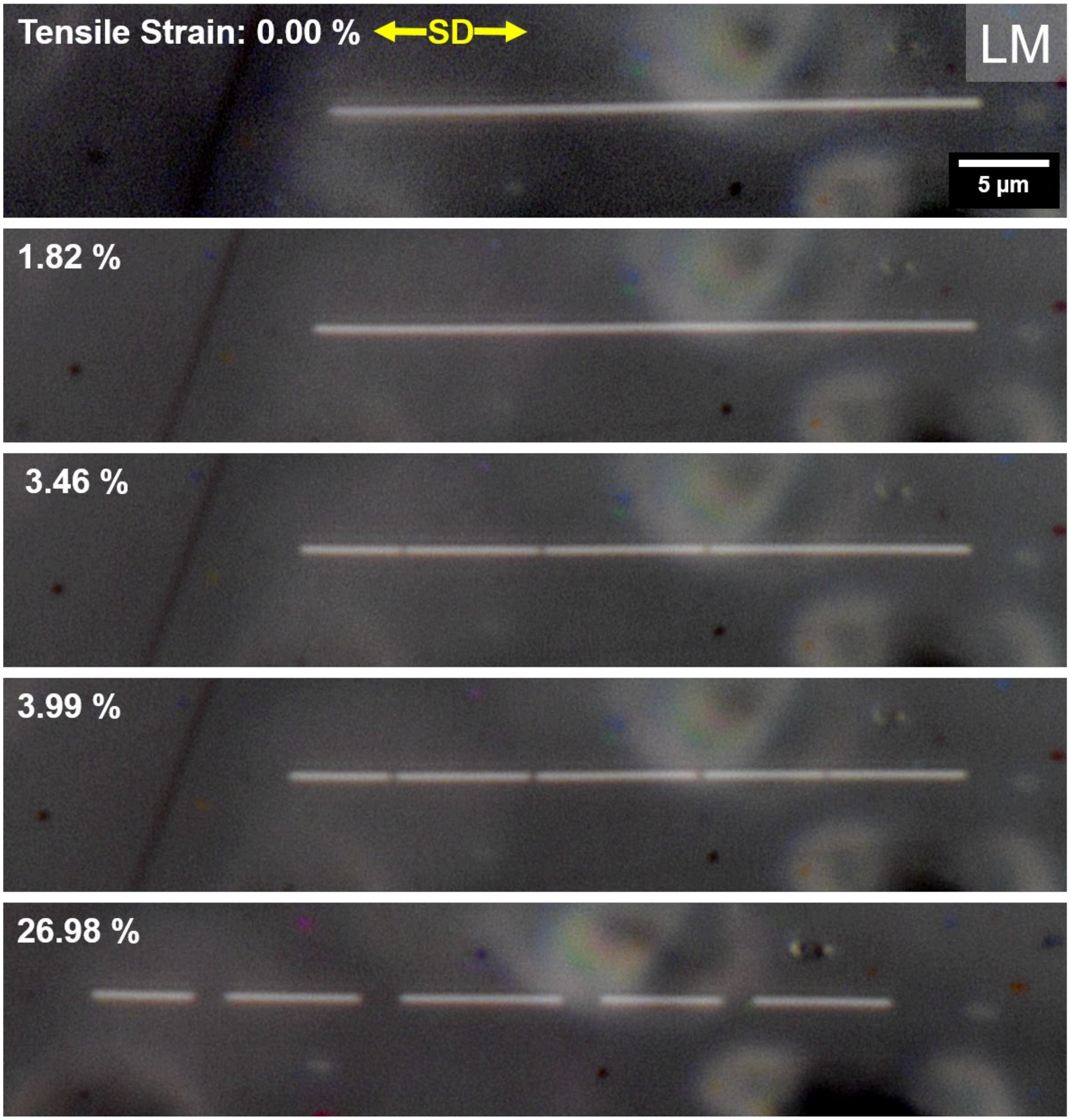

Figure S13. LM still frames from an *in situ* tensile straining test of an AgNW located on PET (PET_T_02 from **Table S2**). The straining direction of the substrate is indicated in yellow. The wire shows the characteristic onset of tensile failure at a tensile substrate strain of 3.46 %, followed by segmentation into five distinct segments.

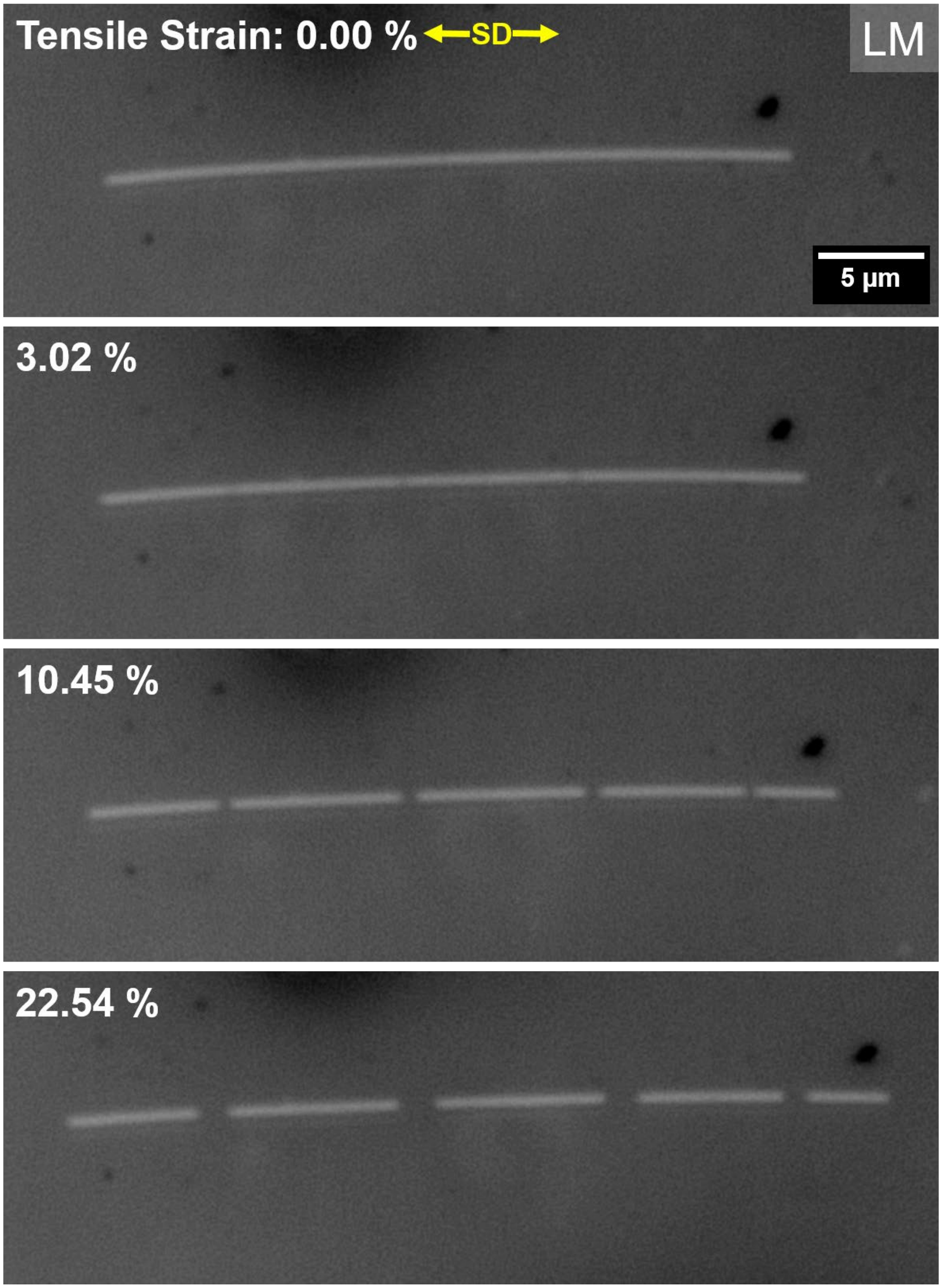

Figure S14. LM still frames from an *in situ* tensile straining test of an AgNW located on PET (PET_T_03 from **Table S2**). The straining direction of the substrate is indicated in yellow. The wire shows the characteristic onset of tensile failure at a tensile substrate strain of 3.02 %, followed by segmentation into five distinct segments.

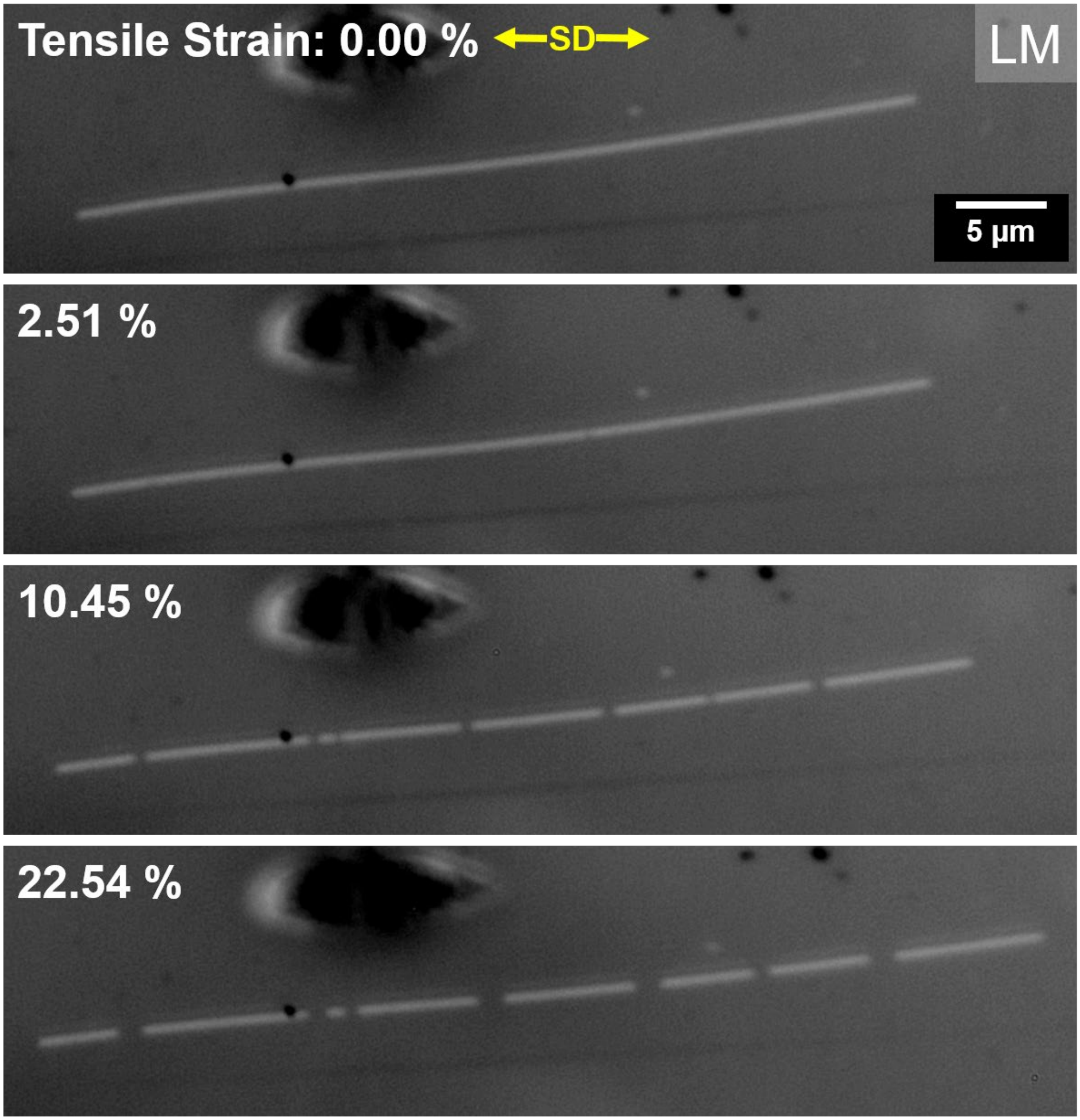

Figure S15. LM still frames from an *in situ* tensile straining test of an AgNW located on PET (PET_T_04 from **Table S2**). The straining direction of the substrate is indicated in yellow. The wire shows the characteristic onset of tensile failure at a tensile substrate strain of 2.51 %, followed by segmentation into eight distinct segments.

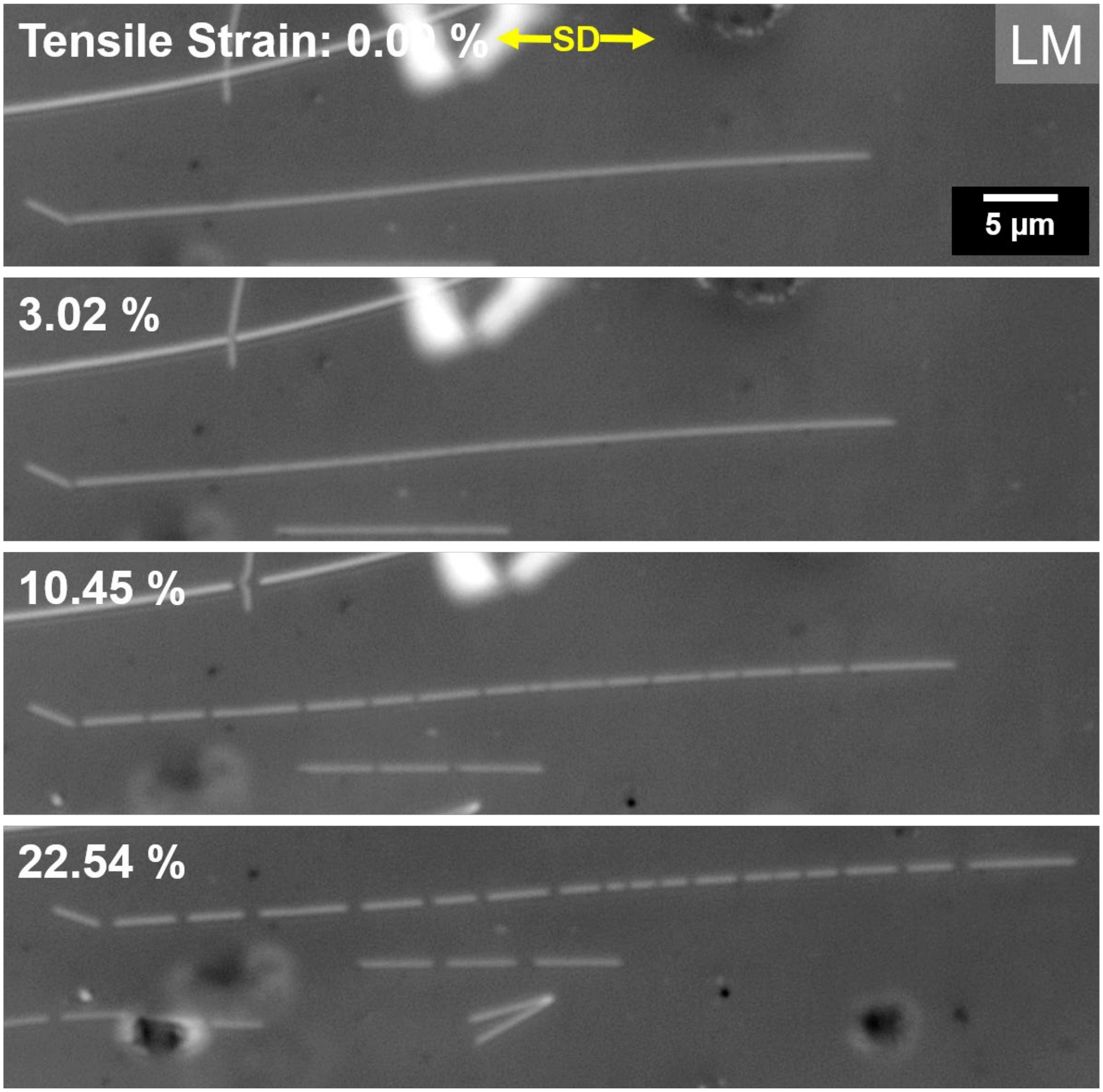

Figure S16. LM still frames from an *in situ* tensile straining test of an AgNW located on PET (PET_T_05 from **Table S2**). The straining direction of the substrate is indicated in yellow. The wire shows the characteristic onset of tensile failure at a tensile substrate strain of 3.46 %, followed by segmentation into 17 distinct segments.

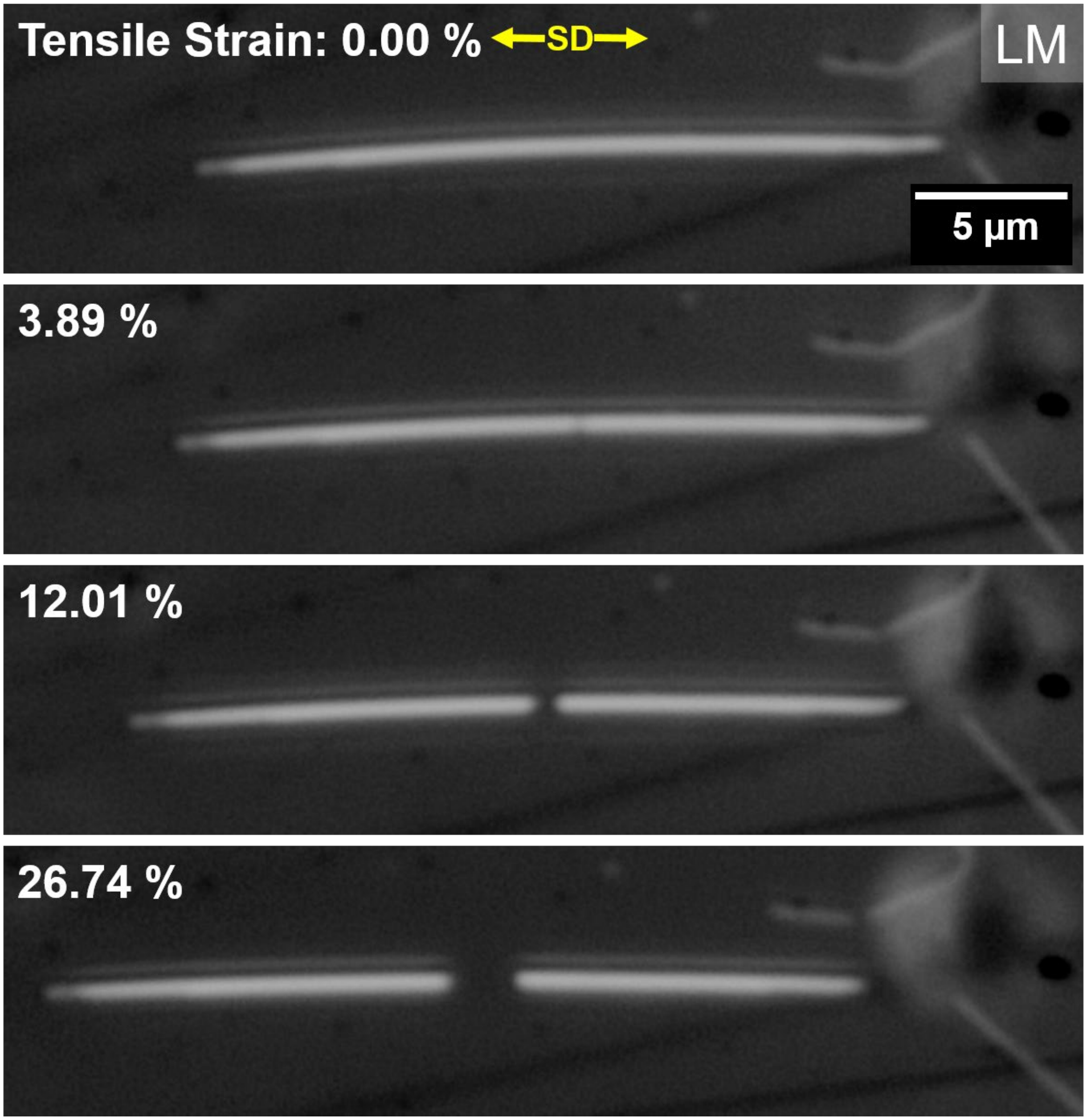

Figure S17. LM still frames from an *in situ* tensile straining test of an AgNW located on PET (PET_T_06 from **Table S2**). The straining direction of the substrate is indicated in yellow. The wire shows the characteristic onset of tensile failure at a tensile substrate strain of 3.89 %, followed by segmentation into two distinct segments.

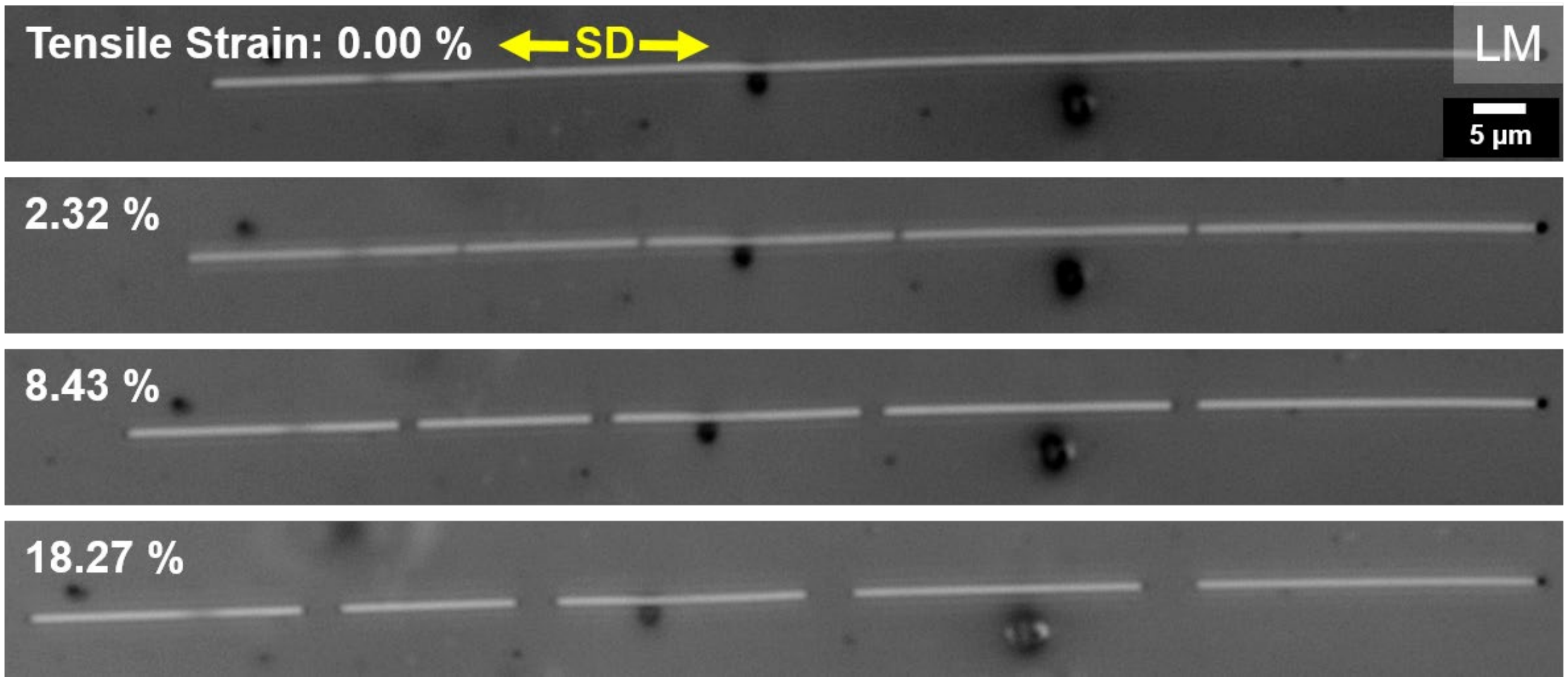

Figure S18. LM still frames from an *in situ* tensile straining test of an AgNW located on PET (PET_T_07 from **Table S2**). The straining direction of the substrate is indicated in yellow. The wire exhibits the characteristic onset of tensile failure at a tensile substrate strain of 2.32 %, followed by segmentation into five distinct segments.

Table S3. Initial length of all tensile tested AgNWs on PDMS, the substrate strain value at the first occurrence of fracture, and the number of segments at the end of straining.

Wire	Initial wire length [μm]	Tensile substrate strain at first fracture [%]	Number of segments
PDMS_T_01 (Figure 2)	95.7	3.28	2
PDMS_T_02 (Figure S19)	43.8	3.21	2
PDMS_T_03 (Figure S19)	32.70	3.21	2
PDMS_T_04 (Figure S20)	107.2	0.24	2

The comparatively low strain value at the onset of fracture in the case of the wire PDMS_T_04 likely results from a defect introduced during coating or sample handling. After this initial failure, the wire exhibited behavior analogous to that shown in **Figure 2a**, with no subsequent fractures but a clear and progressive opening of the initial crack.

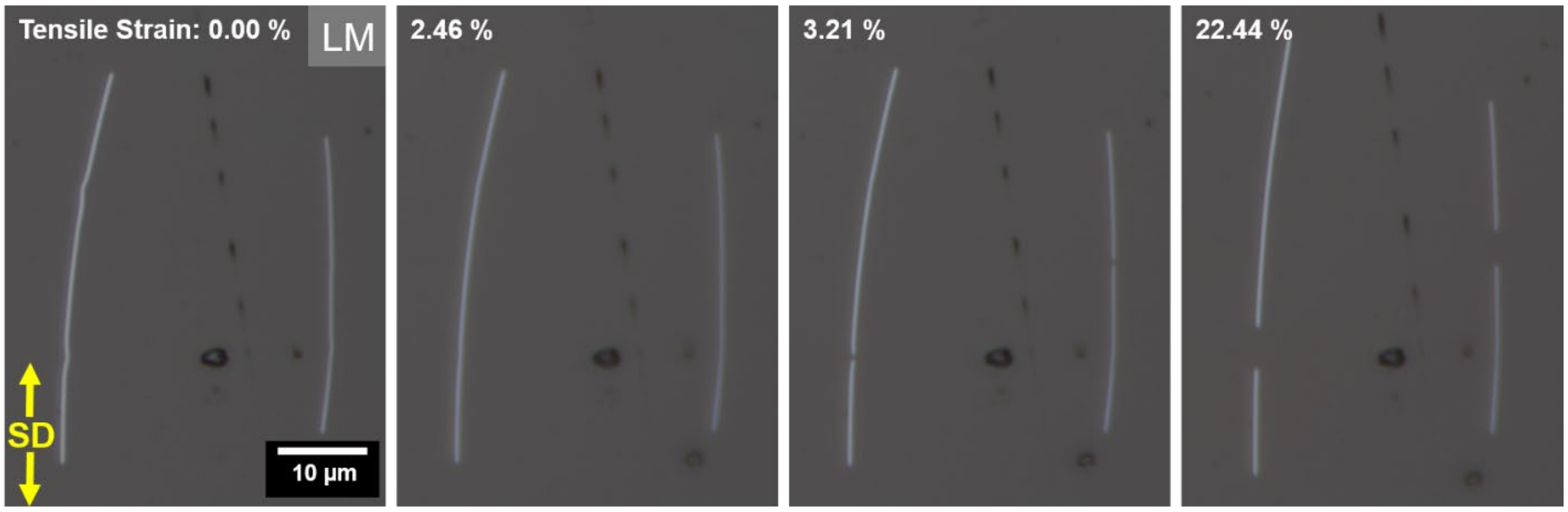

Figure S19. LM still frames from an *in situ* tensile straining test of two AgNWs on PDMS (PDMS_T_02 and PDMS_T_03 from **Table S3**). The straining direction of the substrate is indicated in yellow. Both wires exhibit the onset of tensile failure at a tensile substrate strain of 3.21%, similar to the behavior observed in the wire shown in **Figure 2a**.

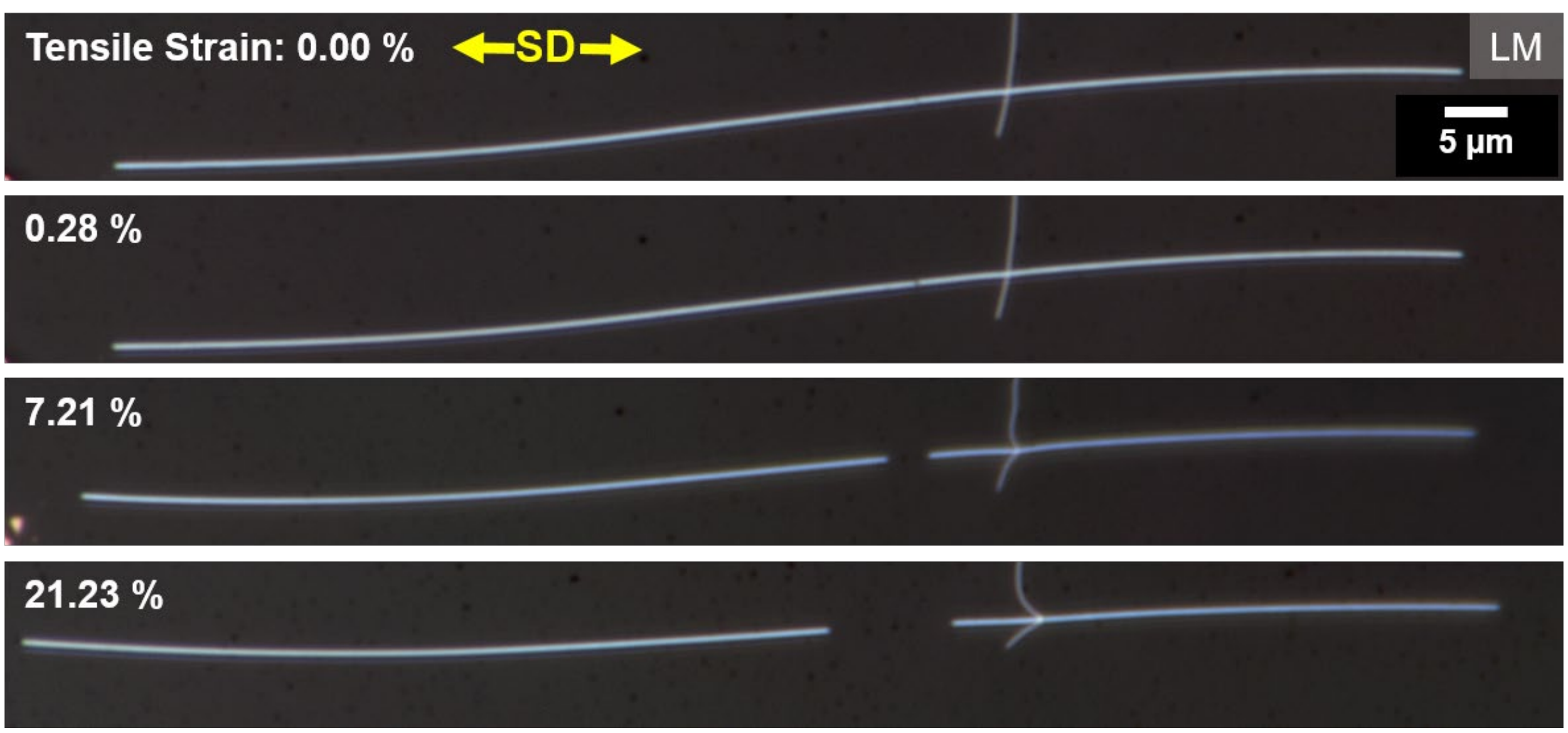

Figure S 20. LM still frames from an *in situ* tensile straining test of a long AgNWs on PDMS (PDMS_T_04 from **Table S3**). The straining direction of the substrate is indicated in yellow. The wire shows fracture at a comparatively low value of tensile substrate strain of 0.28 %**.**

Table S4. Initial length of all compressively tested AgNWs on PET, the compressive substrate strain values at the first occurrence of plastic deformation (kink formation), and the number of plastic deformation sites at the end of straining.

Wire	Initial wire length [µm]	Compressive substrate strain at first plastic deformation [%]	Number of deformation sites
PET_C_01 (Figure 4a)	59.0	1.94	6
PET_C_02 (Figure 4b)	91.1	2.14	13
PET_C_03 (Figure S21)	50.1	2.35	4
PET_C_04 (Figure S22)	109.9	2.78	14
PET_C_05 (Figure S23)	70.9	1.51	11
PET_C_06 (Figure S24)	71.8	1.51	10
PET_C_07 (Figure S25)	35.1	2.13	4
PET_C_08 (Figure S26)	74.2	2.21	9

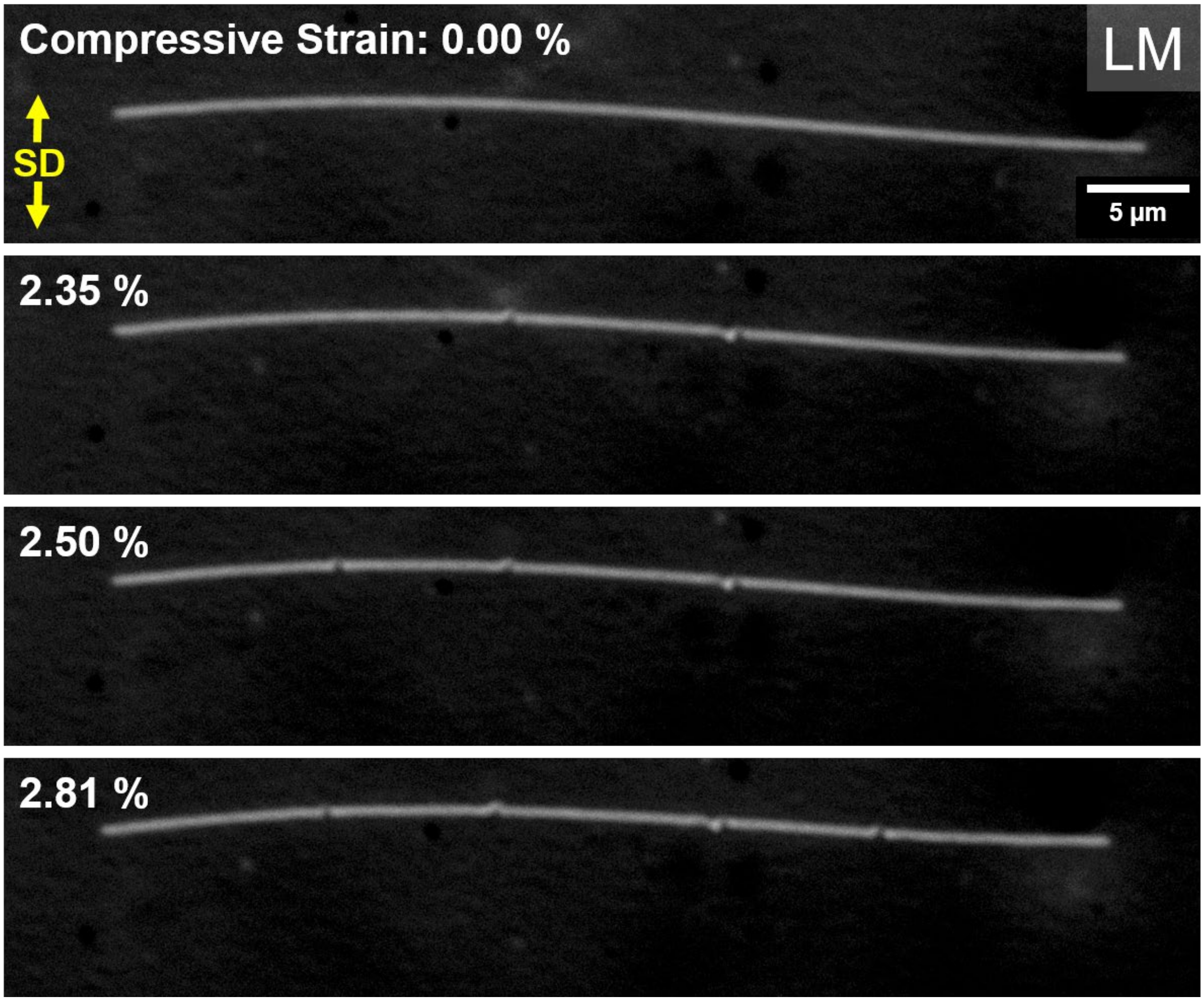

Figure S21. LM still frames from an *in situ* compression test of an AgNW on PET (PET_C_03 from **Table S4**). The straining direction of the substrate is indicated in yellow. The wire shows the characteristic onset of plastic deformation (kink formation) at 2.35 % compressive substrate strain and displays a total of four plastic deformation sites at the end of the experiment.

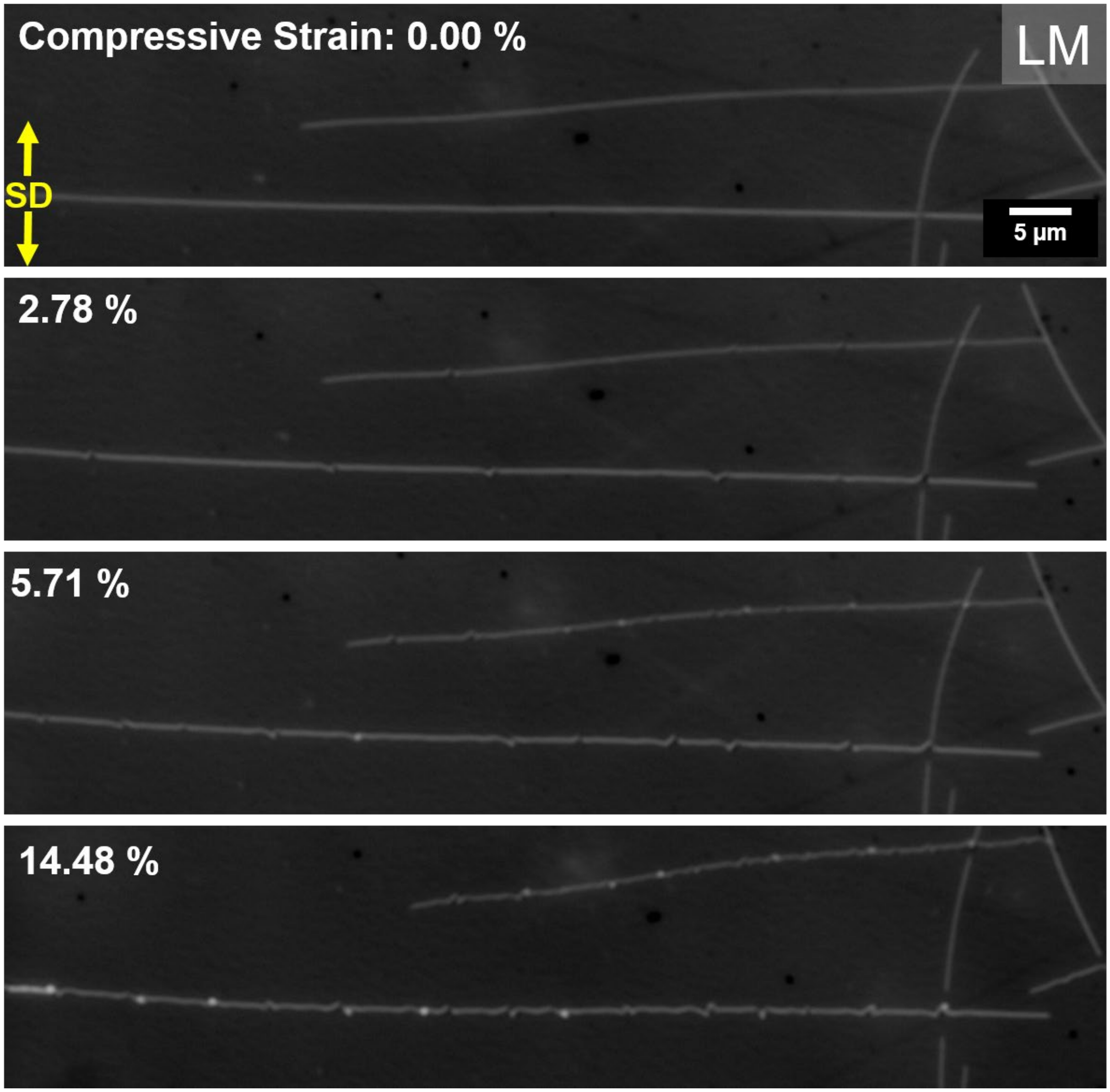

Figure S22. LM still frames form an *in situ* compression test of an AgNW located on PET (PET_C_04 from **Table S4**). The straining direction of the substrate is indicated in yellow. The wire shows the characteristic onset of plastic deformation (kink formation) 2.78 % compressive substrate strain and displays a total of 14 plastic deformation sites at the end of the experiment.

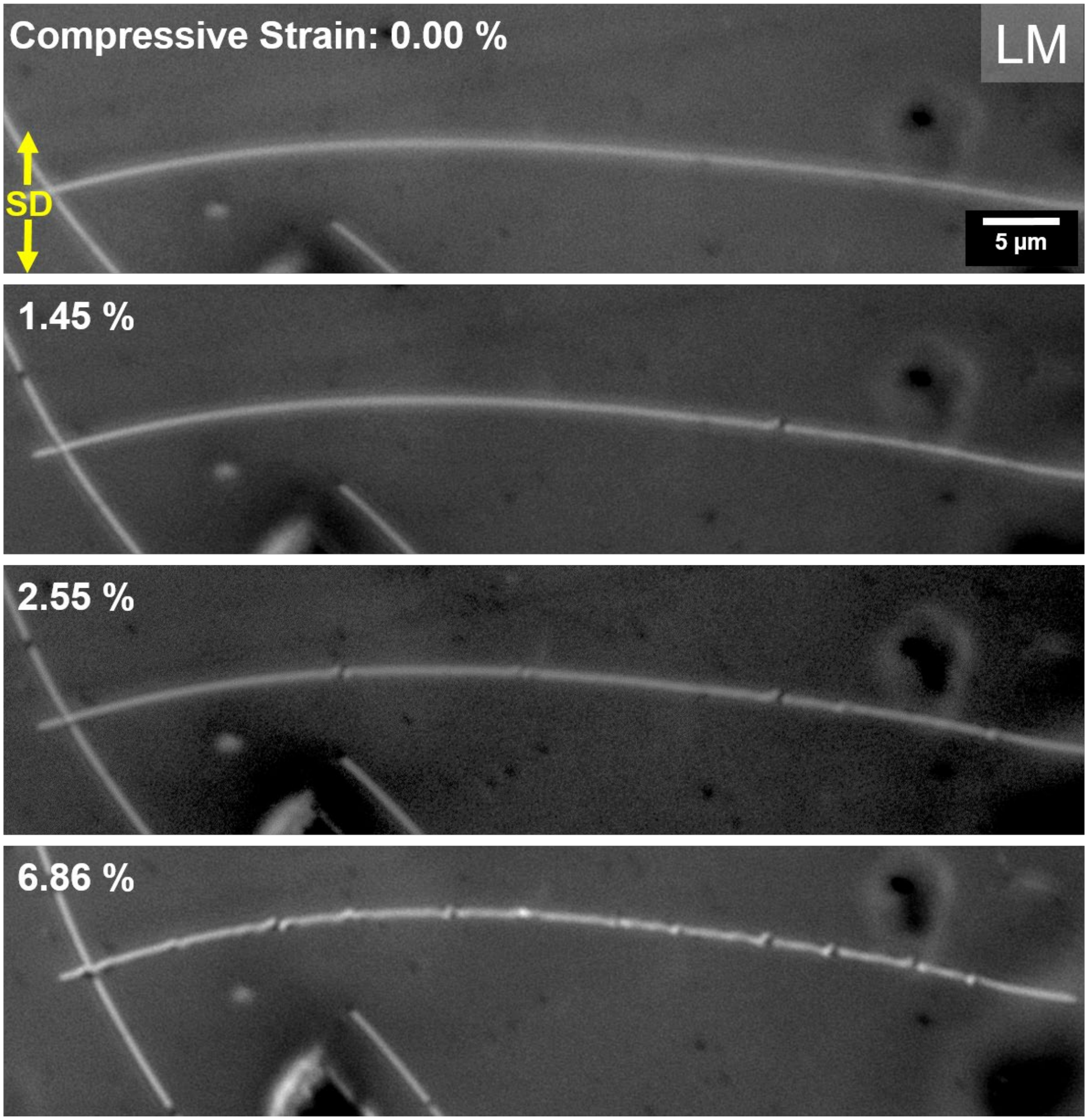

Figure S23. LM still frames form an *in situ* compression test of an AgNW located on PET (PET_C_05 from **Table S4**). The straining direction of the substrate is indicated in yellow. The wire shows the characteristic onset of plastic deformation (kink formation) at 1.45 % compressive substrate strain and displays a total of 11 plastic deformation sites at the end of the experiment.

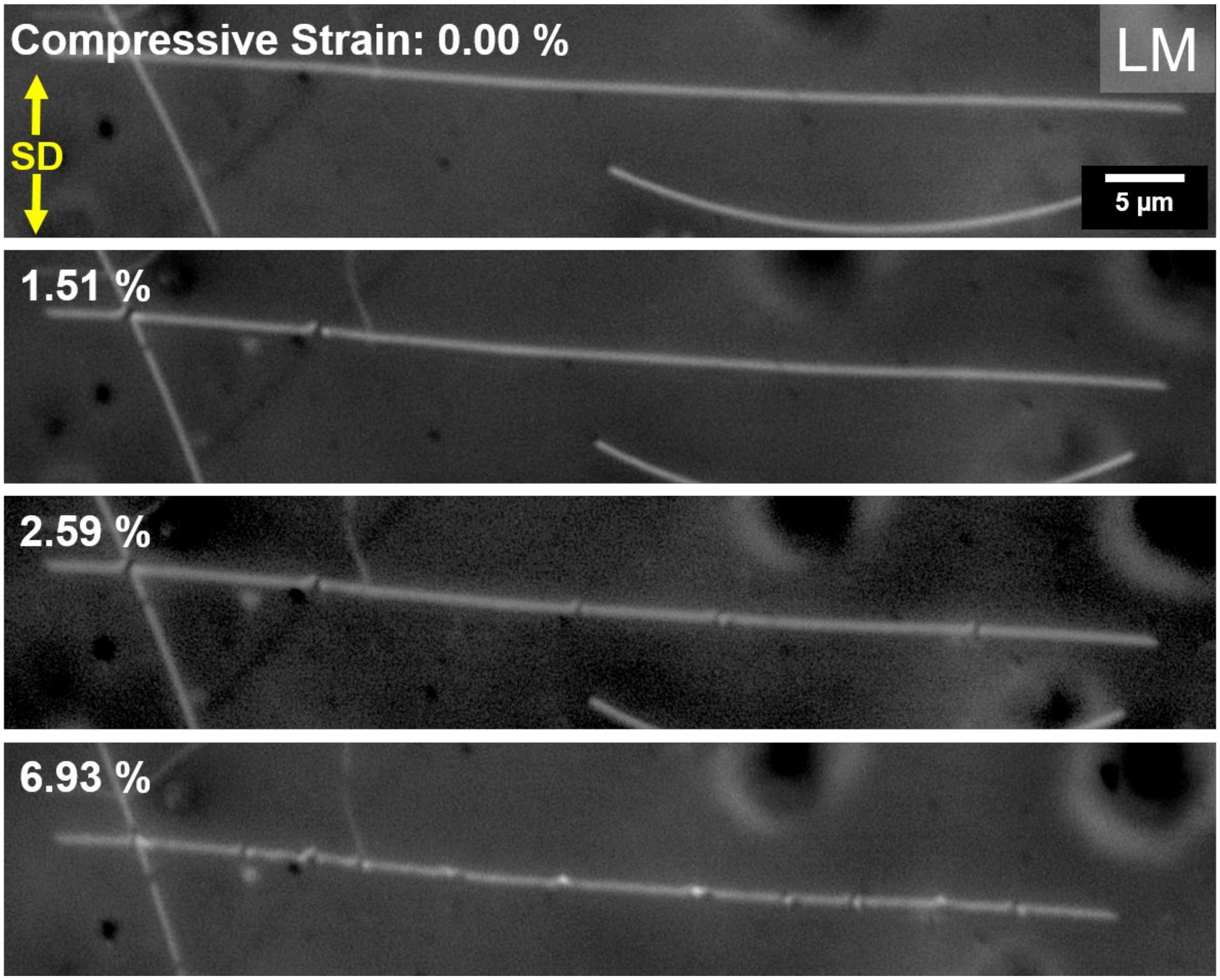

Figure S24. LM still frames form an *in situ* compression test of an AgNW located on PET (PET_C_06 from **Table S4**). The straining direction of the substrate is indicated in yellow. The wire shows the characteristic onset of plastic deformation (kink formation) at 1.51 % compressive substrate strain and displays a total of 10 plastic deformation sites at the end of the experiment.

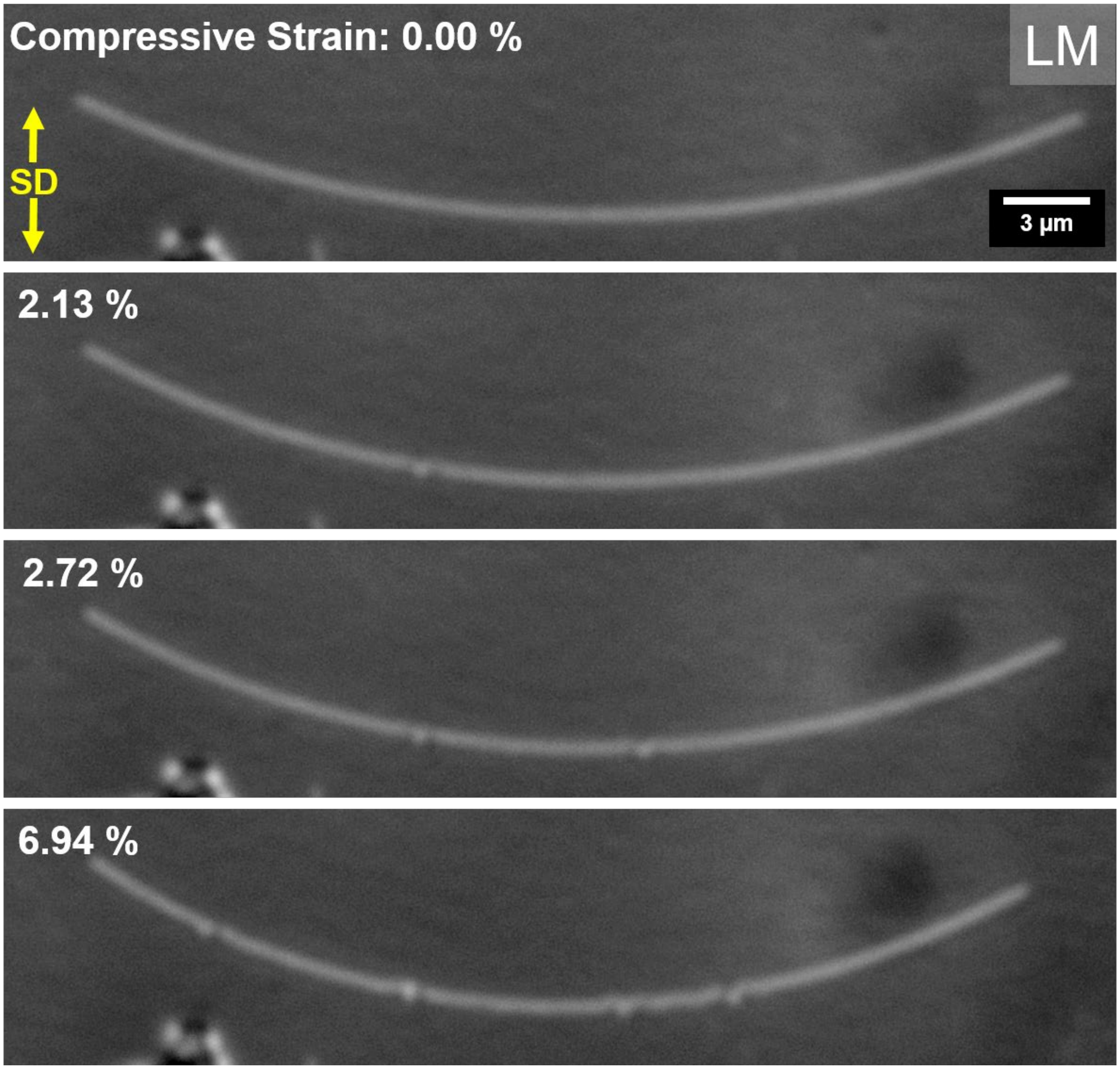

Figure S25. LM still frames form an *in situ* compression test of an AgNW located on PET (PET_C_07 from **Table S4**). The straining direction of the substrate is indicated in yellow. The wire shows the characteristic onset of plastic deformation (kink formation) at 2.13 % compressive substrate strain and displays a total of four plastic deformation sites at the end of the experiment.

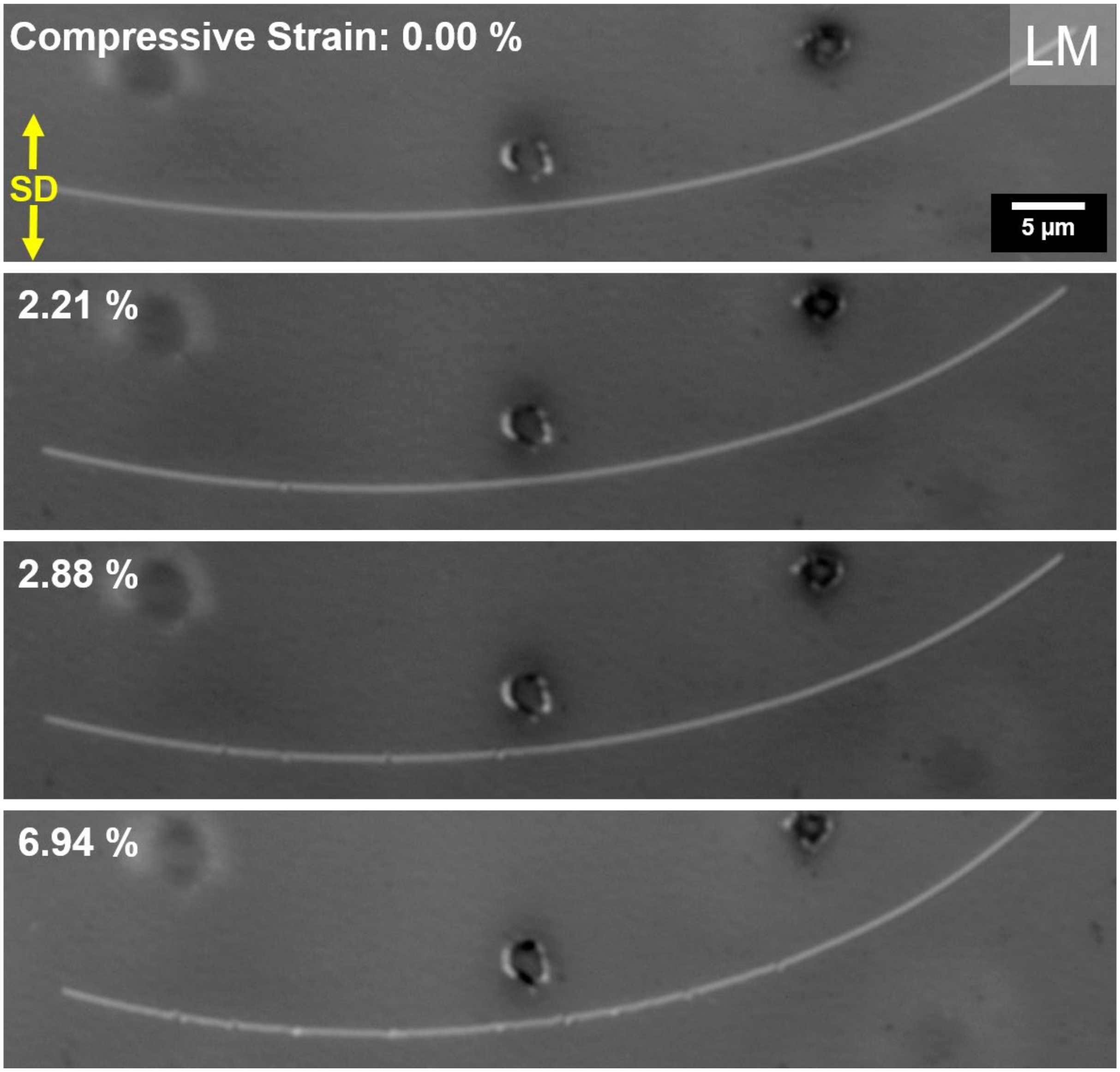

Figure S26. LM still frames form an *in situ* compression test of an AgNW located on PET (PET_C_08 from **Table S4**). The straining direction of the substrate is indicated in yellow. The wire shows the characteristic onset of plastic deformation (kink formation) at 2.21 % compressive substrate strain and displays a total of nine plastic deformation sites at the end of the experiment.

Table S5. Compression tests of AgNWs on PDMS. For each tested wire, the table lists: the number of buckling periods; the critical compressive substrate strain at which the first plastic deformation (kink formation) occurs; the number of kinks formed along the wire; and the critical tensile strain at the wire surface at the elastic-to-plastic transition, evaluated for each kink using the procedure described in the main manuscript**.**

Wire	Initial wire length [µm]	Number of periods	Compressive substrate strain at first plastic deformation [%]	Number of kinks	Critical tensile strain at wire surface at elastic-to-plastic transition [%]
PDMS_C_01 (Figure7)	69.5	20	7.76	6	2.86/3.00/3.66/ 3.35/3.32/2.90
PDMS_C_02 (Figure8)	13.6	5	8.45	1	4.12
PDMS_C_03 (Figure S28)	34.9	12	4.03	3	3.48/3.64/3.83
PDMS_C_04 (Figure S29)	62.4	21	6.10	5	3.12/3.40/3.60/3.24/3.43
PDMS_C_05 (Figure S30)	18.5	3	8.65	1	2.70
PDMS_C_06 (Figure S31)	62.6	18	8.89	3	3.22/2.59/2.51
				**Mean**	**3.25 ± 0.42**

In addition to the values reported in **Table S5**, we have calculated the mean period length from all deformation sites exhibiting a transition from elastic to plastic deformation. The resulting critical period length is 4.3 ± 0.7 µm.

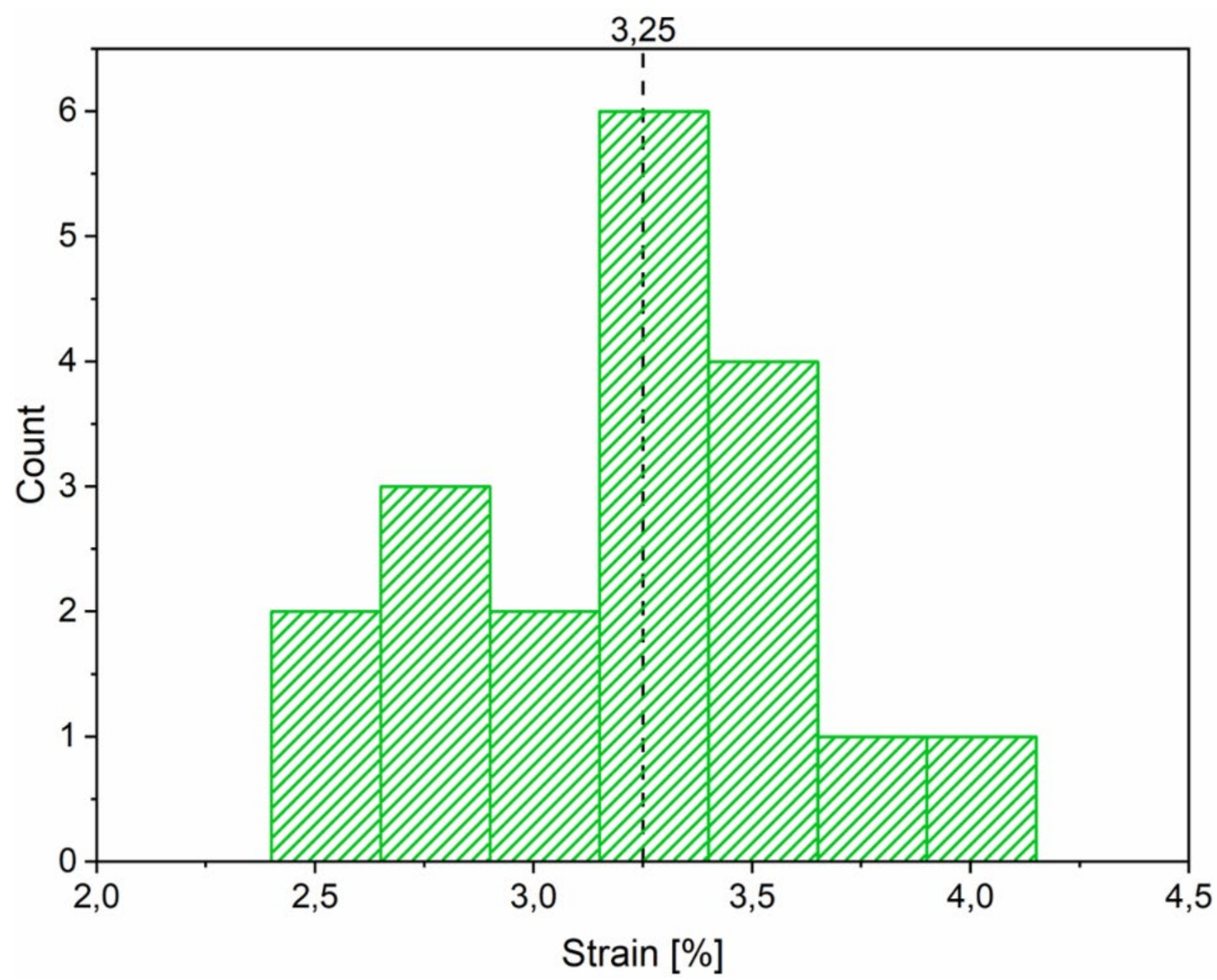

Figure S 27. Histogram of critical tensile strain values (at the convex surface of the AgNWs) at elastic-to-plastic transition, evaluated for the kinks formed in compression tests on PDMS (cf. **Table S4**, last column). The vertical dashed line indicates the mean critical tensile strain value.

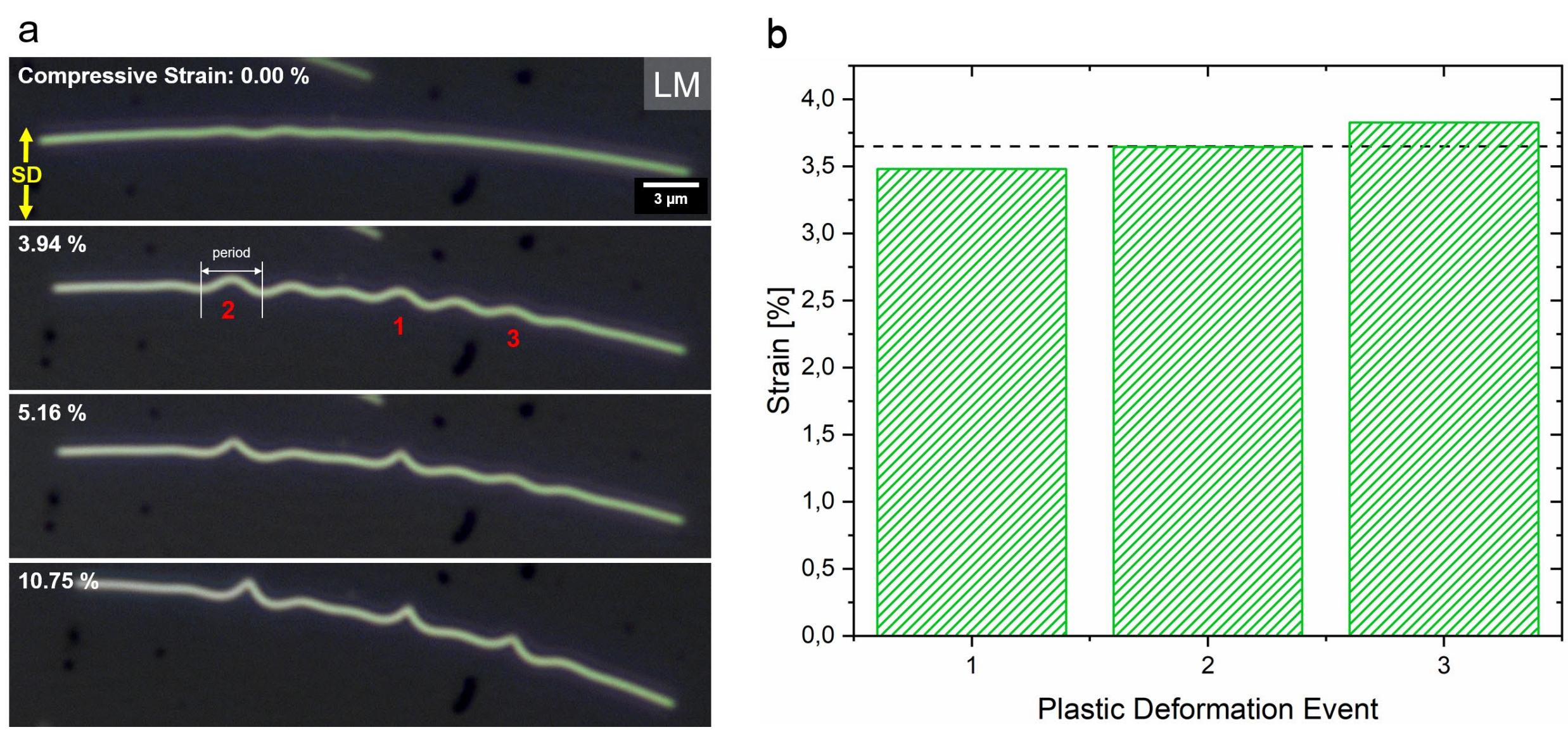

Figure S28. LM images from an in situ compression test of a wire on PDMS (PDMS_C_03 in **Table S5**) including analysis of the strain at the onset of plastic deformation. Straining direction of the substrate is indicated in yellow. An example definition of one period is added in white. a) LM still frames from an in situ straining test showing the onset of plastic deformation at 5.16 %

of compressive substrate strain. b) Tensile strain on the convex wire surface calculated from the wire curvature before plastic deformation as illustrated in **Figure 7** and **Figure S7**.

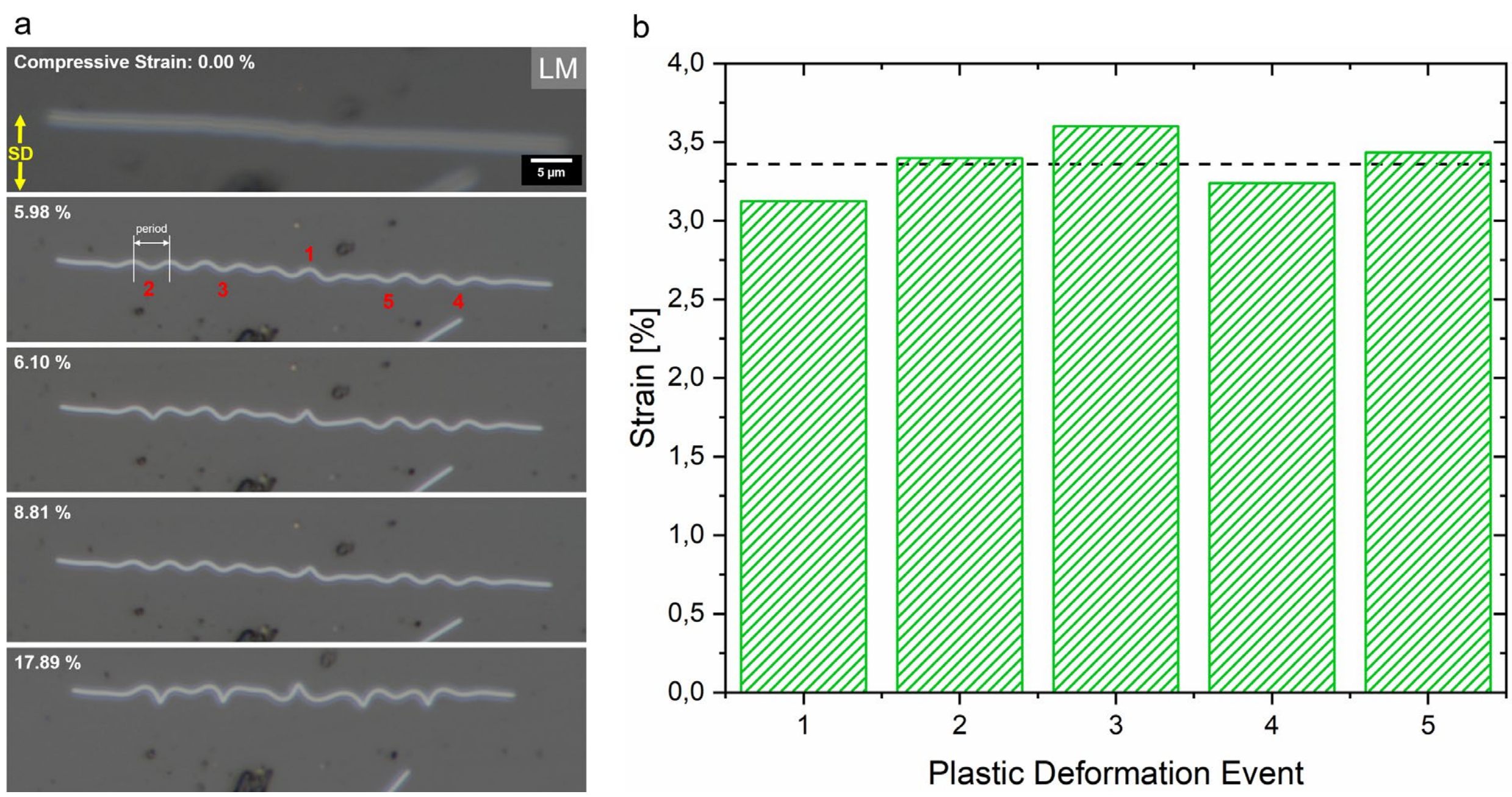

Figure S29. LM images from an in situ compression test of a wire on PDMS (PDMS_C_04 in **Table S5**) including analysis of the strain at the onset of plastic deformation. Straining direction of the substrate is indicated in yellow. An example definition of one period is added in white. a) LM still frames from an in situ straining test showing the onset of plastic deformation at 6.10 % of compressive substrate strain. b) Tensile strain on the convex wire surface calculated from the wire curvature before plastic deformation as illustrated in **Figure 7** and **Figure S7**.

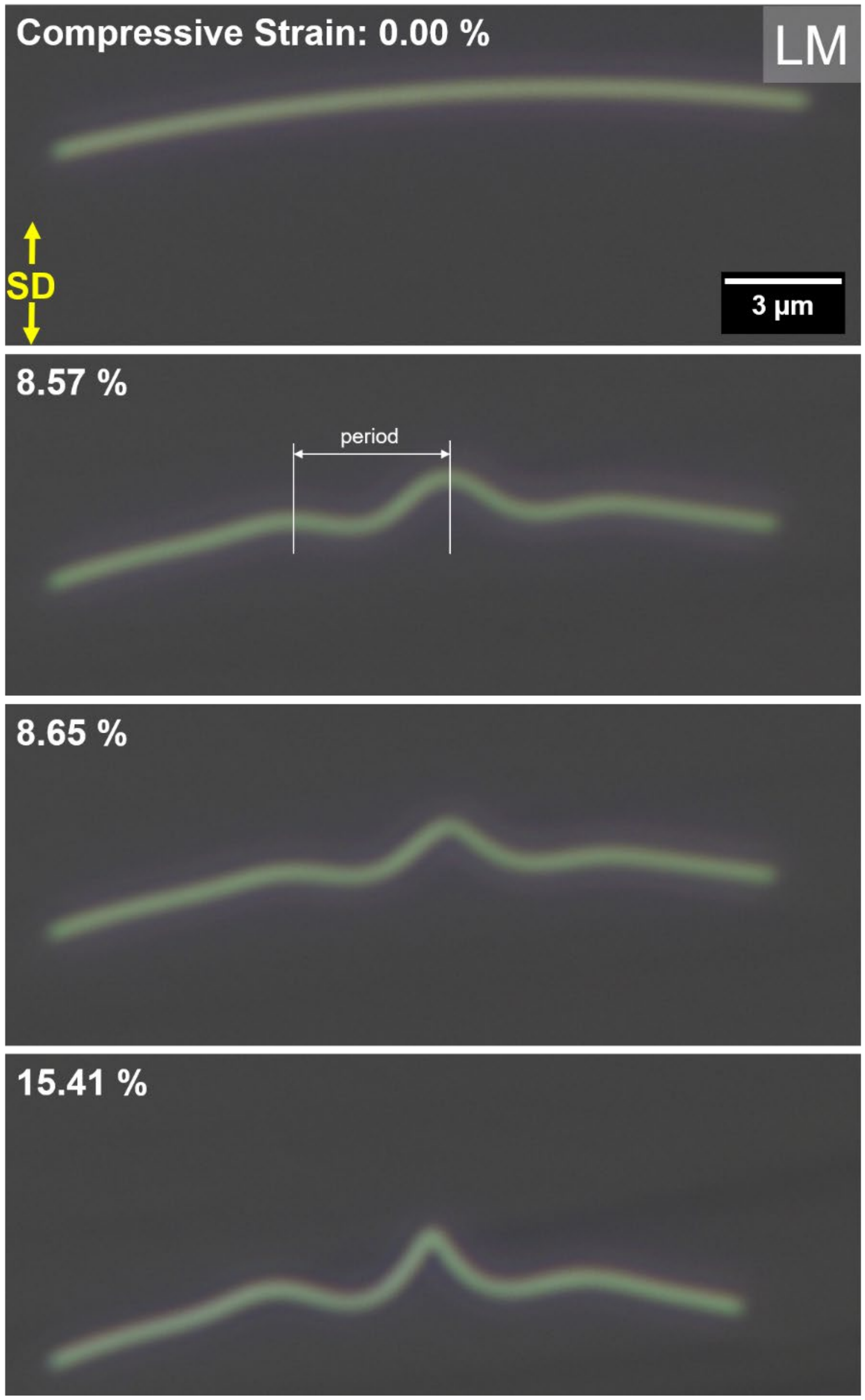

Figure S30. LM images from an in situ compression test of a wire on PDMS (PDMS_C_05 in **Table S5**) including analysis of the strain at the onset of plastic deformation. Straining direction of the substrate is indicated in yellow. An example definition of one period is added in white. a) LM still frames from an in situ straining test showing the onset of plastic deformation at 8.65 % of compressive substrate strain. Tensile strain on the convex wire surface calculated from the wire curvature before plastic deformation was 3.35 %, calculated as illustrated in **Figure 7** and **Figure S7**.

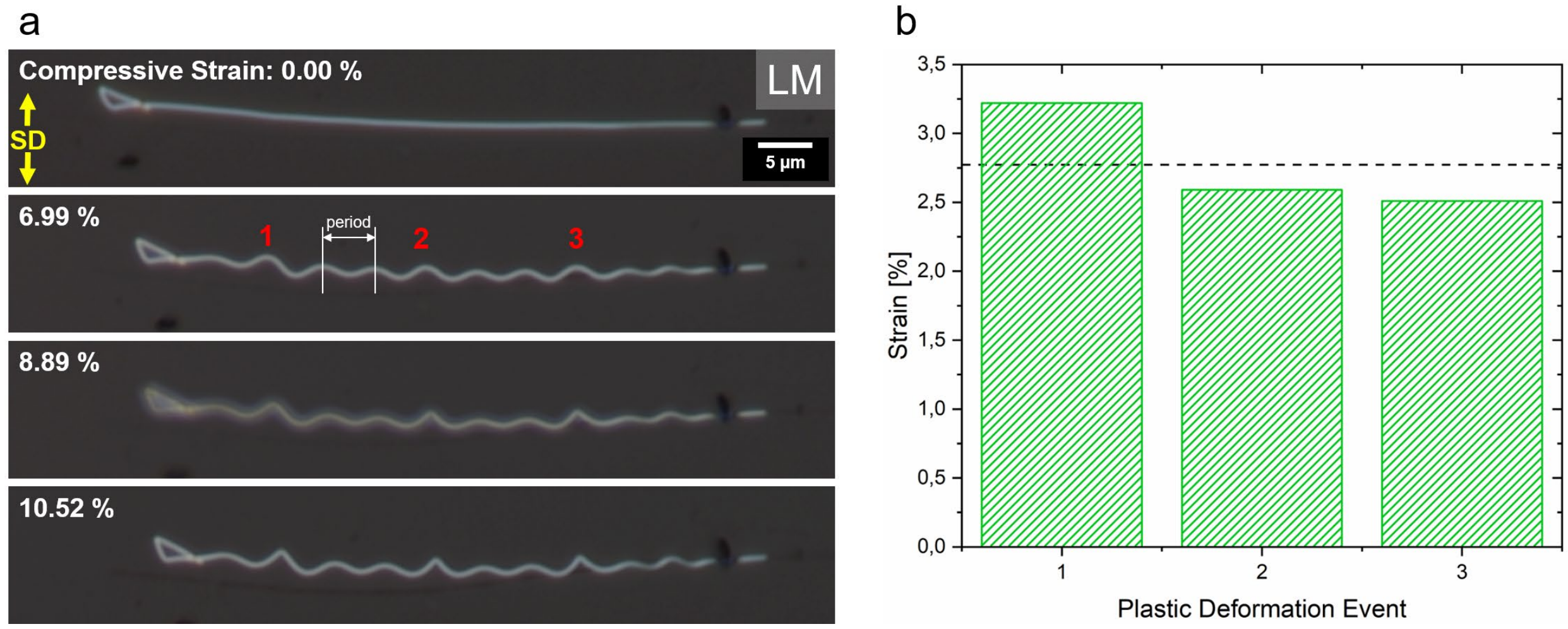

Figure S31. LM images from an *in situ* compression test of a wire on PDMS (PDMS_C_06 in **Table S5**) including analysis of the strain at the onset plastic deformation. Straining direction of the substrate is indicated in yellow. An example definition of one period is added in white. a) LM still frames from an *in situ* straining test showing the onset of plastic deformation at 8.89 % of compressive substrate strain. b) Strain on the wire calculated from the wire curvature before plastic deformation as illustrated in **Figure 7** and **Figure S7**.

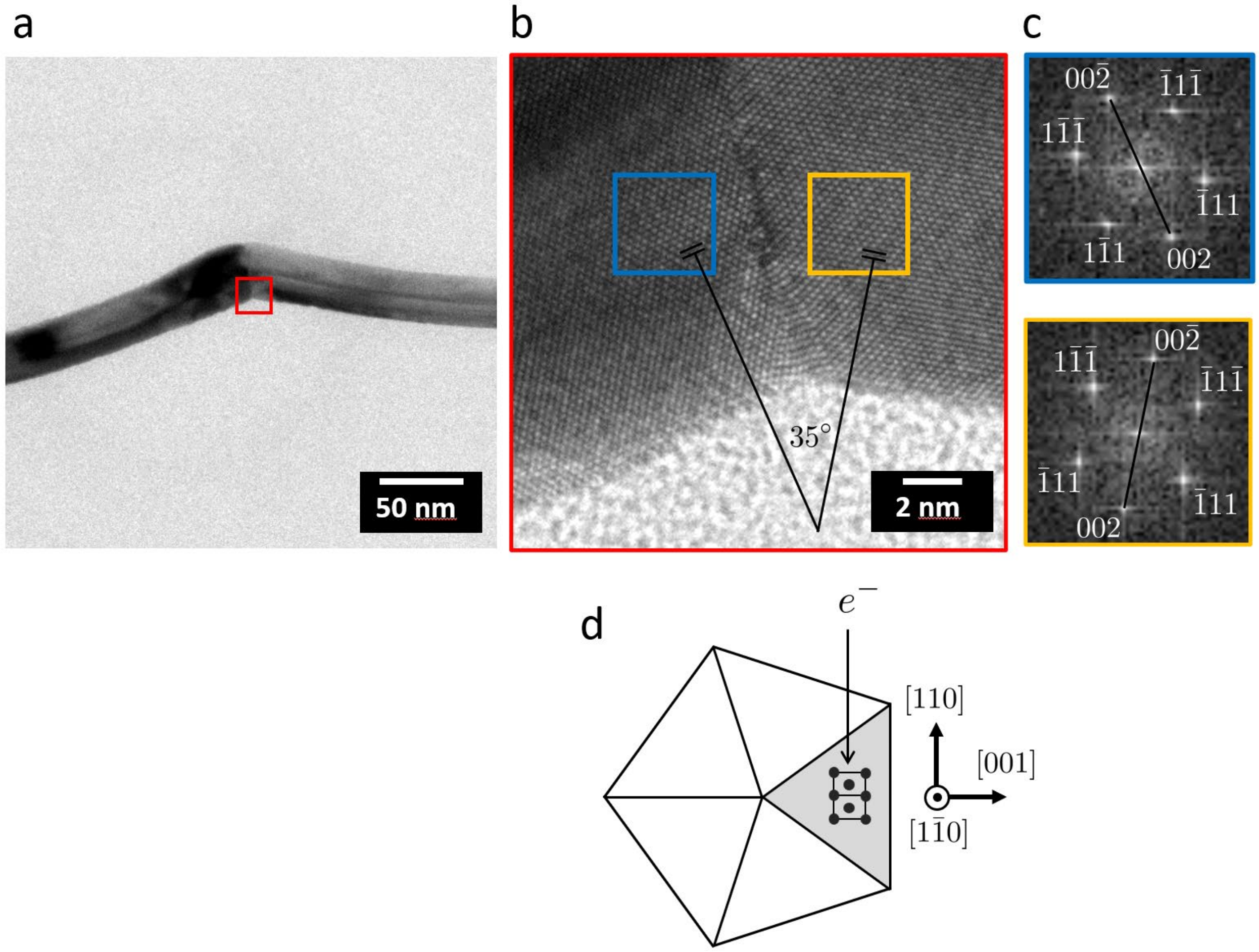

Figure S32. High-resolution TEM analysis of a grain boundary in a single nanowire kink. a) Conventional TEM bright-field image of a nanowire kink. b) High-resolution TEM image of the inner region of the kink, revealing the Ag crystal lattice in the [110] projection for both nanowire segments, which are separated by a tilt grain boundary. Local fast Fourier transforms (FFT) are used to determine the grain boundary tilt angle, measured to be 35°. d) Schematic illustration of the nanowire orientation in the HRTEM experiment, with the twin segment (grey) giving rise to the high-resolution lattice contrast. The crystallographic directions are used to index the local diffractograms shown in c). Note that the grain boundary in this twin segment appears highly localized but exhibits a surface reconstruction in the form of a triangular defect, as discussed in Ref. [4].

Supplementary Movies

Movie 1. Full video of the tensile test performed in **Figure 2a** of a AgNW on a PDMS substrate showing on fracture

Movie 2. Full video of the tensile test performed in **Figure 2b** of an AgNW on a PET substrate showing fragmentation into segments of almost equal length

Movie 3. Full video of the compressive straining test performed in **Figure 4a** of an AgNW on a PET substrate illustrating the formation of deformation events with strain increase

Movie 4. Full video of the compressive straining test performed in **Figure 4b** on an AgNW on a PET substrate illustrating the folding of deformations events by measuring the angular changes of two deformation spots

Movie 5. Full video of the cyclic compressive straining test performed in **Figure 7a** on an AgNW on a PDMS substrate illustrating the transition from elastic to plastic deformation.

Movie 6. Full video of the cyclic compressive straining test performed in **Figure 8b** on an AgNW on a PDMS substrate showing the process of repeated loading on plastic deformation spots.

References

(1) Goodfellow. PET Data Sheet.

(2) The Dow Chemical Company. SYLGARD™ 184 Silicone Elastomer. *Silicone Elastomer Technical Data Sheet*. 2017.

(3) Schweizer, P. Manipulation of Individual Defects in 2D and Layered Materials, University Erlangen-Nuernberg, 2019.

(4) Schrenker, N.; Xie, Z.; Schweizer, P.; Moninger, M.; Werner, F.; Karpstein, N.; Mackovic, M.; Spyropoulos, G.; Göbelt, M.; Christiansen, S.; Brabec, C.; Bitzek, E.; Spiecker, E. Microscopic Deformation Modes and Impact of Network Anisotropy on the Mechanical and Electrical Performance of Fivefold Twinned Silver Nanowire Electrodes. *ACS Nano* **2021**. https://doi.org/10.1021/acsnano.0c06480.
